# Supplementary material for: Alkali cation-π interactions in aqueous systems, modulating supramolecular stereoisomerism of nanoscopic metal-organic capsules
Source: Nat Commun. 2024 Nov 23;15:10180. doi: 10.1038/s41467-024-54426-4 (PMC11585540; doi:10.1038/s41467-024-54426-4)
Supplement: Supplementary file 1 — Supplementary Information [file 41467_2024_54426_MOESM1_ESM.pdf]

Supplementary Information

Alkali Cation-π Interactions in Aqueous Systems, Modulating Supramolecular Stereoisomerism of Nanoscopic Metal-Organic Capsules

Paul Wix[1], Swetanshu Tandon[1], Sebastien Vaesen[1], Kadri Karimu[1], Jennifer S. Mathieson[2], Kane Esien[3], Solveig Felton[3], Graeme W. Watson[1] and Wolfgang Schmitt[1*]

[1] School of Chemistry & SFI AMBER Research Centre, Trinity College Dublin, the University of Dublin, College Green, Dublin, D02 PN40, Ireland
[2] School of Chemistry, University of Glasgow, Joseph Black Building, University Ave, Glasgow G12 8QQ, United Kingdom (UK)
[3] Centre for Quantum Materials and Technologies, School of Mathematics and Physics, Queen's University Belfast, BT7 1NN, Belfast, United Kingdom (UK)

*Correspondence to: schmittw@tcd.ie

	Page
Supplementary Note 1. Synthetic Protocols	2-7
Supplementary Note 2. Anal. & Comp. Methods	8-10
Supplementary Figure 1. IR Spectra	11-12
Supplementary Figure 2. Alkali Metal Bond Distances	13-15
Supplementary Figures 3-8. Packing Diagrams	16-20
Supplementary Figure 9. DFT Energy Minimizations	21
Supplementary Figure 10. MEP Maps	22
Supplementary Figures 11-32. Mass Spectra	23-41
Supplementary Figure 33. XRD Patterns	42-43
Supplementary Figure 33. SQUID Data	44
Supplementary Tables 1-8. MS Assignments	45-52
Supplementary Tables 9-15. BVS Analyses	53-54
Supplementary Tables 16-20. Crystallographic Details	55-59
Supplementary References	60-61

Supplementary Notes

Supplementary Note 1. Synthetic Procedures

***Synthesis tetraethyl-1,3-benzenedisphosphonate*[1]**

10 g of 1,3-dibromobenzene (42.4 mmol, 1 eq) was dissolved in 20 mL 1,3-diisopropylbenzene under a nitrogen atmosphere and stirred at 180 °C for 30 minutes. The temperature of the solution was lowered to 120 °C and 1.17 g $NiBr_2$ (5.4 mmol, 0.13 eq) were added to the mixture after which the solution was heated up again to 180 °C for 20 minutes. 25 mL triethyl phosphite (24.2 g, 133.0 mmol, 3.14 eq) in 20 mL of 1,3-dibromobenzene were slowly dropped in the solution over the next 5 hours. A steady stream of nitrogen gas over the solution was maintained during the reaction to remove ethyl bromide from the atmosphere. The reaction was stirred at 180 °C for 24 hours before cooled to room temperature before triethyl phosphite, solvent and other by-products were distilled of under a high vacuum. The residue was purified by column chromatography with an eluent of DCM:MeOH (20:1). The product was obtained as a slightly yellow oil. Yield: 10.8 g (30.8 mmol, 72.7 %) ^{1}H-NMR ($CDCl_3$, 400 MHz) σ_H (ppm): 8.26 (1H, t, ^3J = 13.19 Hz), 7.99 – 8,05 (2H, m), 7.57 – 7.63 (1H, m), 4.10 – 4.23 (8H, m), 1.36 (12H, t, ^3J = 7.69 Hz), ^{31}P-NMR ($CDCl_3$, 162 MHz) σ_P (ppm): 16.92 (s).

Synthesis of benzene-1,3-diphosphonic acid (H_4BDP)

10.8 g (30.8 mmol, 1 eq) of tetraethyl-1,3-benzenedisphosphonate were dissolved in 50 mL half-concentrated hydrochloric acid and refluxed overnight. The solution was filtered and the solvent removed under vacuum. The product was obtained as a white powder (6.94 g, 94.6 %). ^{1}H-NMR (D_2O+NaOD, 400 MHz) σ_H (ppm): 8.00 (1H, t, ^3J = 13.24 Hz), 7.81-7.87 (2H, m), 7.52 – 7.57 (1H, m). ^{13}C-NMR (D_2O+NaOD, 100 MHz) σ_c (ppm): 126.92 (t, ^3J = 24.71 Hz), 130.4 (m), 131.11 (t, ^2J = 9.51 Hz), 139.53 (dd, ^1J = 166.34 Hz, ^3J = 10.76 Hz). ^{31}P-NMR (D_2O+NaOD, 162 MHz) σ_P (ppm): 14.53 (s) FT-IR (ATR) $\tilde{v}$ (cm^{-1}): 1593 (w), 1402 (m), 1197 (w), 1152 (w), 1087 (m), 982 (s), 939(s), 845(m), 808 (m), 799 (m), 727(m), 693 (s), 675(s), 569 (m), 526(m). ESI-MS: $[M-H]^-$: calculated m/z: 293.0349, observed m/z: 293.0352.

***Synthesis of 5-tert-butyl-tetraethyl-1,3-benzenediphosphonate*[1]**

10 g of 5-*tert*-Butyl-1,3-dibromobenzene (34.2 mmol, 1 eq) was dissolved in 10 mL of 1,3-diisopropylbenzene under nitrogen. This mixture was stirred at 180 °C for 30 minutes after which 1.07 g of Nickel(II) bromide (4.90 mmol, 0.14 eq) was added. A solution of 20 mL of triethyl phosphite (20 mL, 117 mmol, 3.42 eq) in 8 mL of 1,3-diisopropylbenzene was slowly added in steps. After 1 mL was added, the reaction was allowed to stir for 30 minutes after which the rest of the triethyl phosphite solution was added dropwise over a period of 5 hours. The reaction mixture was stirred over 24hr before being cooled to room temperature. Triethyl phosphate, the solvent and other by-products of the reaction were removed under vacuum. The residue was taken up in a 100 mL of ethyl acetate and washed three times with 100 mL of water each. The yellow oil was purified by column chromatography (Eluent: EtOAc:MeOH v/v 20:1). The product was retrieved as a yellow oil (10.58 g, 26.1 mmol, 76.31 %). ^{1}H-NMR ($CDCl_3$, 400 MHz) σ_H(ppm): 1.27-1.30 (12H, t, ^3J = 7.08 Hz), 1.31 (9H, s), 4.00 - 4.17 (8H, m), 7.93-7.99 (3H, m). ^{13}C-NMR ($CDCl_3$, 100 MHz) σ_C (ppm): 16.27 (t, ^3J = 3.15 Hz), 31.05 (s), 35.06 (m), 62.26-62.29 (m), 128.96 (dd, J_1 = 101.49 Hz, J_2 = 14.40 Hz), 131.94 (t, ^3J = 10.53 Hz), 132.47-132.55 (m), 151.94 (s). ^{31}P NMR ($CDCl_3$, 162 MHz) σ_p (ppm): 17.96 (s).

Synthesis of 5-tert-butyl-1,3-benzenediphosphonic acid (H_4BDP-tBu)

A mixture of 10.58 g 5-*tert*-butyl-tetraethyl-1,3-benzenediphosphonate (26.0 mmol, 1eq) and 80 mL of half-concentrated hydrochloric acid (40 mL) was refluxed overnight. The solution was filtered and the filtrate removed under vacuum. The product was obtained as an off-white powder (7.12 g, 92 %). ^{1}H NMR (D_2O+NaOD, 400 MHz) σ_H (ppm): 3.52 (9H, s), 4.71-4.72 (4H,m), 10.01 – 10.11(3H, dd, J=14.4883 Hz). ^{13}C NMR (D_2O+NaOD, 100 MHz) σ_C (ppm): 31.43 (s), 35.01 (s), 130.59 (s), 133.3 (s), 135.29 (s), 150.51-150.64 (m). ^{31}P NMR (D_2O+NaOD, 162 MHz) σ_p (ppm): 14.53. FT-IR(ATR) ṽ (cm^{-1}): 3585 (vw), 2973 (w), 1651 (w), 1584 (w), 1479 (w), 1412 (w), 1364 (w), 1092 (m), 1005 (s), 938 (s), 887 (m), 819 (m) 774 (m), 698 (s), 613 (w), 563 (m) 524(s). ESI-MS: [M-H]$^-$: calculated m/z: 236.9723, observed 236.9701.

Synthesis of DPB-P-OEt hexethyl-1,3,5-benzenetriphosphonate[1]

10 g of 1,3,5-tribromobenzene (31.8 mmol, 1 eq) were dissolved in 20 mL of 1,3-diisopropylbenzene under a nitrogen atmosphere and stirred at 180 °C for 30 minutes. 1 g of $NiBr_2$ (4.58 mmol, 0.14 eq) were added and the solution was stirred for another 20 minutes after which, 25 mL triethyl phosphite (24.2 g, 133.0 mmol, 4.00 eq) in 20 mL of 1,3-dibromobenzene were slowly dropped in the solution over the next 5 hours. A steady stream of nitrogen gas over the solution was maintained during the reaction to remove ethyl bromide from the atmosphere. The reaction was stirred at 180 °C for 24 hours before cooled to room temperature before triethyl phosphite, solvent and other by-products were distilled of under a high vacuum. The residue was purified by column chromatography with an eluent of DCM:MeOH (20:1). The product was obtained as a slightly yellow oil. Yield: 4.52 g (29.3 %).^{1}H-NMR ($CDCl_3$, 400 MHz) σ_H (ppm): 8.37 – 8.44 (3H, m), 4.10 – 4.26 (12H, m), 1.36 (18H, t, ^{3}J = 7.07 Hz). ^{31}P-NMR ($CDCl_3$, 162 MHz) σ_P (ppm): 15.34.

Synthesis of 1,3-5-benzenetriphosphonic acid (H_6TDP)

4.5 g of hexethyl-1,3,5-benzenetriphosphonate were dissolved in 50 mL of half-concentrated hydrochloric acid and refluxed overnight. After filtration the solvent was removed under vacuum and the product obtained as a white powder. Yield: (2.62 g, 89 %) ^{1}H-NMR (D_2O+NaOD, 400 MHz) σ_H (ppm): 8.06 – 8.12 (3H, m) ^{13}C-NMR (D_2O, 100 MHz) σ_c (ppm): 133.48 (tt, J_{CP} = 180.42 Hz, J_{CP} = 12.29 Hz), 134.76 – 134.08 (m). ^{31}P-NMR (D_2O+NaOD, 162 MHz) σ_P (ppm): 12.79 (s). FT-IR(ATR) ṽ (cm^{-1}): 1586 (m), 1416 (m), 1227 (w), 1178 (m), 1123 (m), 1065 (m), 992 (s), 932 (s), 891 (m), 734 (m), 684(s), 574 (w), 509 (s).

Synthesis tetraethyl-5-bromo-1,3-benzenediphosphonate[1]

1,3,5-Tribromobenzene (10 g; 31.85 mmol, 1 eq) was dissolved in 1,3-diisopropylbenzene (10 mL) and degassed with nitrogen. This mixture was stirred at 180 °C for 20 mins under a nitrogen atmosphere. To the reaction mixture, 1.07 g of Nickel(II) bromide(4.90 mmol, 0.15 eq) was added. A solution of 20 mL of triethyl phosphite (12 mL; 63.8 mmol, 2.1 eq) in 8 mL of diisopropyl benzene was added next. After 1 mL was added, the reaction was allowed to stir for 30 minutes after which the rest of the triethyl phosphite solution was added dropwise over a period of 5 hours. The reaction mixture was stirred for 24hr before being cooled to room temperature. Triethyl phosphite, solvent and other by-products were removed under high vacuum. The residue was purified by column chromatography

with an eluent of EtOAc:MeOH (20:1). The product yielded as a yellow oil. Yield: 2.77 g, 20 % ^{1}H NMR ($CDCl_3$, 400 MHz) σ_H (ppm): 1.37 (12H, t, ^{3}J = 7.07 Hz), 4.09 - 4.26 (8H,m), 8.10 - 8.19 (3H, m). ^{13}C NMR ($CDCl_3$, 100 MHz) σ_C (ppm): 16.31 - 16.34 (m), 62.71 - 62.77 (m), 123.26 (s), 130.90-131.03 (m), 132.91 - 133.10 (m), 138.13 - 138.16 (m). ^{31}P NMR ($CDCl_3$, 162 MHz) σ_p (ppm): 14.62.

Synthesis of 5-bromo-1,3-benzenediphosphonic acid

2.77 g of tetraethyl-5-bromo-1,3-benzenediphosphonate (6.45 mmol) was refluxed in 20 mL of half-concentrated hydrochloric acid overnight. The solution was filtered and the filtrate evaporated under vacuum. The product was obtained as an off-white powder (2.03 g, 99 %). ^{1}H NMR (D_2O+NaOD, 400 MHz) σ_H (ppm): 7.87-7.89(3H, m). ^{13}C NMR (D_2O+NaOD, 100 MHz) σ_C (ppm): 122.68 (d, J = 17.71 Hz), 130.60 (t, ^{3}J = 10.47 Hz), 135.79 (d, 3J = 8.21Hz), 129.62 (dd, J1 = 178.20 Hz, J2 = 13.21Hz). ^{31}P NMR (D_2O+NaOD, 162 MHz) σ_p (ppm): 11.75. FT-IR(ATR) ṽ (cm^{-1}): 2737 (br), 2270 (br), 1581 (vw), 1564 (vw), 1413 (vw), 1394 (w) 1114 (m), 1084 (s), 1101 (s), 988 (s), 933 (s), 878 (m) 780 (m), 766 (m), 677 (s), 556 (m), 540 (m), 524 (s), 512 (m). ESI-MS: $[M-H]^-$: calculated m/z: 314.8840, observed m/z: 314.8828.

***Synthesis of tetraisopropyl-2,6-pyridinediphosphonate*[2]**

3 g of 2,6-dibromopyridine (12.7 mmol, 1 eq), 5 g of diisopropyl phosphite (5.1 mL, 30.4 mmol, 2.4 eq), 4.26 g of ethyldiisopropyl amine (5.74 mL, 32.9 mmol, 2.60 eq), 57 mg of palladium(II)acetate (0.253 mmol, 0.02 eq) and 154 mg of bis(diphenylphosphino)ferrocene (0.279 mmol, 0.02 eq) were dissolved under a nitrogen atmosphere in 12 mL of acetonitrile and refluxed for 24 hours. The solution was filtered and the residue extracted three times with ethyl acetate. The residue was purified by column chromatography with DCM as the eluent. The product was obtained as a yellow oil. Yield: 1.1 g (25 %) ^{1}H-NMR ($CDCD2Ol_3$, 400 MHz) σ_H (ppm): 1.35 (24H, dd, ^{2}J = 54.15 Hz, ^{3}J = 6.19 Hz), 4.75 – 4.83 (4H, m) 8.47 (dt, 2H, ^{3}J = 13.2 Hz, J_{HP} = 6.19 Hz), 9.09 – 9.12 (1H, m). ^{31}P-NMR ($CDCl_3$, 162 MHz) σ_P (ppm): 12.1 (s).

Synthesis of pyridine-2,6-diphosphonic acid (H_4BDP-Py)

1.1 g of tetraisopropyl-2,6-pyridinediphosphonate was dissolved in 20 mL CH_2Cl_2 under nitrogen and treated with 2.5 mL of trimethylsilyl bromide (2.90 g, 18.9 mmol, 7.02 eq). The mixture was stirred for 2 days, after which it was treated with 20 mL of water and stirred for another hour. The solution was evaporated under vacuum and the product obtained as an off white powder. Yield: 0.352 g (54 %) ^{1}H-NMR (D_2O+NaOD, 400 MHz) σ_H (ppm): 8.11 (tt, 1H, ^{3}J = 11.1 Hz, J_{HP} = 1.87 Hz), 8.54 – 8.57 (2H, m)^{13}C-NMR (D_2O+NaOD, 100 MHz) σ_c (ppm): 135.29 (dd, J = 163.88 Hz, J = 8.27 Hz), 140.13 (t, J = 7.33 Hz), 149.67 (d, J = 11.88 Hz). ^{31}P-NMR (D_2O+NaOD, 162 MHz) σ_P (ppm): 8.25. FT-IR(ATR) ṽ (cm^{-1}): 3301 (w), 3102 (m), 2660 (m), 2077 (w), 1619 (m), 1400 (m), 1204 (w), 1124 (m), 931 (s), 860 (m), 673 (s), 525 (s).

***Synthesis of tetrakisisopropyl-3,5-aniline-diphosphonate*[3]**

2 g of 3,5-dibromoaniline (7.97 mmol, 1 eq) together with 0.179 mg of palladium(II)acetate (0.797 mmol, 0.1 eq) and 105 mg of triphenylphosphine (0.398 mg, 0.05 eq) were combined under a nitrogen atmosphere. 5 mL of diisopropyl phosphite (4.99 g. 32.26 mmol, 4.8 eq) in 10 mL Ethanol and 5.12 mL of N,N'-dicyclohexylmethylamine (4.67 g, 23.9 mmol, 3 eq) in 10 mL Ethanol were added subsequently. The mixture was refluxed overnight, cooled down to room temperature and evaporated under nitrogen. The residue was purified *via* column chromatography using DCM/MeOH (10:1) as an eluent. Yield : 0.78 g (27 %). ^{1}H-NMR (DMSO-d^6, 400 MHz) σ_H (ppm): 1.23 (24H, dd, ^2J = 41.6 Hz, ^3J = 6.18 Hz), 4.50 – 4.55 (4H, m), 5.78 (2H, s), 7.08 – 7.12 (3H, m). ^{13}C-NMR (DMSO-d^6, 100 MHz) σ_c (ppm): 24.09 (d, J = 31.4 Hz), 70.58, 119.58 – 119.75 (m), 131.14 (dd, J = 185.17 Hz, J = 15.32 Hz, 149.42 (t, J = 16.16 Hz). ^{31}P-NMR (DMSO-d^6, 162 MHz) σ_P (ppm): 16.01.

Synthesis of 3,5-anilinediphosphonic acid (H_4BDP-NH_2)

0.780 g (1.85 mmol, 1 eq) of tetrakisisopropyl-3,5-aniline-diphosphonate was dissolved in 5 ml DCM under a nitrogen atmosphere and treated with 1.5 mL of trimethylsilyl bromide (1.74 g, 11.3 mmol, 6.14 eq). The mixture was stirred for 2 days after which it was treated with 10 mL of water. The solvents were removed under reduced pressure and the product obtained as an off-white powder. Yield: 0.298 g (63 %). ^{1}H-NMR (DMSO-d_6, 400 MHz) σ_H (ppm): 7.01 – 7.05 (2H, m), 7.22 (t, J = 12.73 Hz). ^{31}P-NMR (DMSO-d_6, 162 MHz) σ_P (ppm): 13.33. FT-IR(ATR) $\tilde{\nu}$ (cm^{-1}): 3375 (w), 2777 (br), 1683 (m), 1623 (m), 1585 (m), 1410 (m), 1278 (w), 1153 (m), 1132(m), 1077 (m), 996 (m), 932 (m), 917 (s), 882 (m), 810 (w), 714 (w), 683 (s), 599 (w), 583 (w), 539 (m), 503 (m).

Syntheses of M-{V_{20}} with M = Cs, Rb, K;

Cs-{V_{20}}: 0.070 g Na_3VO_4 (0.381 mmol, 1 eq), 0.070 g NaN_3 (1.08 mmol, 2.83 eq) and 0.193 g CsCl (1.14 mmol, 3 eq) were dissolved in 3 mL H_2O. In a second vial 0.050 g H_4DPB-H (0.210 mmol, 0.55 eq) was dissolved in 1 mL DMF. Both solutions were combined and 1 mL MeCN was added to the mixture. The yellow solution was heated to 70 °C before the pH value was adjusted to pH = 7 using concentrated, aqueous hydrochloric acid. After the addition of 16 µL of $N_2H_4{\cdot}H_2O$, the solution turned green and the pH value was readjusted to pH = 7. After stirring for 1 minute at 70 °C, the solution was cooled down to room temperature. The green solution was split to 1 mL samples, into which 0.5 mL MeCN was diffused into each sample. Green crystals formed after 2 days, which were filtered and dried at 40 °C overnight. Yield: 0.054 g. CHN analysis for $Cs_{11.5}(V_{20}P_{16}O_{84}Na_{0.5}Cs_8C_{48}H_{32})(CH_3CN)(H_2O)_{33}$; Expected: C: 8.96, H: 1.52, N: 0.21, Found: C: 9.15, H: 1.42, N: 0.23. Selected FT-IR signals (ATR-Diamond)/(cm^{-1}): 3306 (br), 2052 (w), 2032 (w), 1644 (br), 1468 (w), 1390 (w), 1110 (m), 1032 (m), 958 (s), 798 (m), 686 (m). 644 (m), 574 (m).

Rb-{V_{20}}: Rb-{V_{20}} was synthesized according to the procedure above, using 0.138 g RbCl (1.14 mmol, 3 eq) instead of CsCl. Yield: 0.048 g; CHN analysis for $Rb_{13}[V_{20}P_{16}O_{84}NaRb_8C_{48}H_{32}Cl_2](H_2O)_{30}$; Expected: C: 9.77, H: 1.57, N: 0.00, Found: C: 9.58, H: 1.71, N: 0.00. Selected FT-IR signals (ATR-Diamond)/(cm^{-1}): 3320 (br), 1646 (br), 1394 (w), 1118 (m), 1034 (m), 958 (s), 802 (m), 696 (m). 638 (m), 570 (m).

K-{V_{20}}: K-{V_{20}} was synthesized according to the procedure above, using 0.088 g K_3VO_4 (0.380 mmol, 1 eq) instead of Na_3VO_4 and CsCl. Yield: $3x10^{-2}$ g; CHN analysis for $K_{12}[V_{20}P_{16}O_{84}K_9C_{48}H_{32}](N_3)(H_2O)_{27}$; Expected: C: 11.96, H: 1.80, N: 0.87, Found: C: 11.91, H: 1.91, N: 0.99. Selected FT-IR signals (ATR-Diamond)/(cm^{-1}): 3324 (br), 2062 (m), 1644 (br), 1392 (w), 1106 (m), 1038 (m), 960 (s), 800 (m), 694 (m), 568 (m).

Syntheses of M-{V_{20}-*t*Bu} with M = Cs, Rb

Cs-{V_{20}-*t*Bu}: Cs-{V_{20}-*t*Bu} was synthesized analogously to **Cs-{V_{20}}**, substituting H_4BDP with 0.062 mg H_4BDP-*t*Bu (0.210 mmol, 0.55 eq). Yield: 0.012 g; CHN analysis for $Cs_{13}[V_{20}P_{16}O_{84}NaCs_8C_{80}H_{96}Cl_2] \cdot 26\ H_2O$: Expected: C: 13.22 H: 2.05 N: 0.00, Found: C: 13.57, H: 1.89, N: 0.09. Selected FT-IR signals (ATR-Diamond) /(cm^{-1}): 3364 (br), 2952 (m), 2870 (w), 1634 (br), 1464 (w), 1408 (w), 1366 (w), 1120 (s), 1028 (m), 962 (s), 819 (w), 703 (m), 652 (w), 570 (m).

Rb-{V_{20}-*t*Bu}: Rb-{V_{20}-*t*Bu} was synthesized analogously to **Cs-{V_{20}}**, substituting H_4BDP with 0.062 mg H_4BDP-*t*Bu (0.210 mmol, 0.55 eq). Yield: 0.01 g; CHN analysis for $Rb_{13}[V_{20}P_{16}O_{84}NaRb_8C_{80}H_{96}Cl_2] \cdot 29\ H_2O$: Expected: C: 15.18 H: 2.45 N: 0.00, Found: C: 15.31, H: 2.07, N: 0.09. Selected FT-IR signals (ATR-Diamond) / (cm^{-1}): 3352 (br), 2958 (m), 1644 (br), 1412 (w), 1365 (w), 1123 (s), 1038 (m), 965 (s), 823 (w), 702 (m), 575 (m).

Syntheses of Rb-{V_{20}-Py} and Rb-{V_{20}-NH_2}: Rb-{V_{20}-Py} and **Rb-{V_{20}-NH_2}** were synthesized analogously to **Rb-{V_{20}}** by substituting H_4BDP with 0.050 g H_4BDP-Py (0.210 mmol, 0.55 eq) or 0.053 g H_4BDP-NH_2 (0.210 mmol, 0.55 eq), respectively. The compounds crystallized as green crystals after several hours. The samples were product mixtures containing non-removable inorganic vanadate side-products, which prevented further characterization.

Syntheses of M-{V_{30}} with M = Cs, Rb, K

K-{V_{30}}: 0.070 g Na_3VO_4 (0.380 mmol, 1 eq) was dissolved in 3 mL of water and 1mL of MeCN together with 0.070 g NaN_3 (1.08 mmol, 2.83 eq) and 0.086 g KCl (1.14 mmol, 3 eq). In a second solution 0.066 g benzene-1,3,5-triphosphonic acid (H_6BTP) was dissolved in in 1mL DMF. Both solutions were combined and heated to 70 °C. Following 16 µL of $N_2H_4 \cdot H_2O$ was added to the solution, which subsequently turned green. The solution was stirred for one hour, during which a dark green precipitate formed. The solution was cooled down to room temperature before the colourless supernatant solution was removed. The precipitate was dissolved in 3 mL of water. Non-dissolved residuals were filtered of and 0.5 mL of DMF was added to the solution. Green crystals of **K-{V_{30}}** formed after several weeks, which were sorted by hand in the mother liquor. Yield: $4x10^{-2}$ g. CHN analysis for $K_{27}[V_{30}P_{24}O_{140}C_{48}H_{24}K_4]N_3 \cdot 74\ H_2O$; Expected: C: 7.49, H: 2.25, N: 0.55, Found:. C: 6.73, H: 1.58, N: 0.72. Selected FT-IR signals (ATR-Diamond) /(cm^{-1}): 3350 (br), 1629 (br), 1403 (w), 1120 (m), 1034 (m), 952 (s), 821 (w), 542 (m).

Rb-{V_{30}}: Rb-{V_{30}}: was synthesized analogously to **K-{V_{30}}**, substituting KCl for 0.138 g RbCl (1.14 mmol, 3 eq). Yield: $3x10^{-2}$ g; CHN analysis for $Rb_{27}[V_{30}P_{24}O_{140}C_{48}H_{24}K_4]N_3 \cdot 50\ H_2O$; Expected: C: 6.62, H: 1.44, N: 0.48, Found: C: 5.96, H: 1.4, N: 0.54. Selected FT-IR signals (ATR-Diamond) / (cm^{-1}): 3350 (br), 1629 (br), 1403 (w), 1120 (m), 1034 (m), 952 (s), 821 (w), 542 (s). 3367 (br), 2069 (w), 1652 br), 1122 (m), 1040 (m), 969 (s), 817 (w), 552 (s).

Cs-{V_{30}}: Cs-{V_{30}} was synthesized analogously to **K-{V_{30}}**, substituting KCl with 0.193 g CsCl (1.14 mmol, 3 eq). Yield: $2x10^{-2}$ g; CHN analysis for $Cs_{27}[V_{30}P_{24}O_{140}C_{48}H_{24}Cs_4]N_3 \cdot 50\ H_2O$; Expected: C: 5.66, H: 1.22, N: 0.41, Found: C: 5.01, H: 1.00, N: 0.38. Selected FT-IR signals (ATR-Diamond) / (cm^{-1}): 3378 (br), 2062 (w), 1649 (br), 1397 (w), 1123 (m), 1041 (m), 969 (s), 816 (w), 552 (m).

Na-{V_{30}}: Na-{V_{30}} was synthesized analogously to **K-{V_{30}}**, but without the addition of KCl. Yield: 6 mg CHN analysis for $Na_{30}[V_{30}P_{24}O_{140}C_{48}H_{24}]N_3 \cdot 70\ H_2O$; Expected: C: 8.11, H: 2.33, N: 0.59, Found: C: 7.35, H: 1.85, N: 0.69. Selected FT-IR signals (ATR-Diamond) / (cm^{-1}): 3350 (br), 1629 (br), 1403 (w), 1120 (m), 1034 (m), 952 (s), 821 (w), 542 (m).

Supplementary Note 2. Analytical and Computational Methods

<u>2.1 FT-IR Spectroscopy:</u> FT-IR spectra were recorded using a Bruker Tensor II FT-IR spectrometer with a scan rate of 16 scans min^{-1} in a spectral range of 400-4000 cm^{-1}.

<u>2.2 ESI Mass Spectrometry:</u> ESI-MS spectra were recorded on a Waters Synapt G2 HDMS instrument using water as a solvent.

<u>2.3 Powder X-ray Diffraction:</u> Samples were ground in the mother liquor and sealed in a glass capillary. The capillaries were mounted using a goniometer head on a Bruker APEX2 Duo diffractometer. Data was collected with Cu-Kα (λ=1.54 Å) radiation at 2θ values of 10° and 20° by a 360° ϕ-rotation with 10 minutes per frame at 25 °C. The data was integrated and processed using the Bruker APEX II routine XRD2-Eval subprogram.

<u>2.4 Single-Crystal X-Ray Diffraction Analysis:</u> Single-crystal X-ray diffraction analysis and refinement were performed using a Bruker APEX2 Duo diffractometer. X-ray data were measured at 100 K using an Oxford Cryosystem Cobra low temperature device and a MiTeGen micromount. Frames were integrated using the Bruker SAINT software package.[4] The data were corrected for absorption effects applying the multi-scan method, SADABS.[5] Structures were solved by Direct Methods using the ShelXT.[6] Least squares refinements were conducted using Olex2[7] and ShelXL.[8] Non-hydrogen atoms were refined anisotropically. The Paton-SQUEEZE[9] routine was employed to account for un-resolved electron density attributable to diffuse solvent molecules and disordered cations.

Crystallographic Details: The crystallographic quality values varied across the reported structures as a result of the variable diffraction intensities which are influenced by the disuse electron density of the surrounding counterions and large numbers of disordered crystallization water molecules whose positions could not be resolved. To account for the latter the SQUEEZE routine within the PLATON software package was applied. The X-ray data for **Cs-{V_{20}-*t*Bu}, Rb-** and **Cs-{V30}** were limited to sin(Θ_{max}/λ) = 0.556, 0.515 and 0.544, accounting for a low or negligible electron density at higher Θ angles. The structural disorder within the {V_5O_9} units and disorder due to superimposed, structural {V_{30}} isomers, affect the crystal structure refinement and influence the V-O bond precisions and the associated Bond-Valence Sum Analysis.

Although almost identical in their core structures, the crystal structures of **M-{V_{20}}**, **M= Cs, Rb, K** were solved in three different space groups. **K-{V_{20}}** was solved in the lower symmetry monoclinic space group *C2/m* whereas **Rb-{V_{20}}** and **Cs-{V_{20}}** were solved in the higher symmetry tetragonal space groups *I4/mmm* and *P4/mnc*. The change from the body-centred cell of *I4/mmm* for **Rb-{V_{20}}** to the primitive tetragonal cell of *P4/mnc* for **Cs-{V_{20}}** is associated with a slight rotation of the clusters in respect to neighbouring clusters located on the edges of the unit cell (as shown in Supplementary Figure 3.). In **Cs-{V_{20}}**, the clusters are rotated *ca*. 6° against each other, which reduces the symmetry of the crystal structure. The difference is associated with intermolecular π-π interactions. *P4/mnc* is a 'klassengleiche' k-subgroup to *I4/mmm* with index 2. This means that *P4/mnc* and *I4/mmm* share the same point group but not all translations. In this case the slight rotation of the central {V_{20}} cluster leads to the removal of all body-centred translations of *I4/mmm*, resulting in *P4/mnc*. The **{V_{20}-NH_2}** and **{V_{20}-Py}** derivatives also crystallize in *P4/mnc* and *I4/mmm*, respectively. The introduction of *tert*-butyl groups lowers the symmetry and **Rb-{V_{20}-*t*Bu}** and **Cs-{V_{20}-*t*Bu}** were solved in the monoclinic *C2/m* and triclinic P-1 space groups. All **M-{V_{20}}** structures are composed of four {V_5O_9}$_o$ units in which {O_4V^{IV}=O} moieties are rotated in respect to {V_5O_9}$_c$ units. Within each {V_5O_9}$_o$ unit two different {O_4V^{IV}=O} vanadyl groups can be inverted. For all **M-{V_{20}}** structures, this was modelled as disordered

in a 50:50 occupancy. The **K-{V_{30}}** structure was solved in the triclinic space group *P*-1. The structure was refined as a disordered structure consisting of a mixture of two superimposed, isomeric structures that are occupying the same position within the unit cell. The isomers **K-{V_{30}}$_{2c/4o}$** and **K-{V_{30}}$_{4c/2o}$** which differ by the orientation of the {V_5O_9} units, as well as the presence of the cubic alkali metal assembly in the centre of the cage. Both structures were modelled to have an occupancy of 50 %. In addition, the two different possible positions of the inverted {O_4V^{IV}=O} moieties in the {V_5O_9}$_o$ units give rise to the disorder within the disordered parts. Considering the nature of the positional disorder, the observed cation-π interactions in **K-{V_{30}}** may conceptionally be associated with either isomer, **K-{V_{30}}$_{2c/4o}$** and/or **K-{V_{30}}$_{4c/2o}$**. The crystal structure of **Na-{V_{30}}** clarifies this ambiguity. The structure was refined in the triclinic space group *P*-1 as singular **Na-{V_{30}}$_{4c/2o}$** isomer with minor disorder of some V centres, showing no signs of cation-π interactions. While it is possible that disordered sodium ions may be present within the cavity of **Na-{V_{30}}$_{4c/2o}$**, none of them located in the vicinity of phenyl rings as in the **K-{V_{30}},** confirming the association of the cation-π interactions within {V_{20}} ring moieties in **K-{V_{30}}$_{2c/4o}$.** In the **{Na-V_{30}}** structure the inversion/flipping of the V=O moieties in the {V_5O_9}$_o$ units only occurs at very low propensity. Weakly diffracting crystals for **Rb-{V_{30}}** were obtained. Although the crystals may be twinned and X-ray data is of low quality, refinement in the monoclinic *C*2/*c* space group demonstrates the structural resemblance to **K-{V_{30}}**. Crystals of **Cs-{V_{30}}** were small and relatively weakly diffracting giving rise to a lower resolution structure that was refined in the triclinic space group *P*-1. The X-ray data allowed the location of four Cs^+ ions in the inner cage structure and established the atom connectivity of a **Cs-{V_{30}}$_{3o/3c}$** cage structure.

<u>2.5 SQUID Measurements:</u> The magnetic susceptibility measurements were obtained using a MPMS-XL Quantum Design SQUID magnetometer.

<u>2.6 UV-VIS Spectroscopy:</u> UV-VIS spectra were recorded on a Perkin Elmer Lambda 35 spectrometer using a quartz cuvettes with a path length of 10mm at room temperature. All spectra were analyzed using the UVWinlab software.

<u>2.7 Elemental Analysis:</u> Microanalytical analysis was undertaken using a Exeter Analytical CE 440 instrument at the Microanalysis Laboratory of the School of Chemistry and Chemical Biology in the University College Dublin.

<u>2.9 Computational Analysis:</u> DFT calculations, were carried out using the Gaussian09 package[10] implementing the hybrid DFT functional PBE0[11] in conjunction with basis sets, 6-31G(p,d,2d,)[12] to model H-, C-, N- O and P-atoms, respectively and SDDALL[13], providing effective core potentials for the V atoms. The choice of functionals and basis sets was informed by our previous experience and literature evidence demonstrating their suitability to accurately compute structural and electronic properties of related first-row transition metal complexes including vanadate species.[14-17]

In the applied model the phosphonate ligands were replaced by methylphosphonate ligands for computational easement. The structural input coordinates for the {V_5O_9}$_c$ arrangement were consistent to those of conventional *'closo'* cages.[18-S0] The initial coordinates for {V_5O_9}$_o$ derive from **Cs-{V_{20}}**. A calculation using {V_5O_9}$_c$ includes a templating H_2O molecule located at the focal point of the half-capsule. The location of the H_2O molecule was constrained at a distance of 3.1 Å from the central V^V atom. The default convergence criteria and a larger integration grid containing 225 radial shells with each shell containing 974 angular points were used. For each system, only the ferromagnetic configuration was modelled. Frequency calculations were carried out to determine the free energy of the different systems.

2.10 Bond Valence Sum Analysis: The applied model drives form the Bond Valence Model as described in the literature.[21-23] It follows Pauling's electrostatic valence concept,[S24] whereby the sum of bond valences S_{ij} around an ion i over all neighboring atoms j, is equal to the formal oxidation state V_i.

$$V_i = \sum_j S_{ij}$$

$$S_{ij} = \exp\left(\frac{R_0 - R_{ij}}{B}\right)$$

S_{ij} relates to the bond strength and correlates inversely with bond length, providing a measure of the electrostatic flux between a cation and an anion. R_0 and B are tabulated parameters and R_{ij} is the interatomic distance between atoms i and j. The parameters R_0 and B (0.37) were taken from:https://www.iucr.org/resources/data/datasets/bond-valence-parameters (2016 version).

Supplementary Figures

A

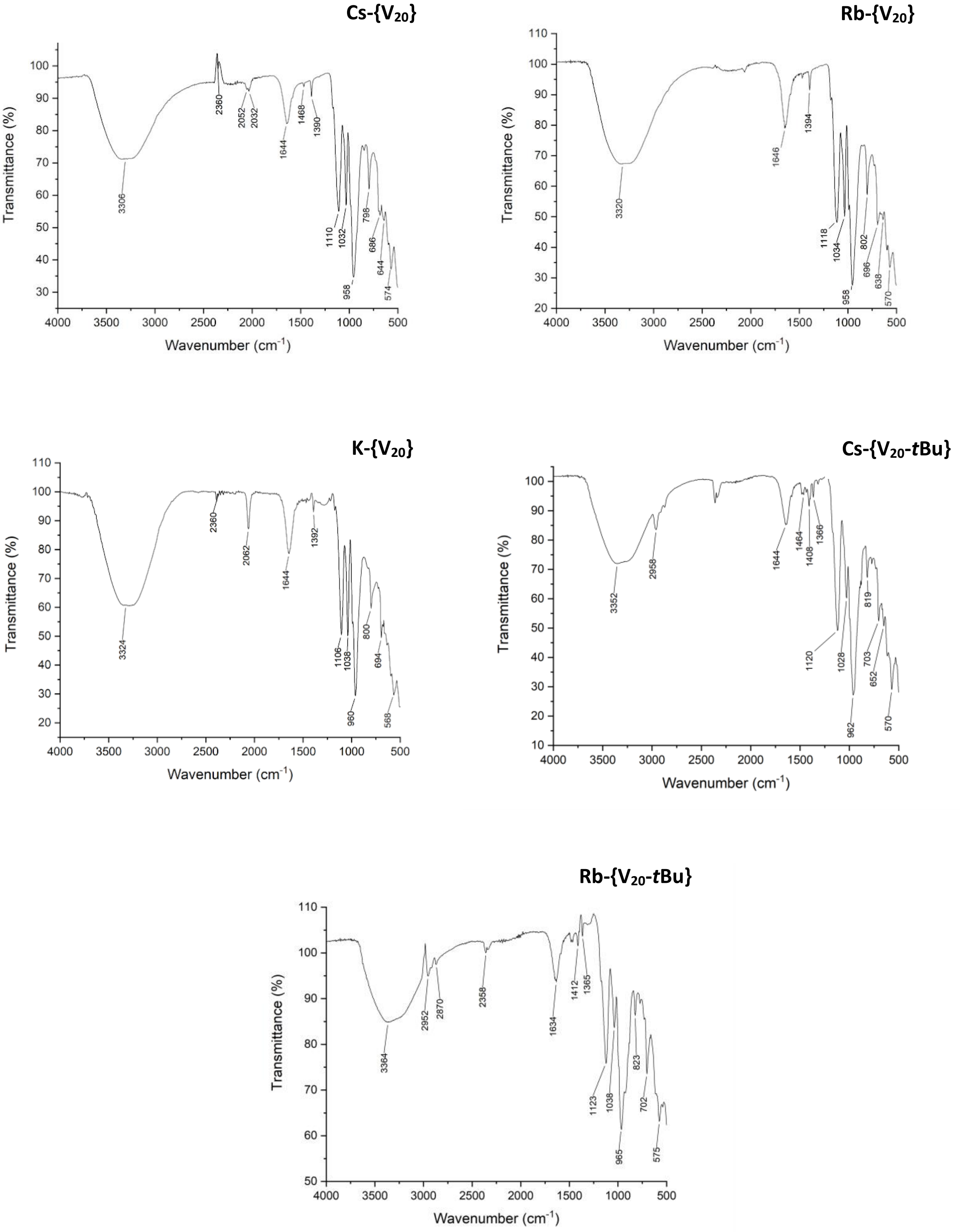

B

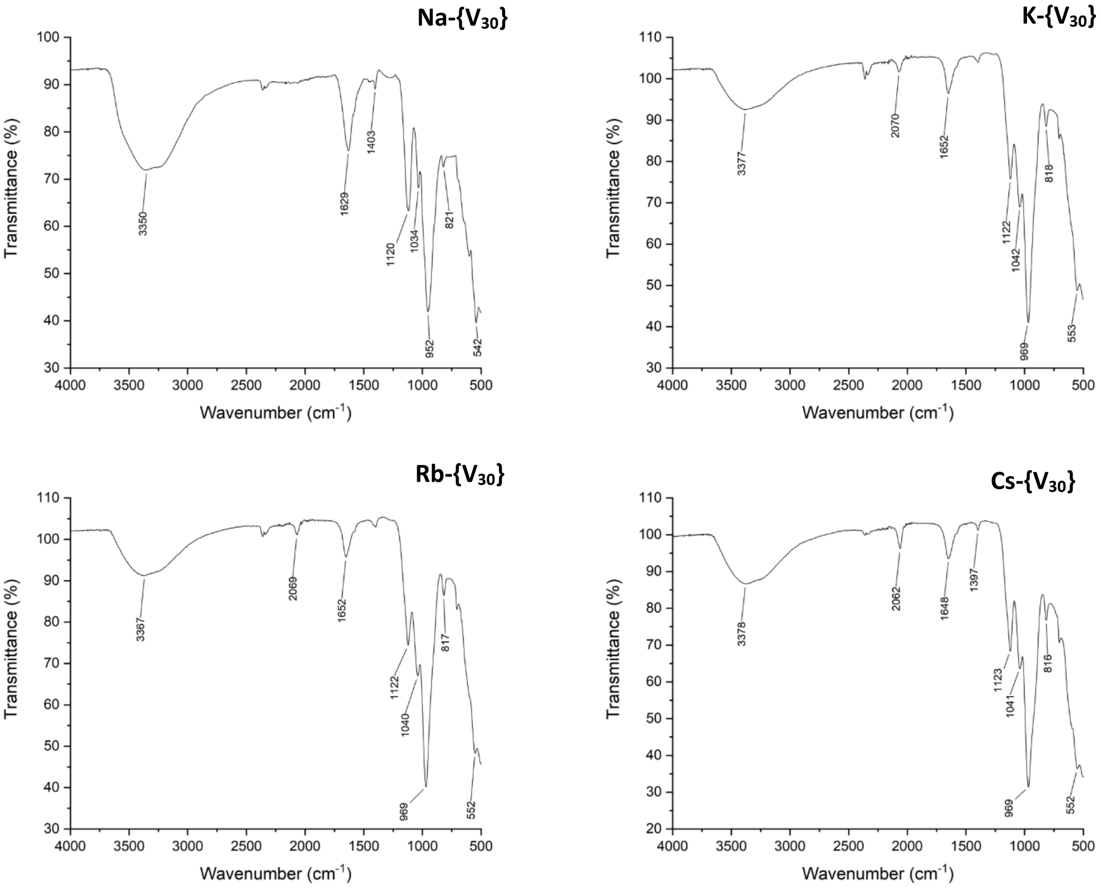

Supplementary Figure 1. FT-IR spectra of various vanadate $\{V_{20}\}$ (**A**) and $\{V_{30}\}$ (**B**) cages. Note: The signals at *ca.* 1110-1125 cm^{-1} and *ca.* 1040 cm^{-1} can be attributed to the {P-O} vibrations of the coordinating phosphonate ligands while the broad signals at *ca.* 960-970 cm^{-1} stem from vibrations of the $\{V^{V}=O\}$ and $\{V^{IV}=O\}$ moieties.[25-27] The broadness of the latter signal and observed shoulders are consistent with the mixed-valent nature of the compounds. [27-29] The broad signals around 3370 cm^{-1} are due to O-H vibrations of H-bonded water molecules and water molecules that bind to the counterions. The bending {H-O-} vibrations of these constitutional H_2O molecules appear at *ca.* 1640-1660 cm^{-1}. {V–O–V} bending vibrations are expected to contribute to bands <650cm^{-1}. [25-29]

A

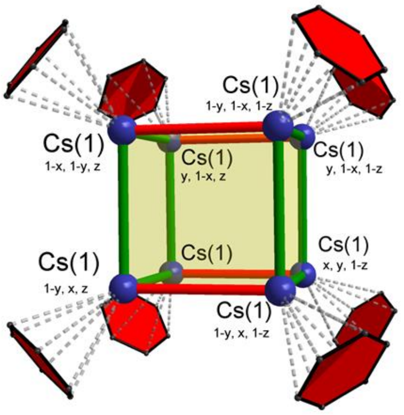

Cs-{V_{20}}

5.0059(2) Å

4.5138(1) Å

Distance to central Na^+: **4.0561(1) Å**

Space group: ***P4/mnc***

B

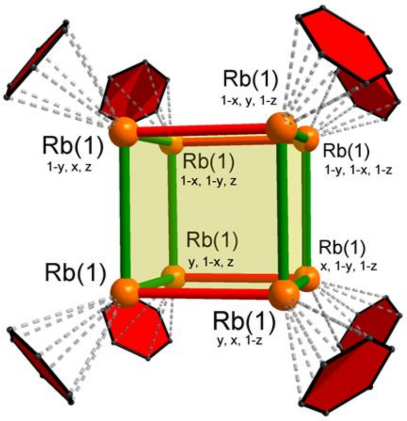

Rb-{V_{20}}

5.0332(2) Å

4.5741(1) Å

Distance to central Na^+: **4.0981(1)** Å

Space group: ***I4/mmm***

C

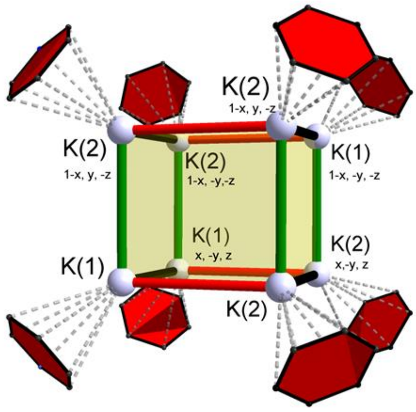

K-{V_{20}}

5.0198(2) Å

4.6320(2) Å

4.6365(2) Å

Distances to central K^+:

K(1) 4.1221(2) Å; K(2) 4.1231(2) Å

Space group: ***C2/m***

D

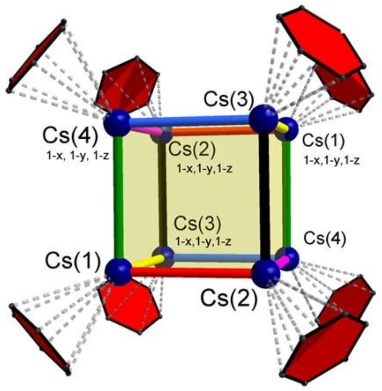

Cs-{V_{20}-*t*Bu}

- 4.9242(2) Å (blue)
- 4.8891(2) Å (red)
- 4.6049(2) Å (black)
- 4.6274(2) Å (green)
- 4.5521(3) Å (magenta)
- 4.5484(3) Å (yellow)

Distances to central Na^+:
Cs(1) 4.0600(3) Å; Cs(2) 4.0658(2) Å; Cs(3) 4.0461(2) Å; Cs(4) 4.0869(2) Å

Space group: ***C2/m***

E

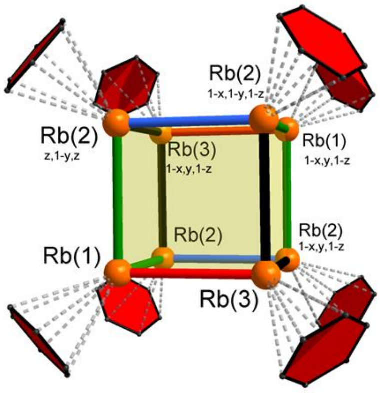

Rb-{V_{20}-*t*Bu}

- 5.0680(5) Å (blue)
- 4.9595(5) Å (red)
- 4.5120(2) Å (black)
- 4.5306(2) Å (green)

Distances to central Na^+:
Rb(1) 4.0781(2) Å; Rb(2) 4.0681(2) Å; Rb(3)4.0360(2) Å

Space group: ***P*-1**

F

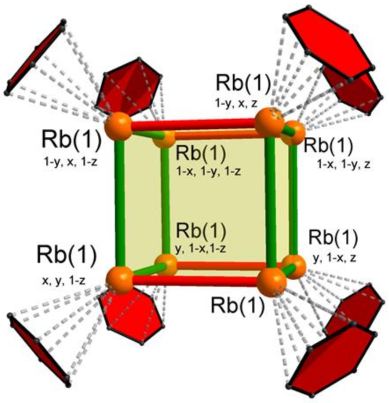

Rb-{V_{20}-NH_2}

- 4.9270(2)) Å (red)
- 4.5806(2) Å (green)

Distance to central Na^+: **4.0694(1) Å**

Space group: ***P4/mnc***

G

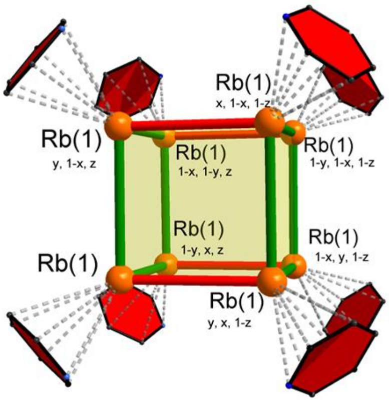

Rb-{V_{20}-Py}

- 4.7365(2) Å (red)
- 4.5076(2) Å (green)

Distance to central Na^+: **3.9709(1) Å**

Space group: ***I4/mmm***

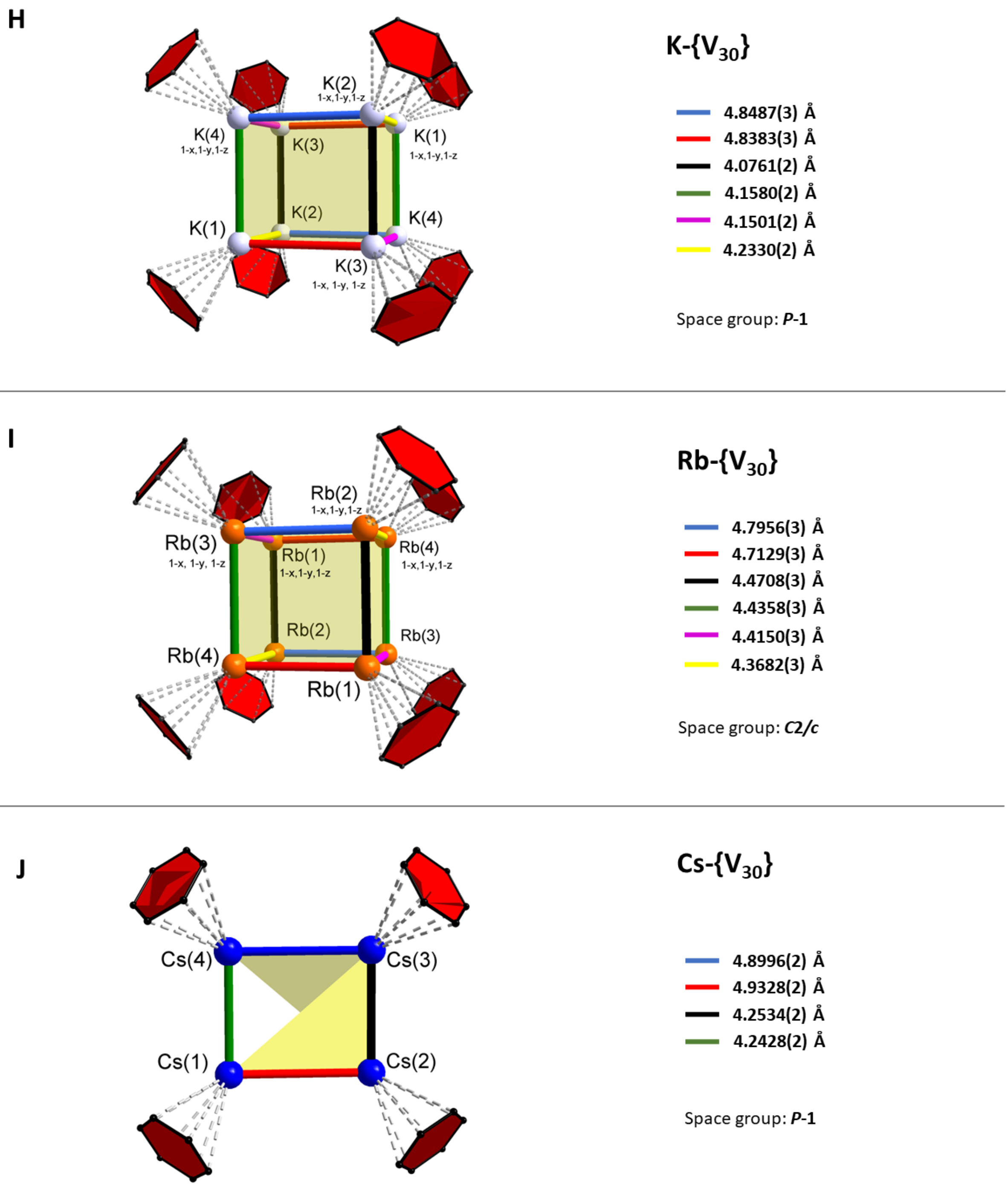

Supplementary Figure 2. Alkali metal distances within the cage structures. **A-C Cs-{V_{20}}**, **Rb-{V_{20}}** and **K-{V_{20}}**, respectively; **D-G Cs-{V_{20}-*t*Bu}**, **Rb-{V_{20}-*t*Bu}**, **Rb-{V_{20}-NH_2}** and **Rb-{V_{20}-Py}**, respectively; **H-J K-{V_{30}}**, **Rb-{V_{30}}** and **Cs-{V_{30}}**, respectively.

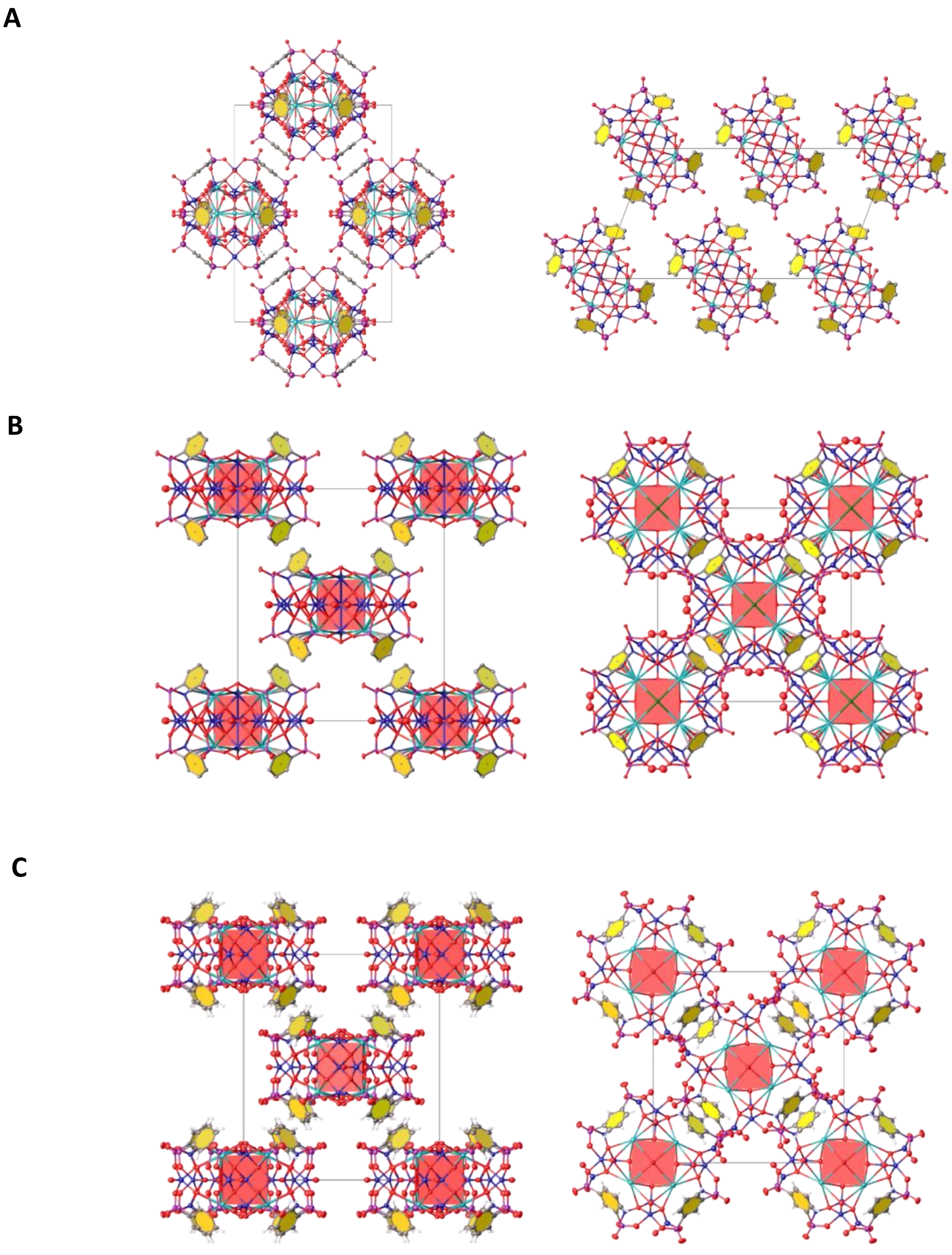

Supplementary Figure 3. A Packing diagram of **K-{V_{20}}** with view in the direction of the crystallographic *c*-axis (left) and *b*-axis (right); **B** Packing of **Rb-{V_{20}}** with view in the direction of the crystallographic *a*-axis (*left*) and *c*-axis (*right*). **C** Packing of **Cs-{V_{20}}** with view in the direction of the crystallographic *a*-axis (*left*) and *c*-axis (*right*).

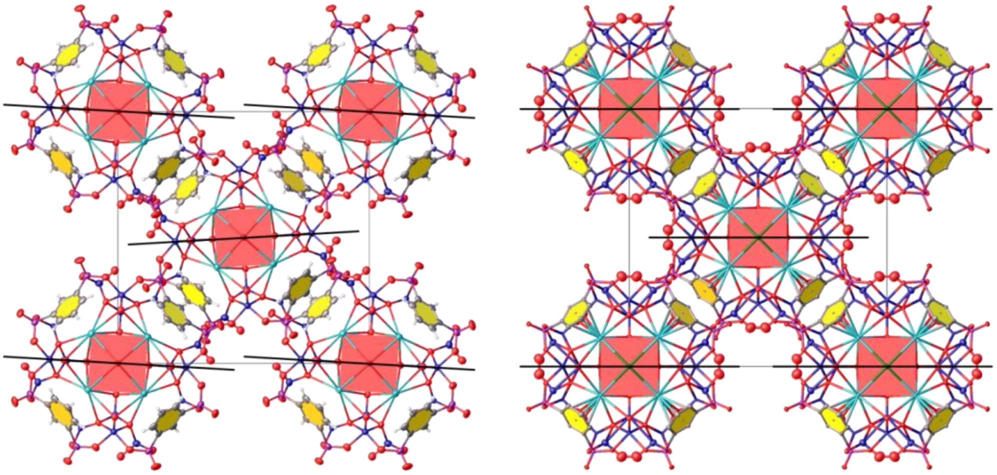

Supplementary Figure 4. Comparison of the packing between **Cs-{V_{20}}** (*left*) and **Rb-{V_{20}}** (*right*) with view in the [100]-direction. The lines are drawn through the centres of the oxo-clusters and indicate their relative orientations.

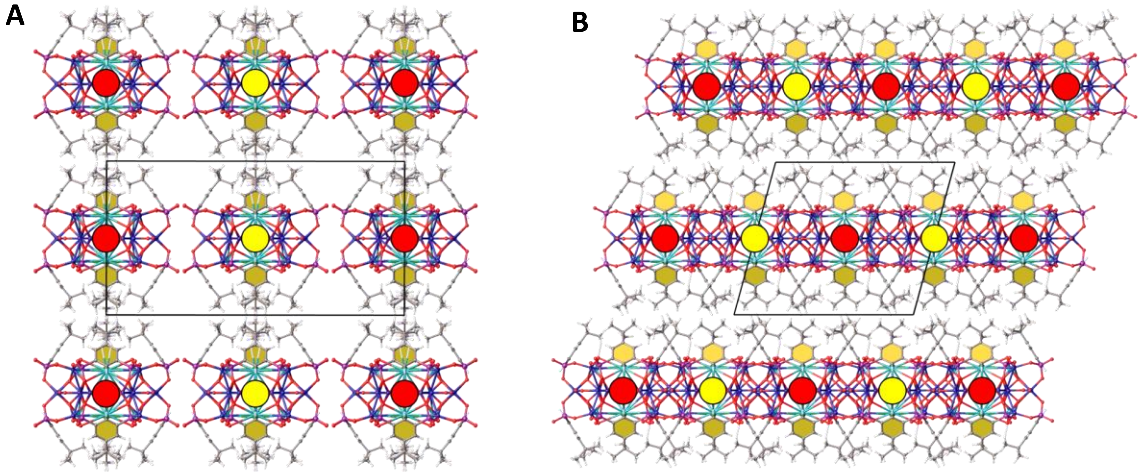

Supplementary Figure 5. Packing diagram of **Rb-{V_{20}-*t*Bu}**. **A** View in the [100]-direction; **B** View in the [010]-direction. The red and yellow circles visualise different height positions in the viewing direction.

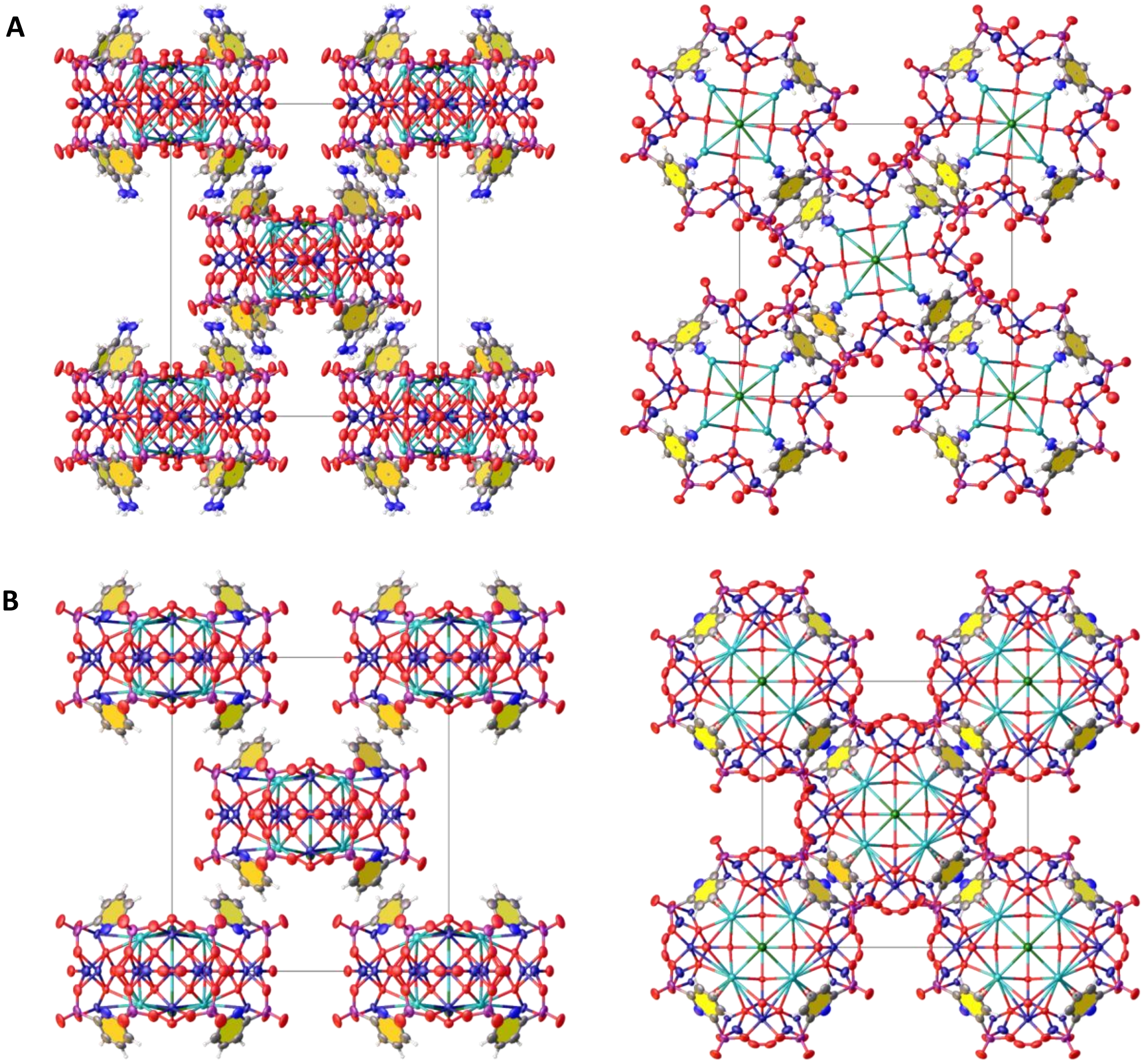

Supplementary Figure 6. A Packing diagrams for **Rb-{V_{20}-NH_2}** in the [100]-direction (*left*) and [001]-direction (*right*); **B** Packing diagram of **Rb-{V_{20}-Py}** in the [100] (*left*) and [001] directions (*right*).

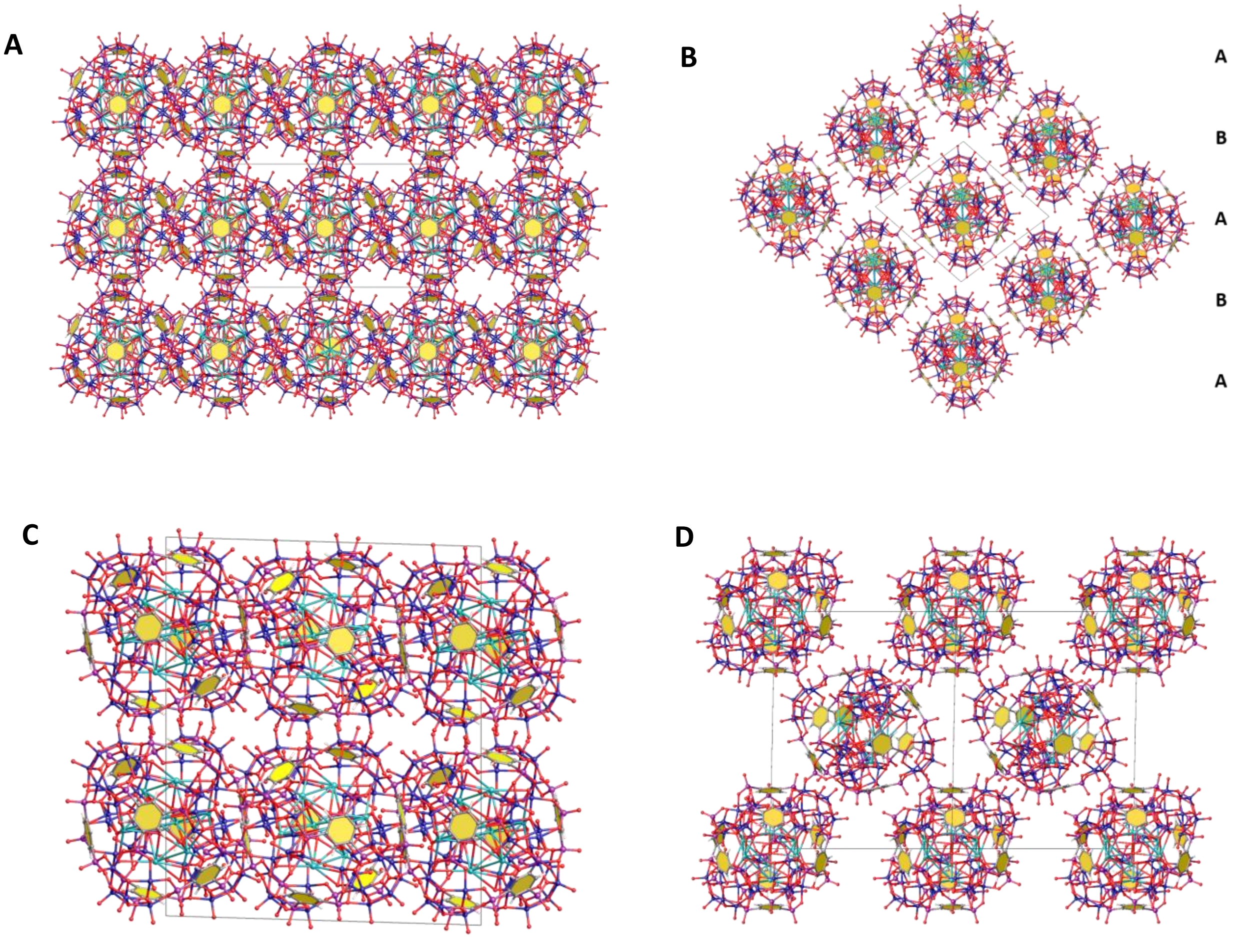

Supplementary Figure 7. A Packing of **K-{V_{30}}** with view in the [110]-direction. **B** Packing of **K-{V_{30}}** with view in the direction of the crystallographic *c*-axis. The clusters stack in [110] direction in *ABAB* fashion; **C** Packing of **Rb-{V_{30}}** with view down the crystallographic *b*-axis; **D** Packing of **Rb-{V_{30}}** with view in the crystallographic [110] direction.

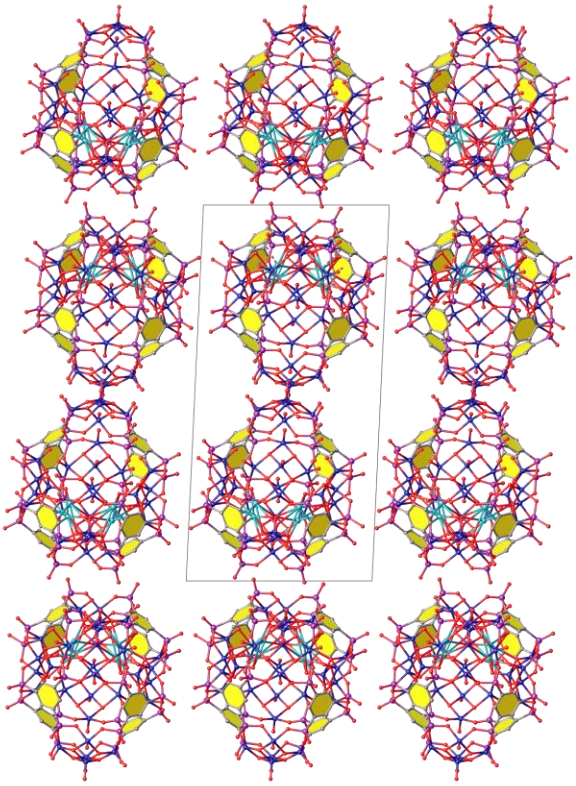

Supplementary Figure 8. Packing of **Cs-{V_{30}}** with view in the [010] direction.

5

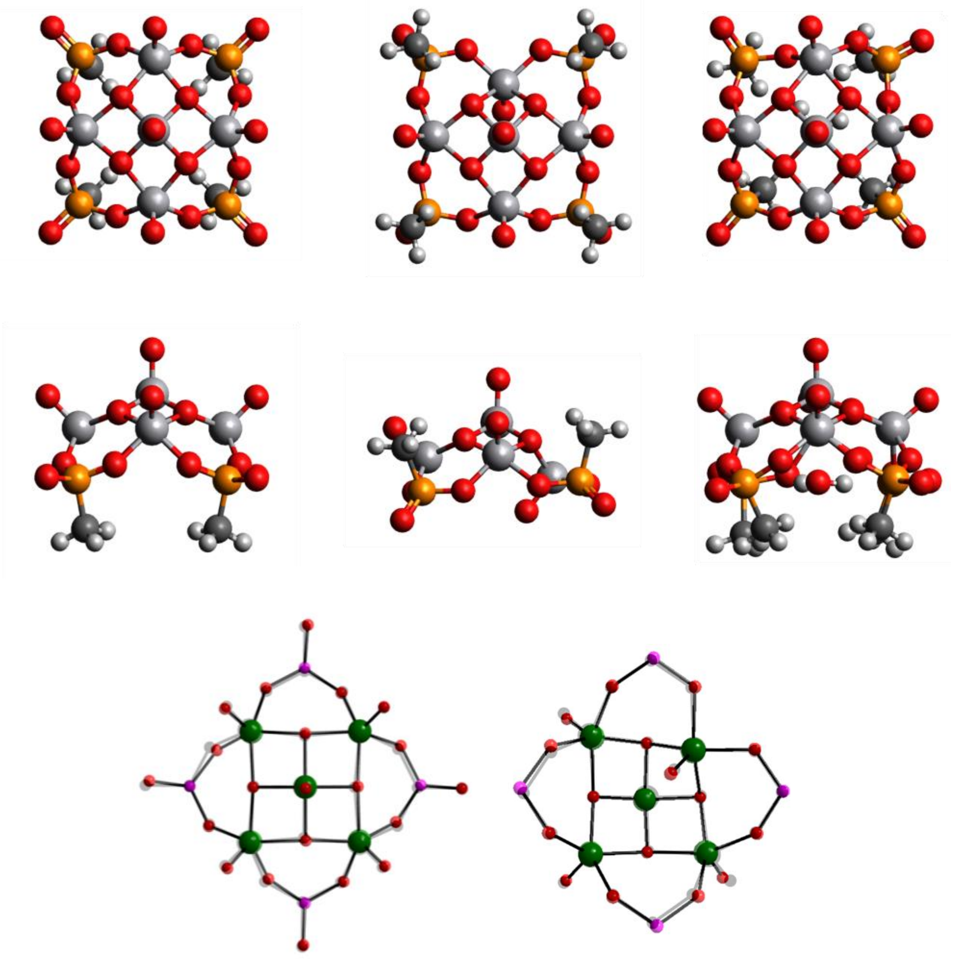

Model	Free Energy / Hartrees	Stabilisation / (kJ/mol)
**Water**	-76.331661	
**$\{V_5O_9\}$**	-3461.41635	
**$\{V_5O_9\}$ inverted**	-3461.405869	+ 27.5 vs. $\{V_5O_9\}$<br>+ 63.8 vs. $\{V_5O_9(H_2O)\}$
**$\{V_5O_9(H_2O)\}$**	-3537.761812	-36.2 vs. $\{V_5O_9\}$+H_2O

Supplementary Figure 9. *Top*: DFT energy minimisations for $\{V_5O_9\}_c$, $\{V_5O_9\}_o$ and $\{V_5O_9\}_c$ + H_2O. Structural data for $\{V_5O_9\}_c$ is consistent with reported $\{V_{10}\}$ molecular capsules.[18-20] *Middle*: Overlay of experimental structure (coloured) and calculated structure (black shadow) for $\{V_5O_9\}_c$ and $\{V_5O_9\}_o$ (experimental structural data taken from the literature[20] and from **K-$\{V_{20}\}$**. Coordinate files of calculated structure are available as xyz-files). *Bottom*: Relative Energy values associated with the DFT minimisations.

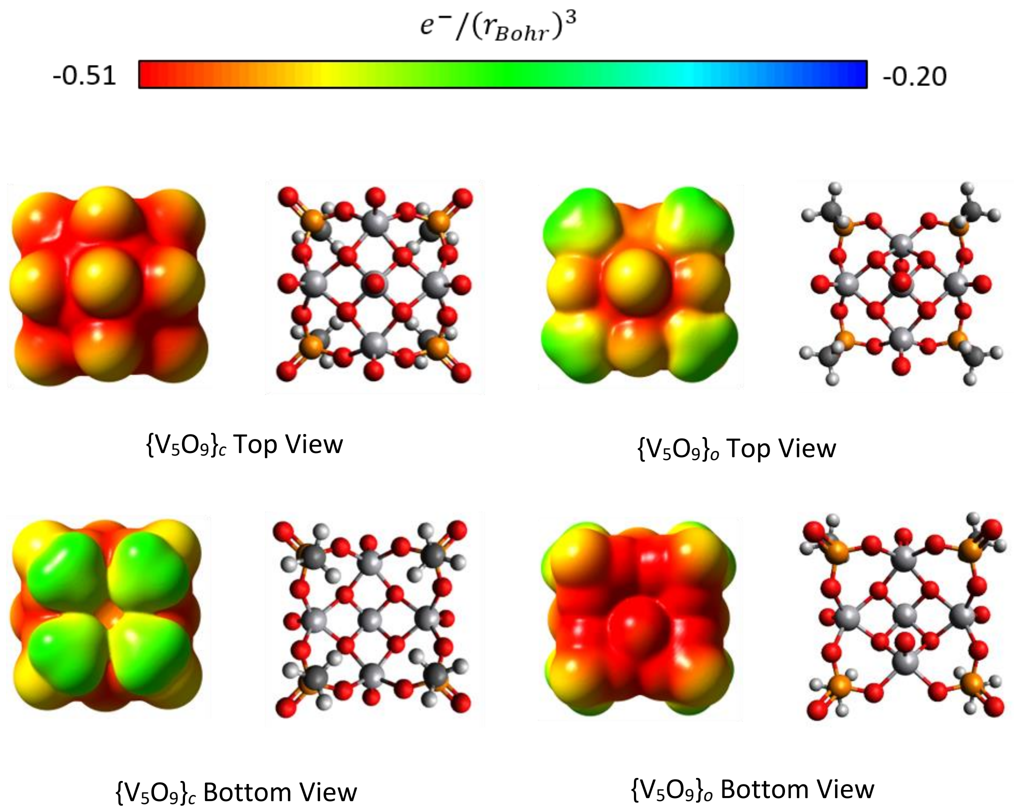

Supplementary Figure 10. Molecular electrostatic potential maps for the $\{V_5O_9\}_c$ and $\{V_5O_9\}_c$ half-capsules. Molecular electrostatic potential at $\boldsymbol{\rho(r) = 0.02\ \mathrm{a.u.}}$ from -0.51 to -0.2 $\boldsymbol{e^-/(r_{Bohr})^3}$ derived from DFT calculations using structural models of $\{V_5O_9\}$ units in which the phenyl rings of the organophosphonate ligands were terminated by methyl groups to reduce the computation time. Structural data for $\{V_5O_9\}_c$ is consistent with conventional $\{V_{10}\}$ molecular capsules.[18-20] Areas of higher electron density are coloured red, whereas the areas of lower electron density are coloured green to blue.

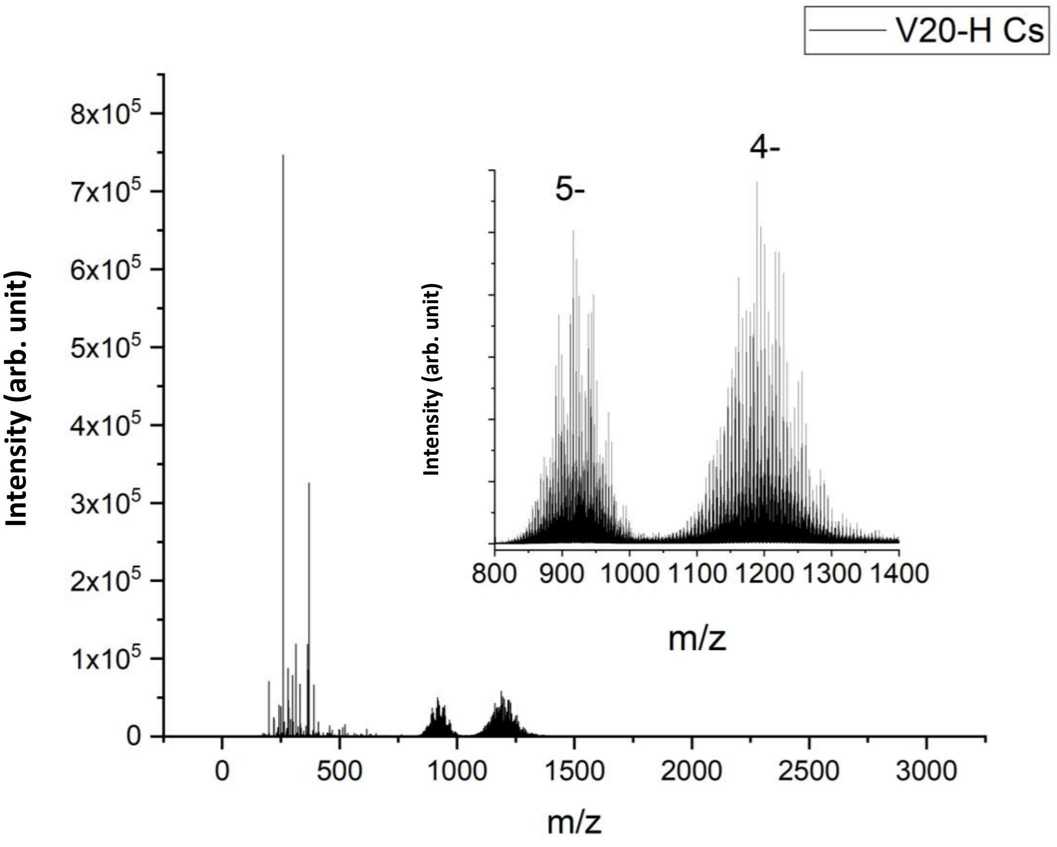

Supplementary Figure 11. ESI-MS of **Cs-{V_{20}}** in water. Two broad envelopes corresponding to the -5- and -4 charged species are detectable along with fragments in the lower mass region.

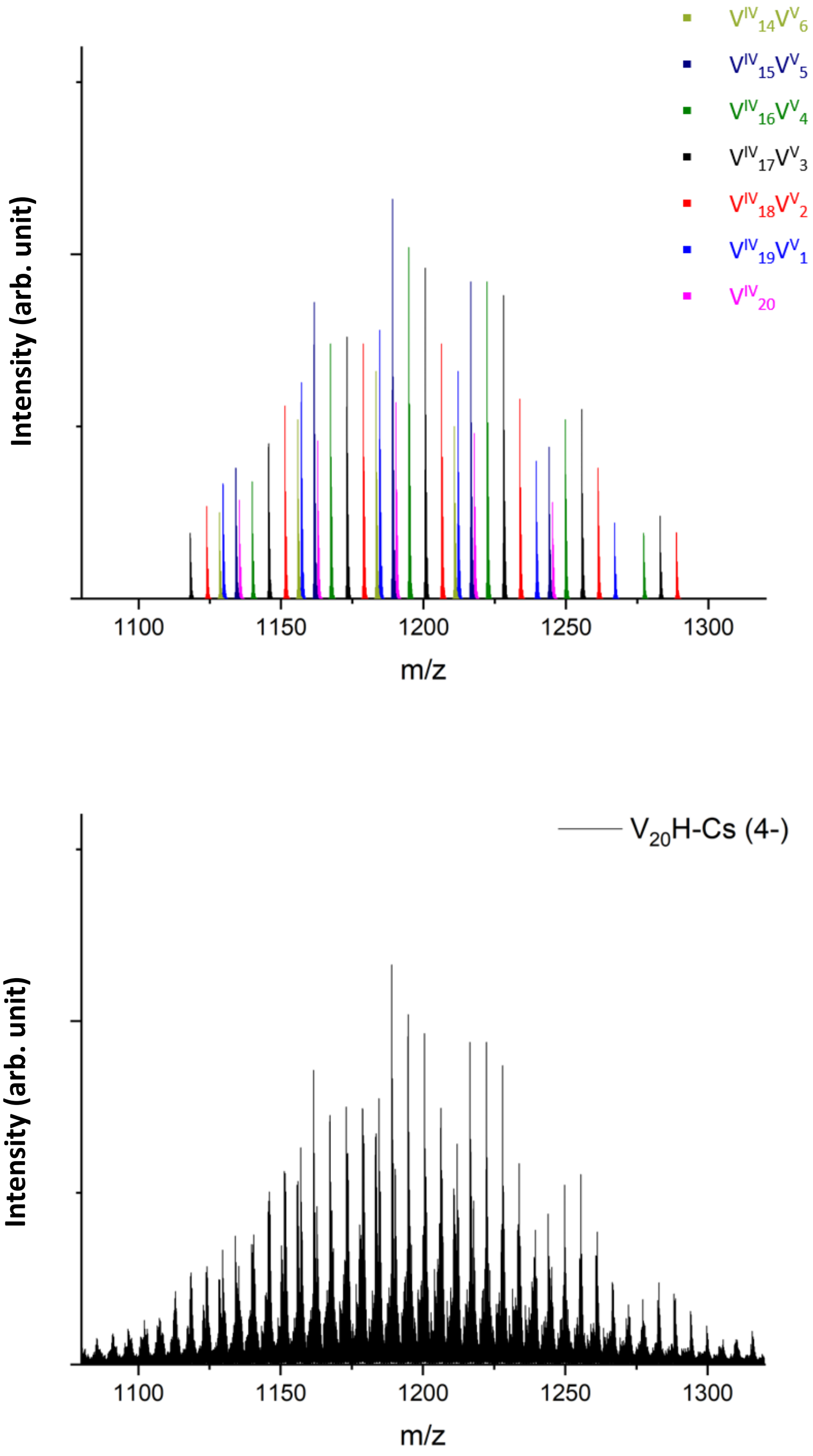

Supplementary Figure 12. The experimental envelope arising for -4 charged species in the ESI-MS spectrum of **Cs-{V_{20}}** (*bottom*) and the modelled envelope (*top*). Different colours represent species with various oxidation states.

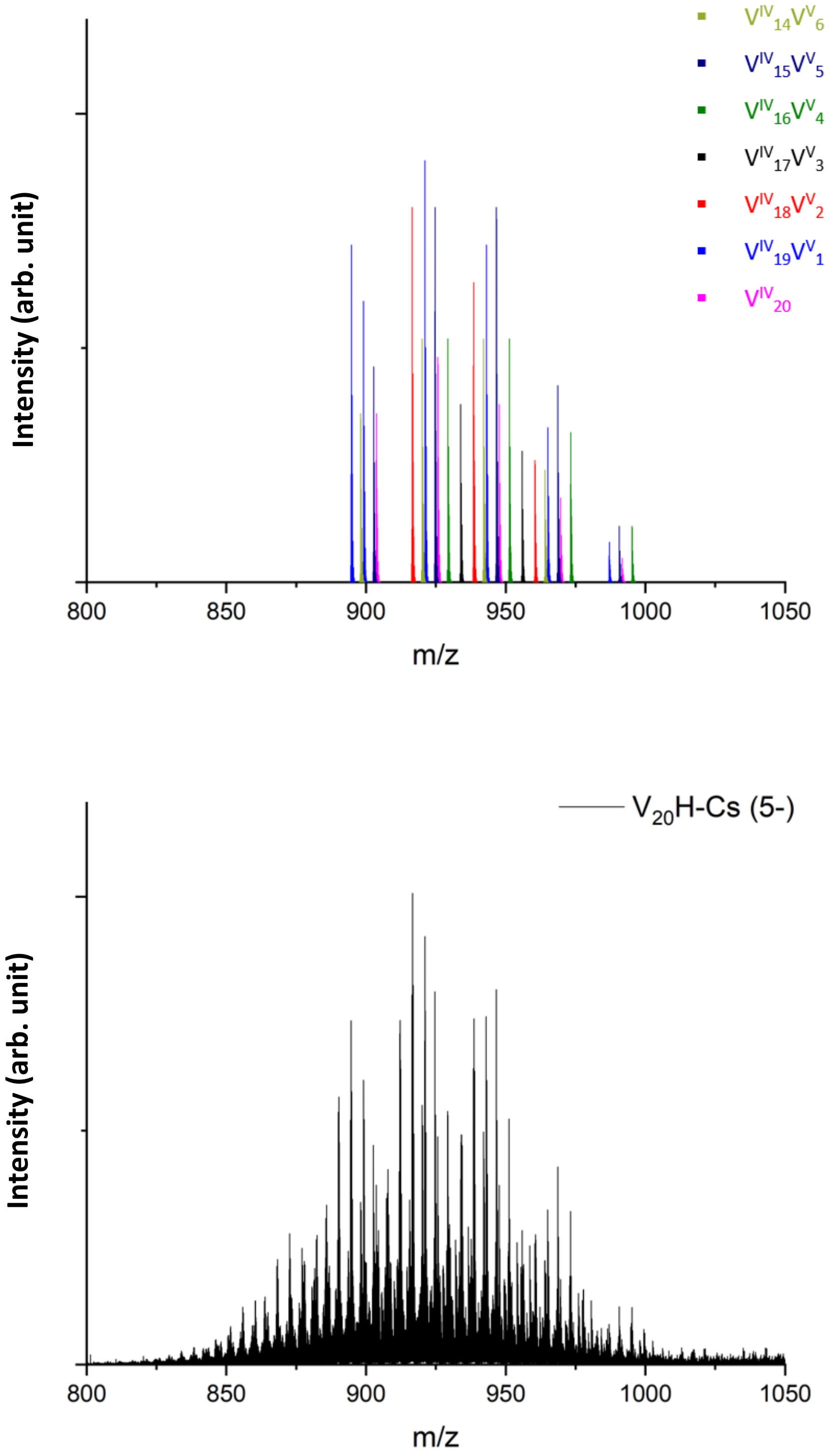

Supplementary Figure 13. The experimental envelope arising from -5 charged species in the ESI-MS spectrum of **Cs-{V_{20}}** (*bottom*) and the modelled envelope (*top*). Different colours represent species with various oxidation states.

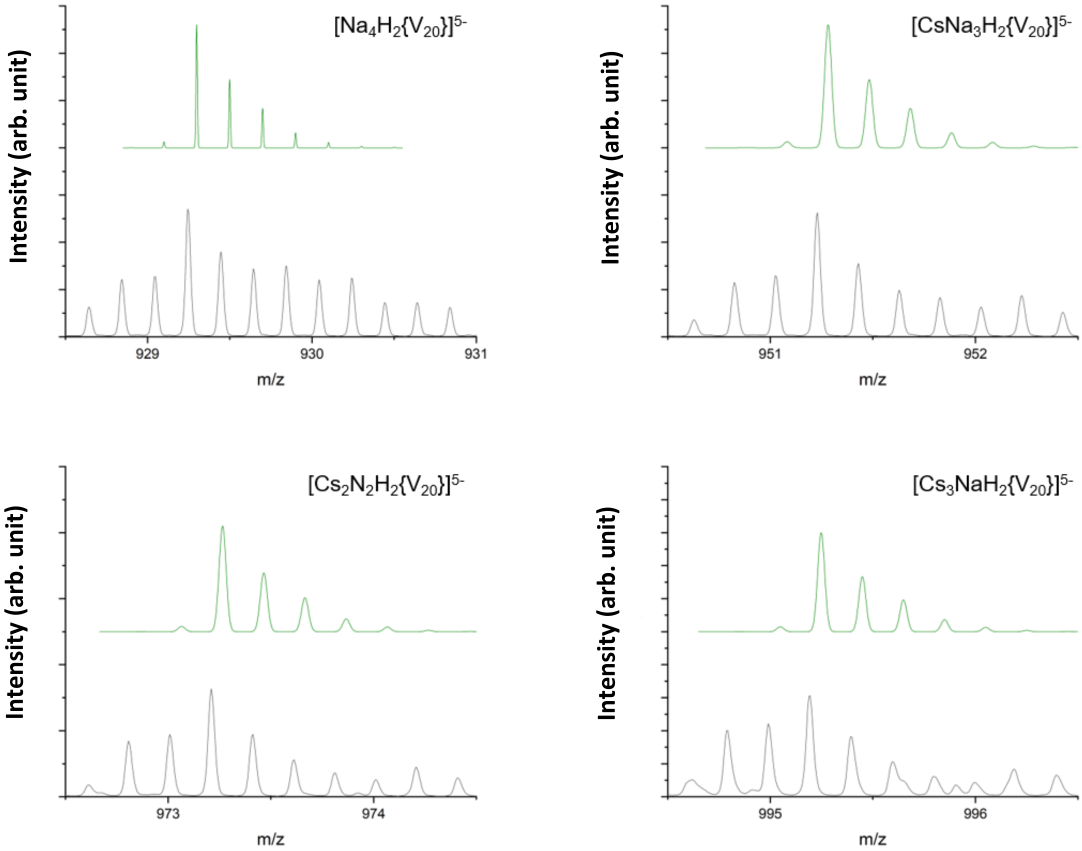

Supplementary Figure 14. Simulated (*top*) and experimental (*bottom*) isotopic patterns for $\{V^{IV}_{16}V^{V}_{4}\}$ species in the envelope arising from -5 charged species in the ESI-MS spectrum of **Cs-{V$_{20}$}**.

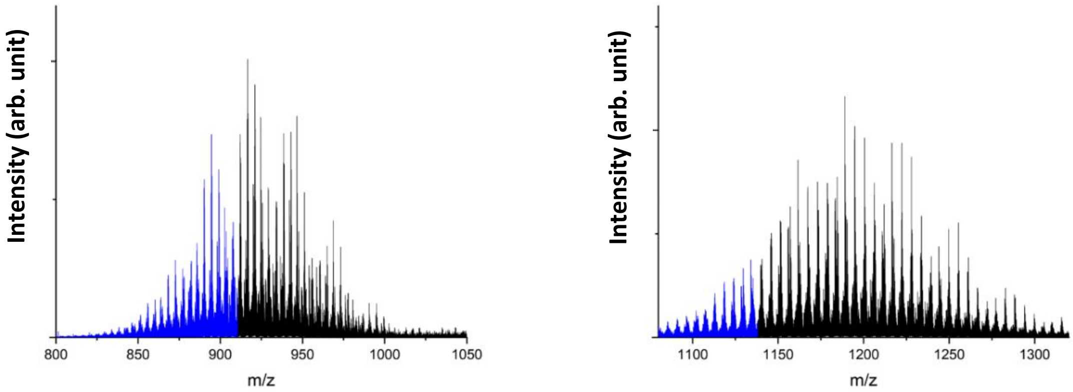

Supplementary Figure 15. Envelopes arising from -5 and -4 species of **Cs-{V$_{20}$}**. The blue parts of the spectra highlight signals of species which have lost Cs^+ ions from the central cubic alkali metal moiety of the cage structure.

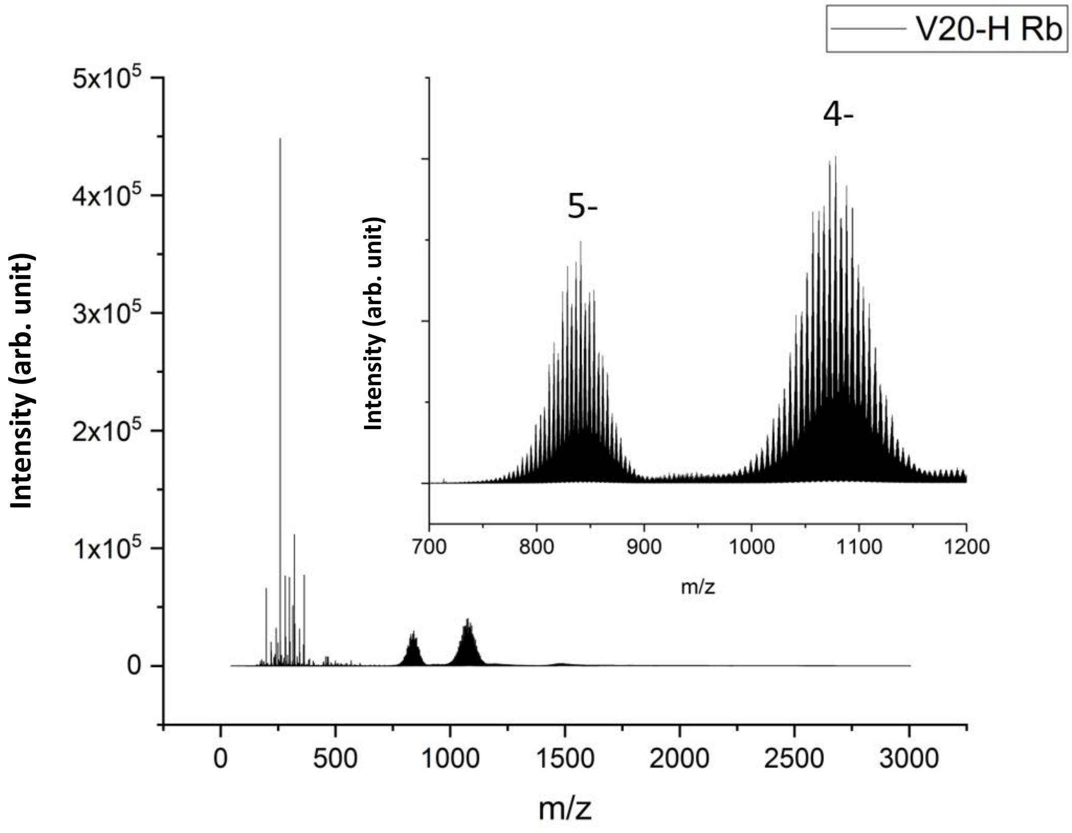

Supplementary Figure 16. ESI-MS spectrum of **Rb-{V_{20}}** in water. Two broad envelopes corresponding to the -5 and -4 charged species are detectable along with fragments in the lower mass region.

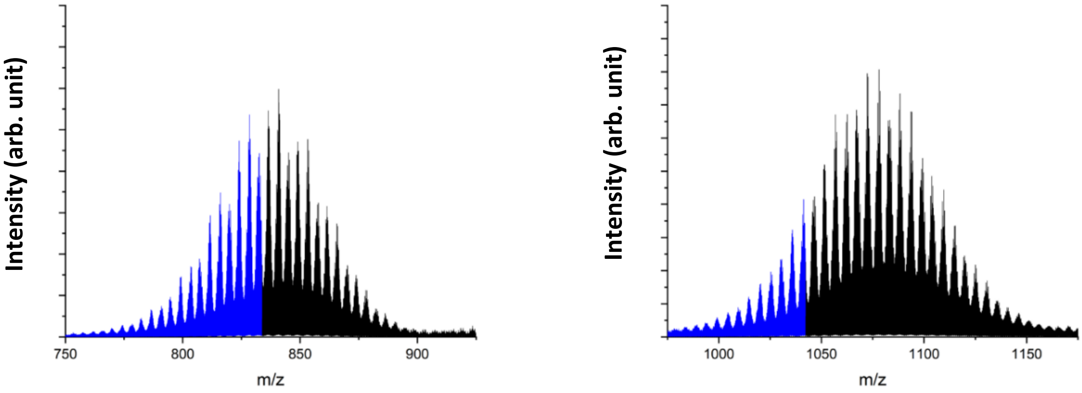

Supplementary Figure 17. Envelopes arising from -5 and -4 charged species of **Rb-{V_{20}}**. The blue parts of the spectra highlight signals of clusters which have lost Rb^+ ions from the central cubic alkali metal moiety of their cage structure.

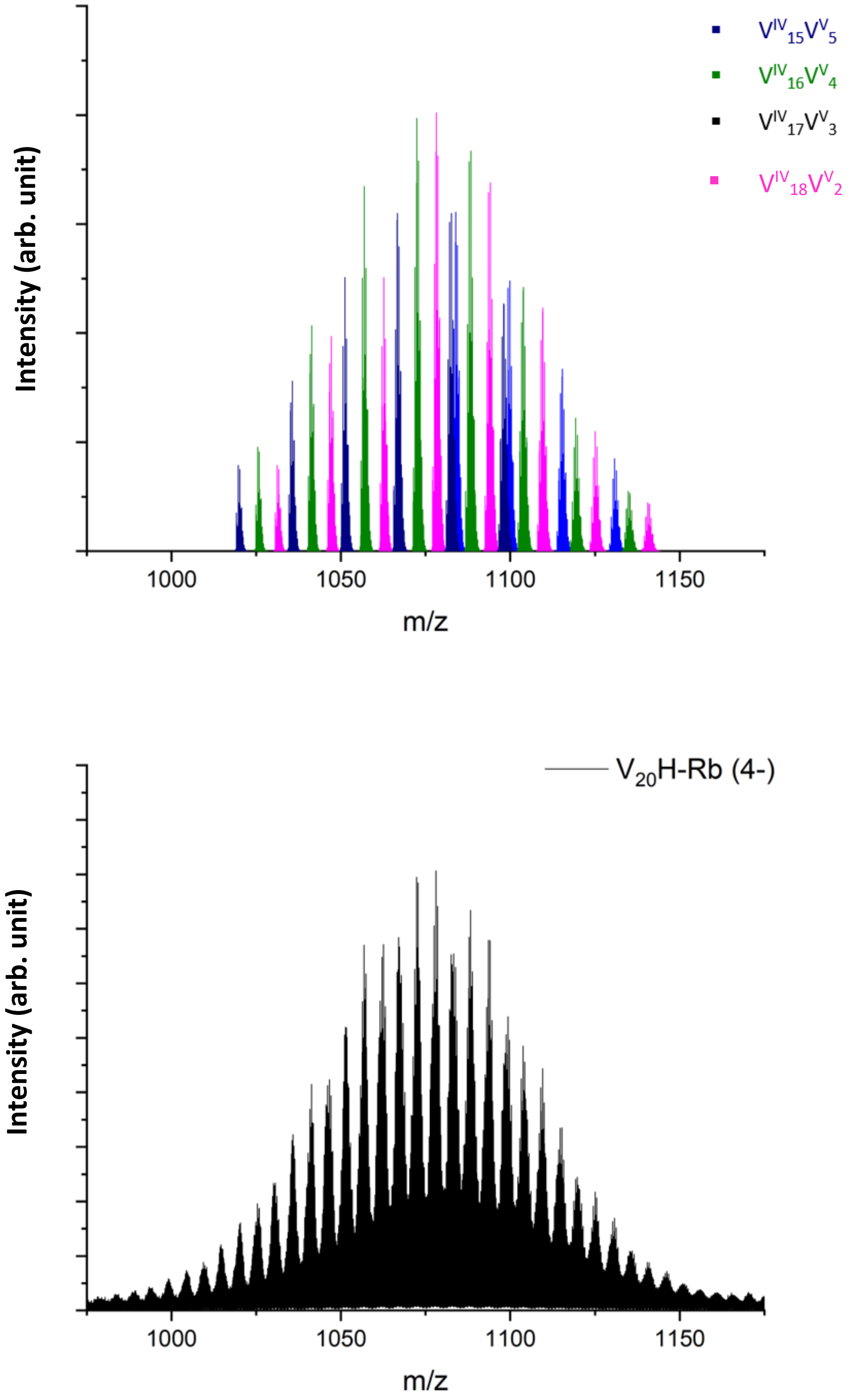

Supplementary Figure 18. The experimental envelope arising from -4 charged species in the ESI-MS spectrum of **Rb-{V_{20}}** (*bottom*) and the modelled envelope (*top*). Different colours represent species with various oxidation states.

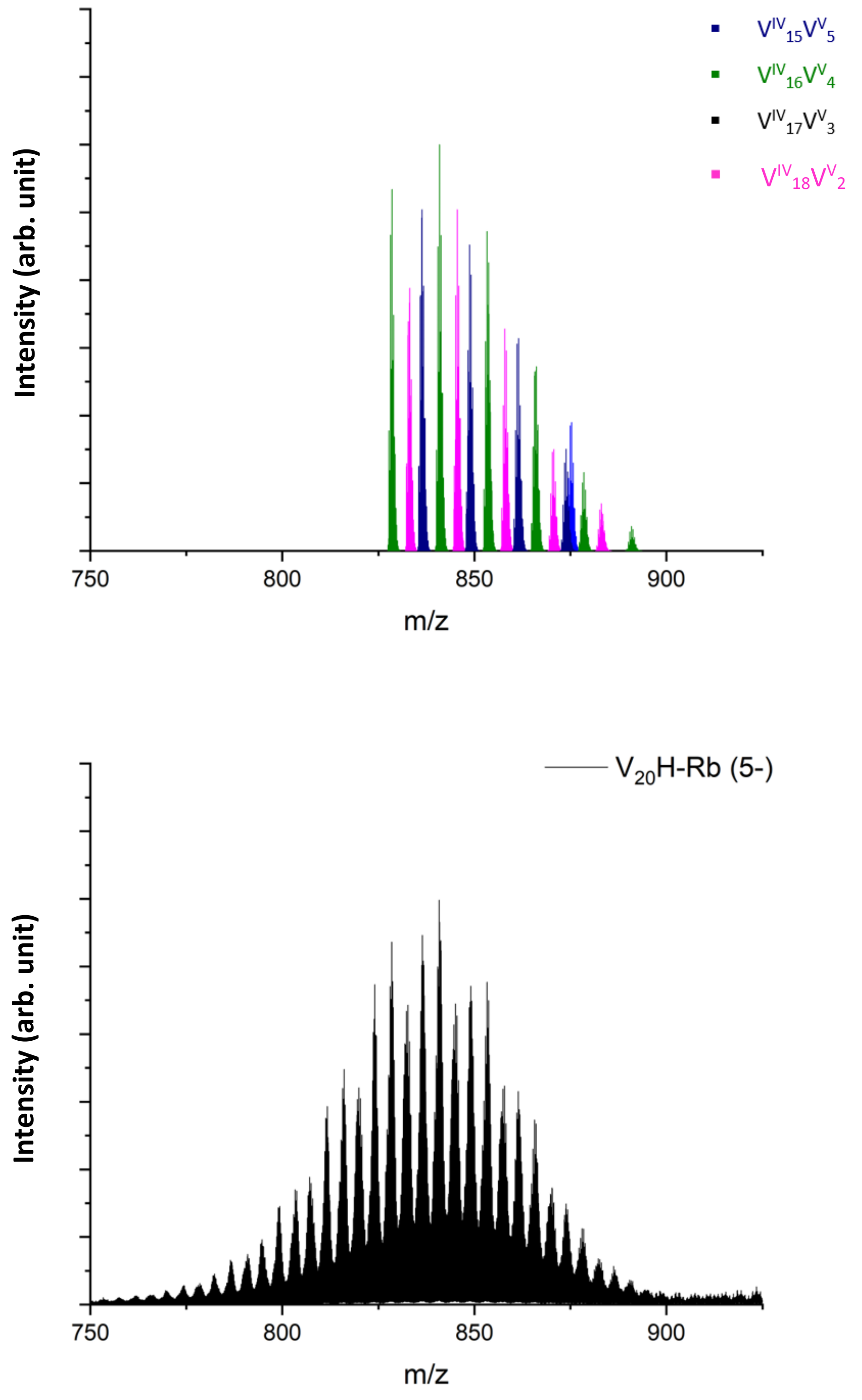

Supplementary Figure 19. The experimental envelope arising from -5 charged species in the ESI-MS spectrum of **Rb-{V_{20}}** (*bottom*) and the modelled envelope (*top*). Different colours represent species with various oxidation states.

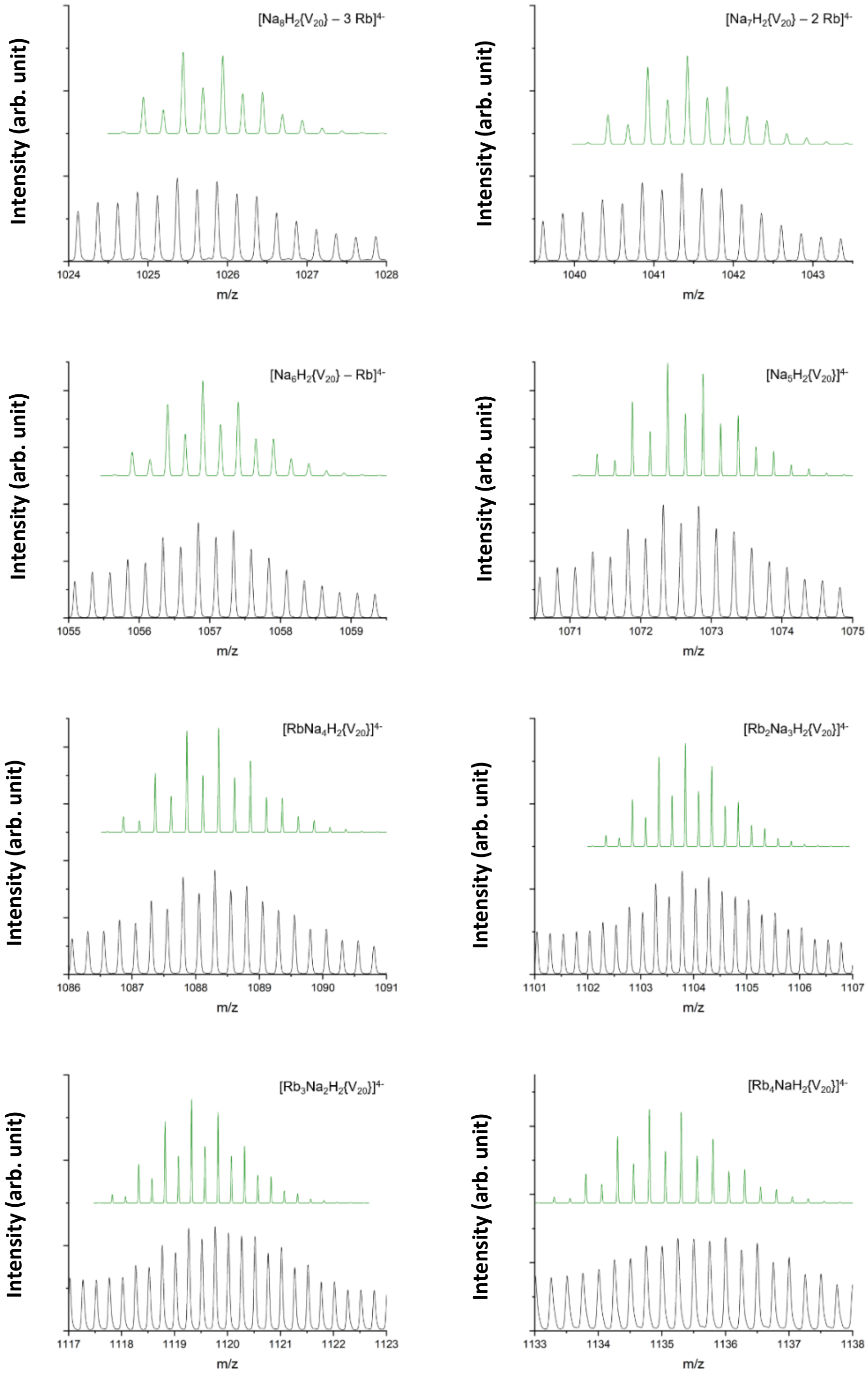

Supplementary Figure 20. Simulated (*top*) and experimental (*bottom*) isotopic patterns for $\{V^{IV}_{16}V^{V}_{4}\}$ species of the envelope arising from -5 charged species in the ESI-MS spectrum of **Rb-{V$_{20}$}**.

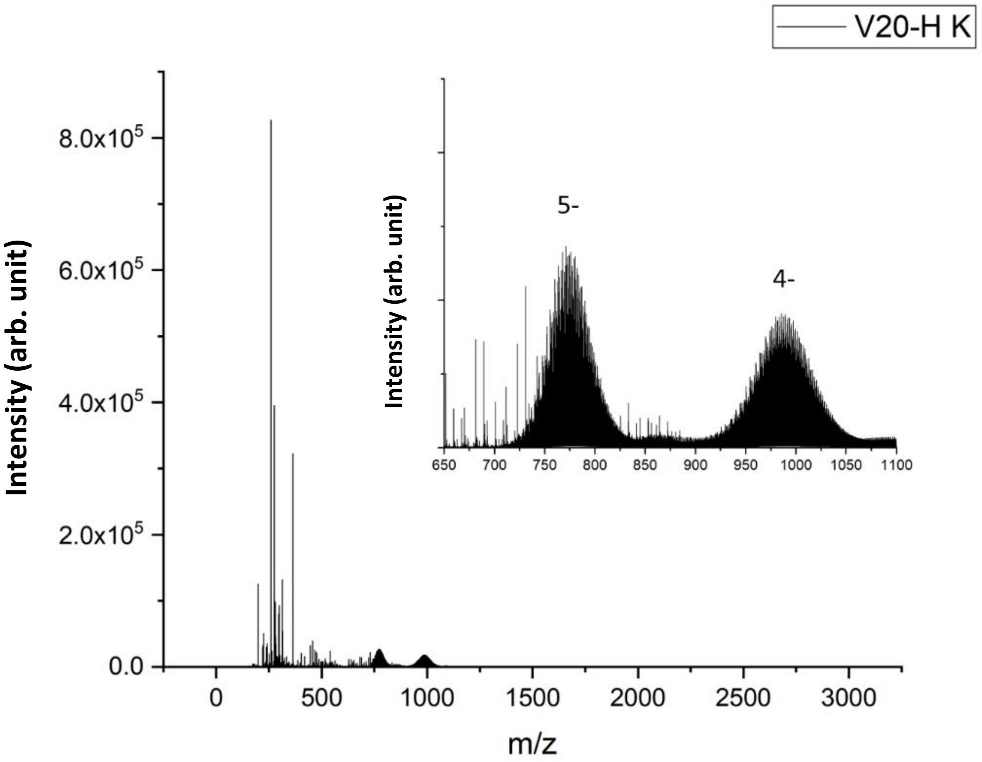

Supplementary Figure 21. ESI-MS spectrum of **K-{V_{20}}** in water. Two broad envelopes corresponding to the -5 and -4 charged species are detectable along with fragments in the lower mass region.

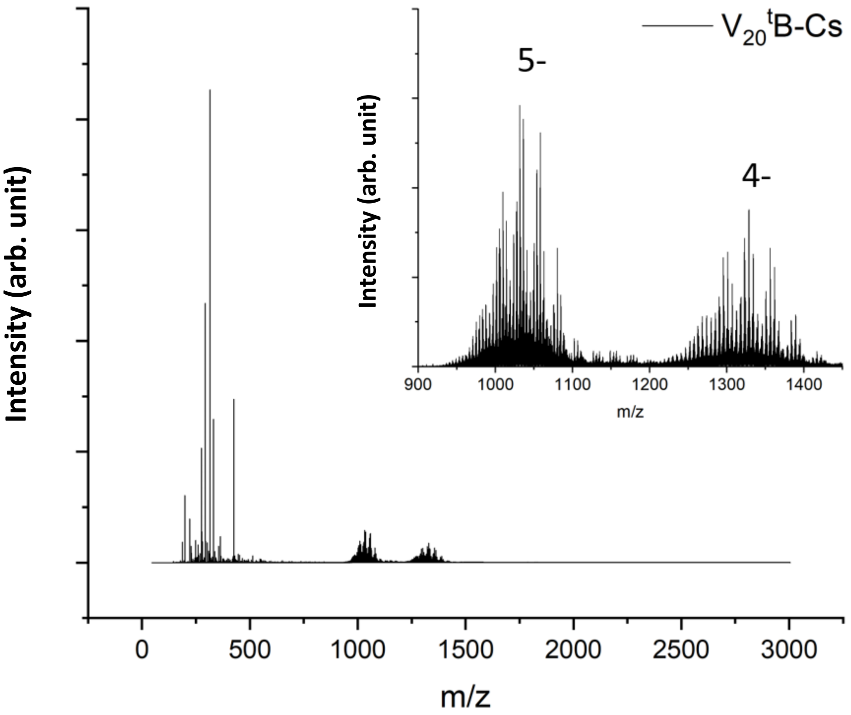

Supplementary Figure 22. ESI-MS spectrum of **Cs-{V_{20}-*t*Bu}** in water. Two broad envelopes corresponding to the -5 and -4 charged species are detectable along with fragments in the lower mass region.

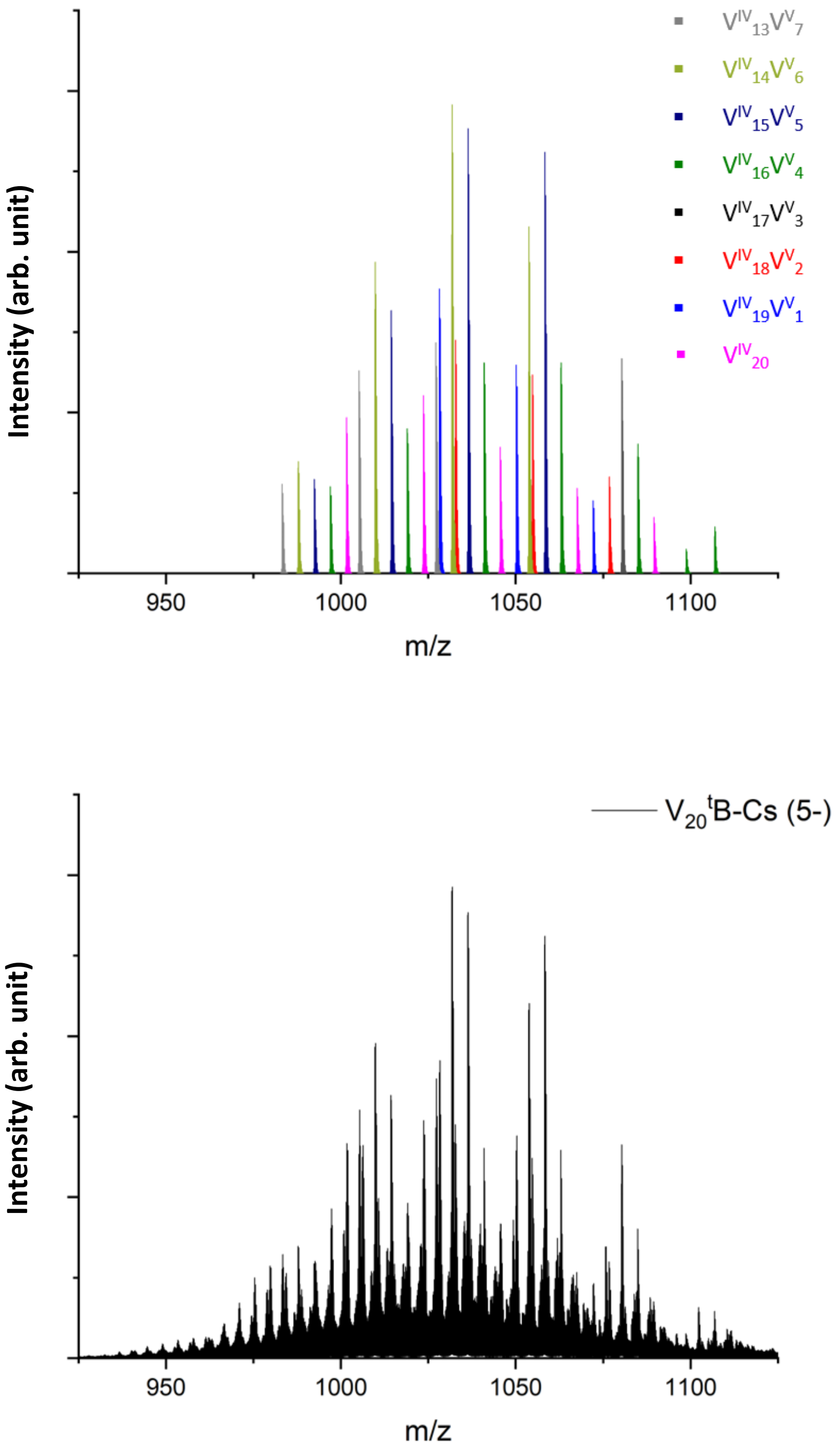

Supplementary Figure 23. The experimental envelope arising from -5 charged species in the ESI-MS spectrum of **Cs-{V_{20}-*t*Bu}** (*bottom*) and the modelled envelope (*top*). Different colours represent species with various oxidation states.

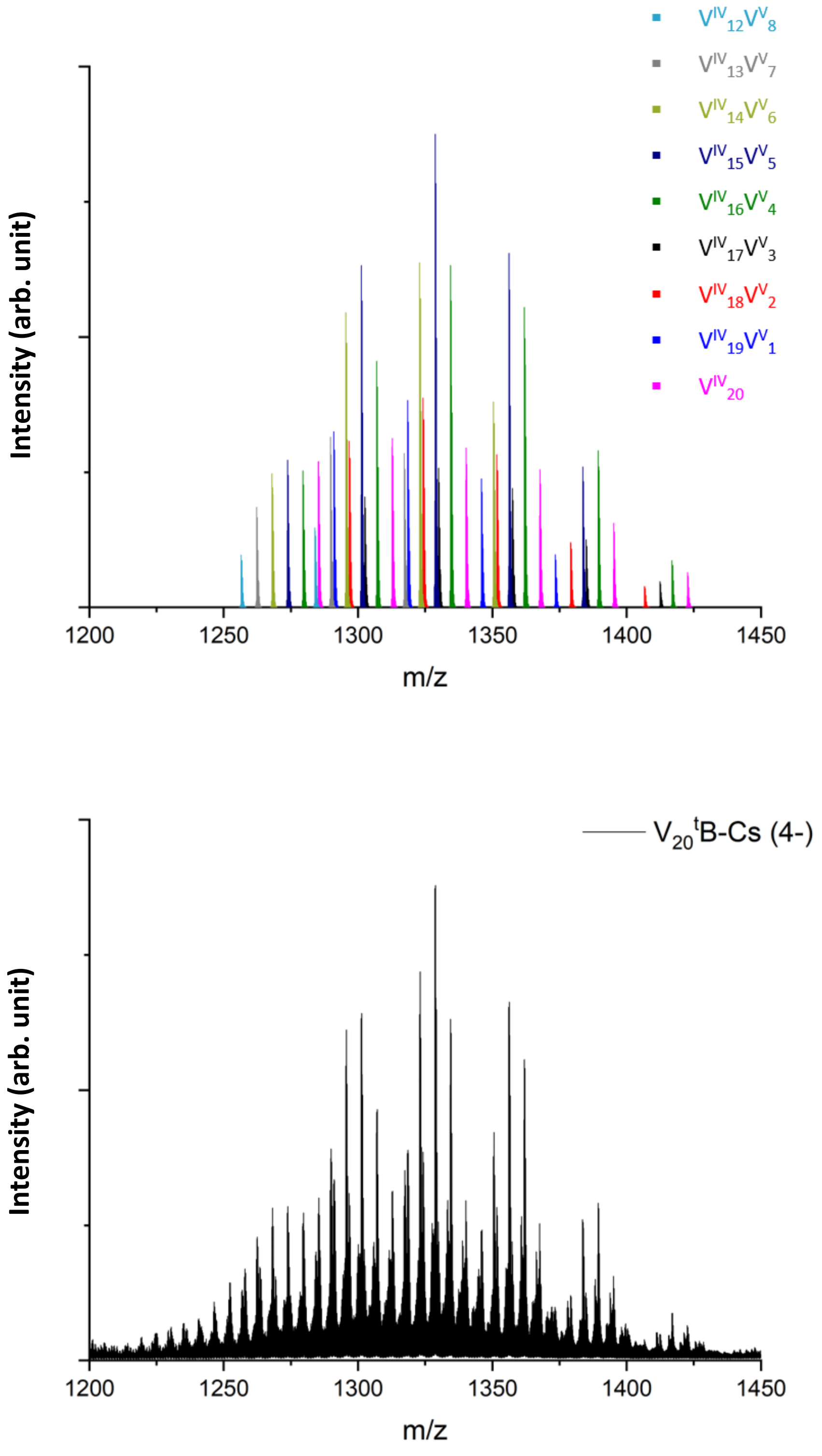

Supplementary Figure 24. The experimental envelope arising from -4 charged species in the ESI-MS spectrum of **Cs-{V_{20}-*t*Bu}** (*bottom*) and the modelled envelope (*top*). Different colours represent species with various oxidation states.

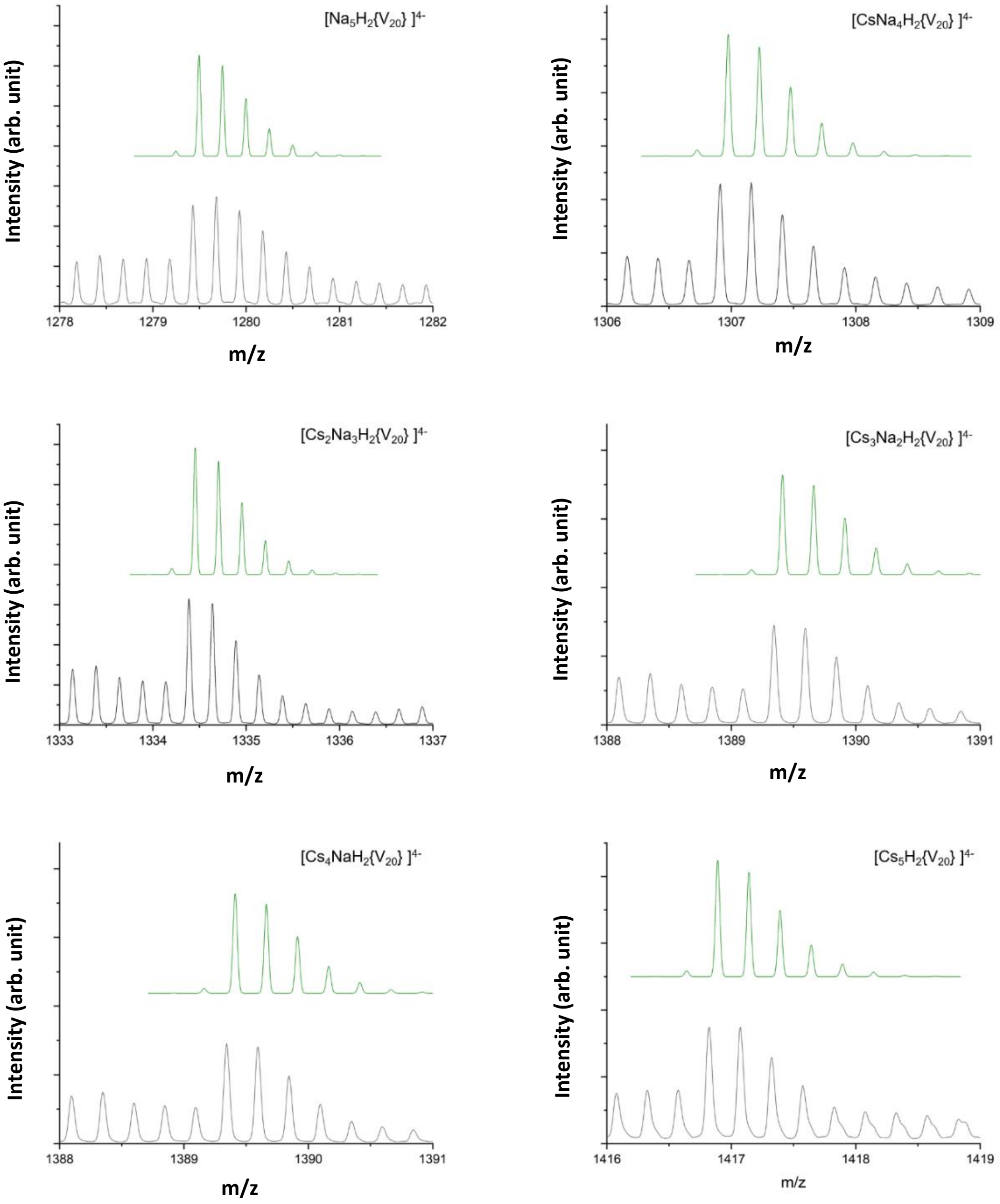

Supplementary Figure 25. Simulated (*top*) and experimental (*bottom*) isotopic patterns for $\{V^{IV}_{16}V^{V}_{4}\}$ species of the envelope arising from -5 charged species in the ESI-MS spectrum of **Cs-{V_{20}-*t*Bu}**.

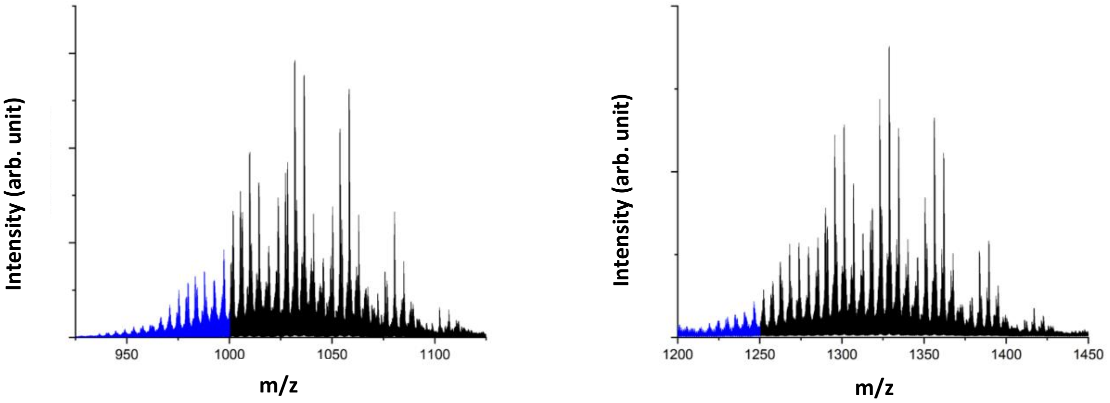

Supplementary Figure 26. Envelopes arising from -5 and -4 charged species of **Cs-{V_{20}-*t*Bu}**. The blue parts of the spectra highlight signals of species which have lost Cs^+ ions from the central cubic alkali metal moiety of the cage structure.

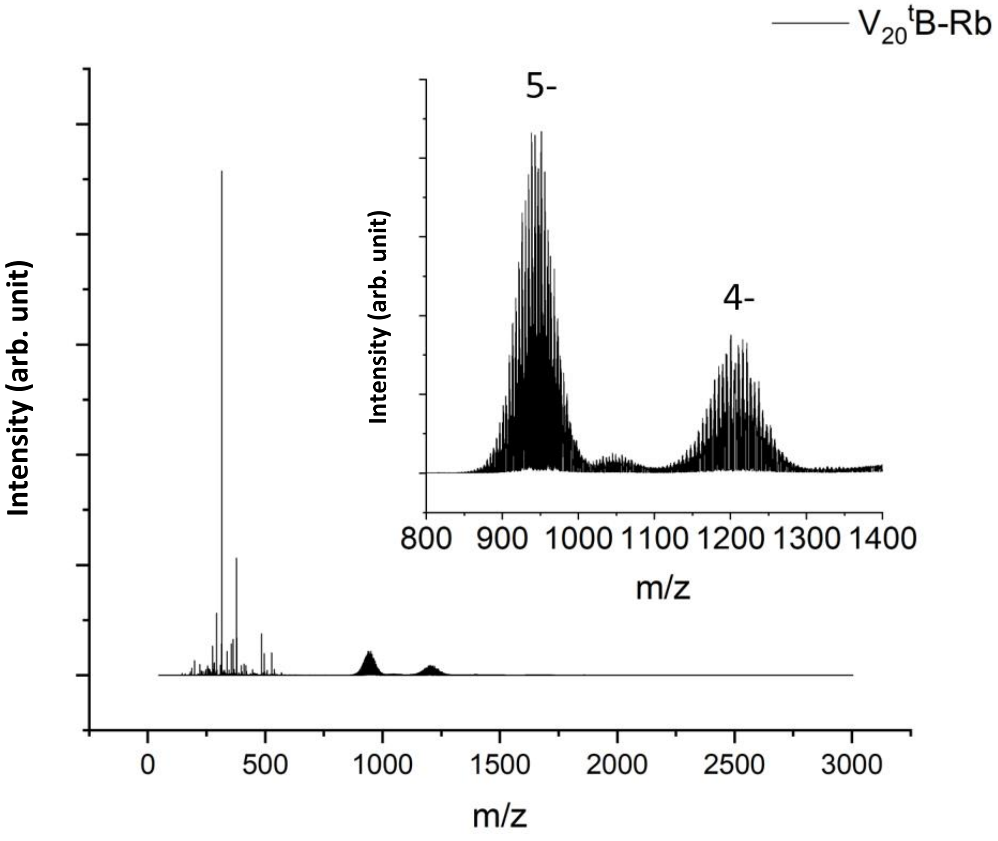

Supplementary Figure 27. ESI-MS spectrum of **Rb-{V_{20}-*t*Bu}** in water. Two broad envelopes corresponding to the -5 and -4 charged species are detectable along with fragments in the lower mass region.

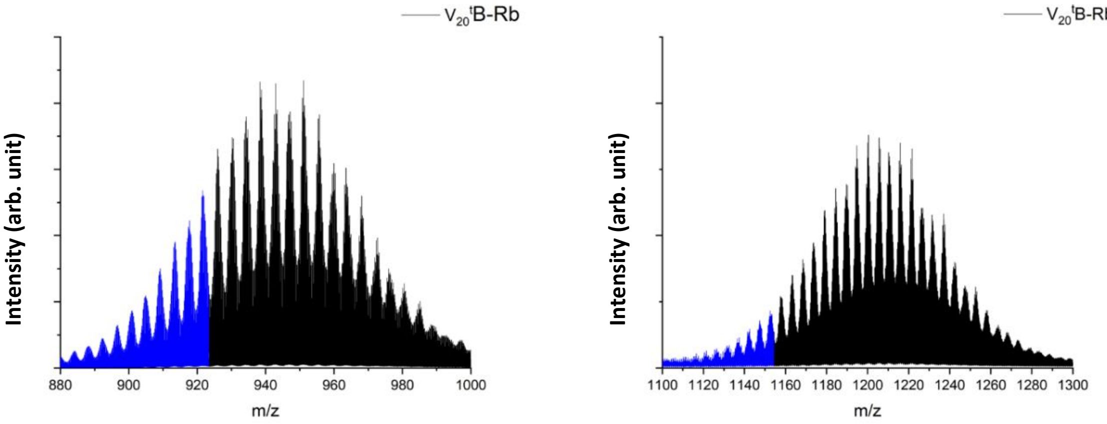

Supplementary Figure 28. Envelopes arising from -5 and -4 charged species of **Rb-{V_{20}-*t*Bu}**. The blue parts of the spectra highlights signals of species which have lost Rb^+ ions from the central cubic alkali metal moiety of the cage structure.

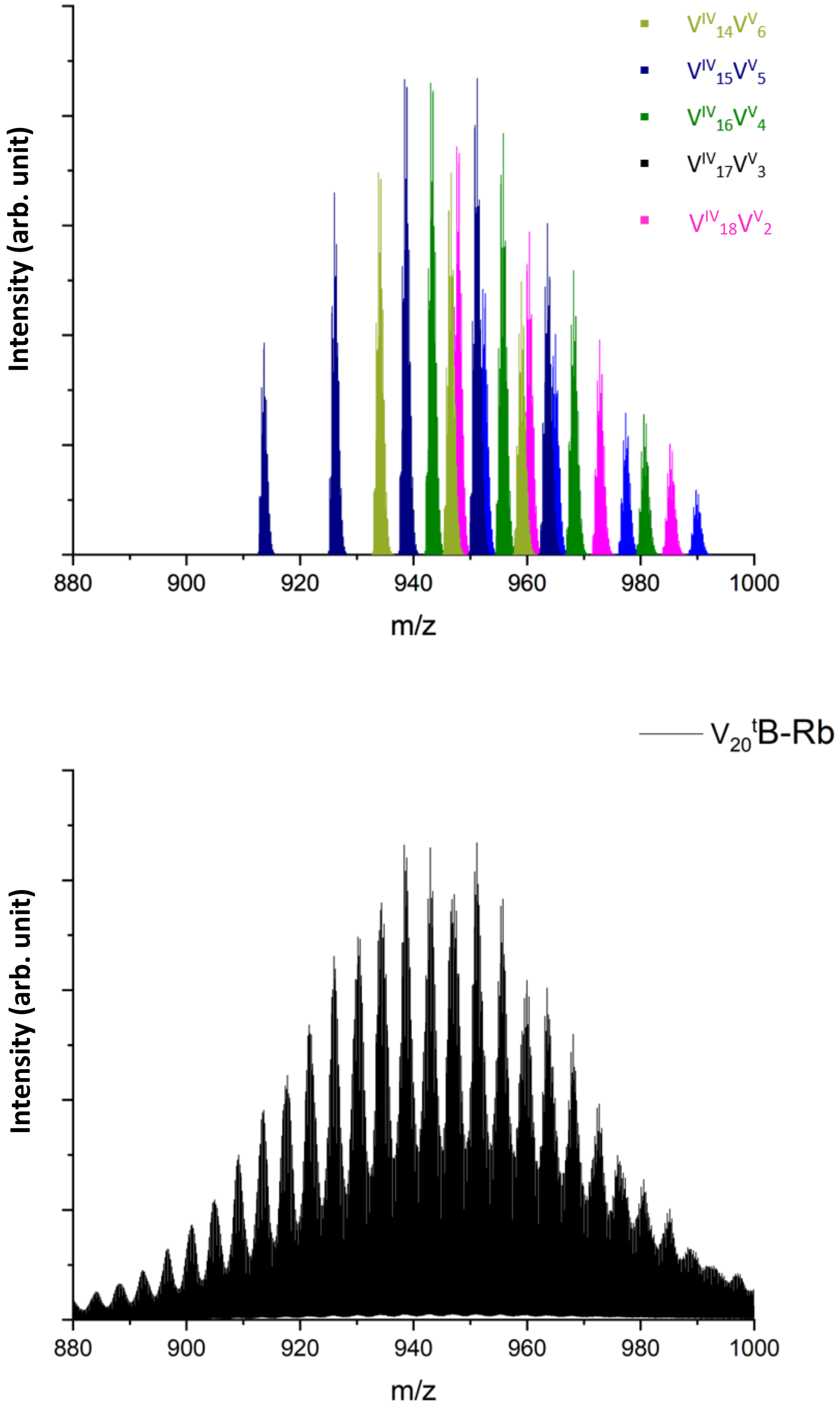

Supplementary Figure 29. The experimental envelope arising from -5 charged species in the ESI-MS spectrum of **Rb-{V_{20}-*t*Bu}** (*bottom*) and the modelled envelope (*top*). Different colours represent species with various oxidation states.

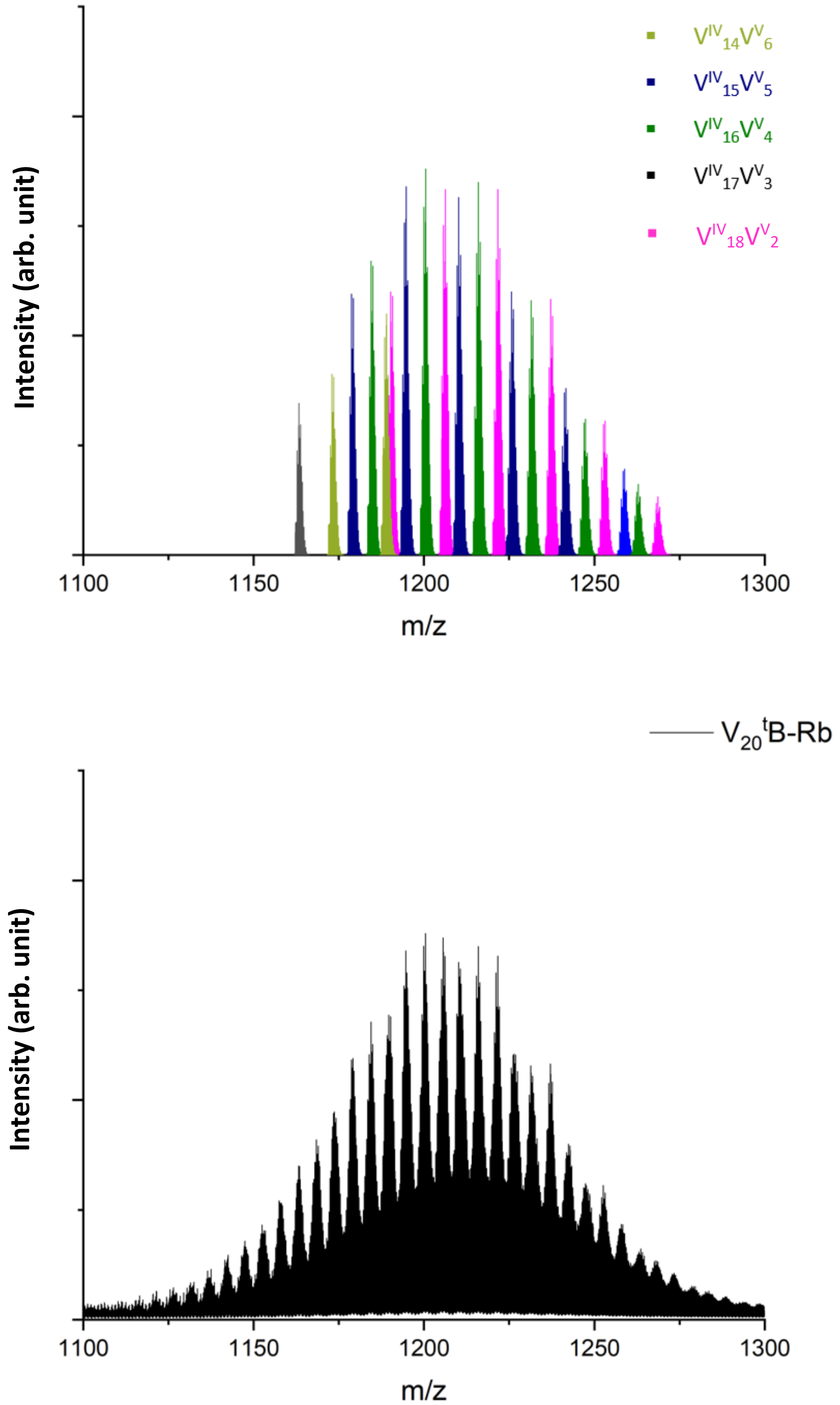

Supplementary Figure 30. The experimental envelope arising from -4 charged species in the ESI-MS spectrum of **Rb-{V_{20}-*t*Bu}** (*bottom*) and the modelled envelope (*top*). Different colours represent species with various oxidation states.

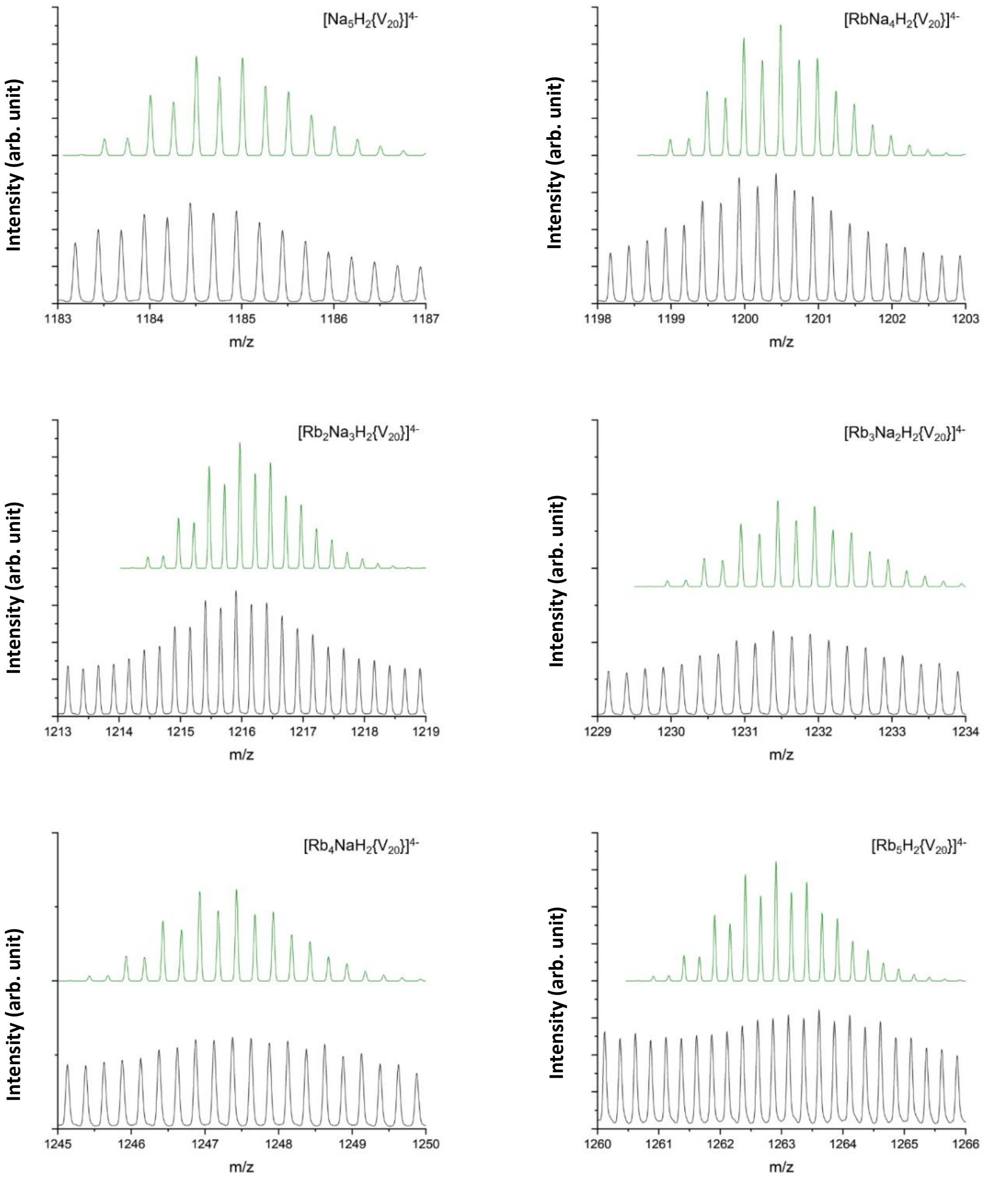

Supplementary Figure 31. Simulated (*top*) and experimental (*bottom*) isotopic patterns for $\{V^{IV}{}_{16}V^{V}{}_{4}\}$ species in envelope arising from -4 charged species in the ESI-MS spectrum of **Rb-{V_{20}-*t*Bu}**.

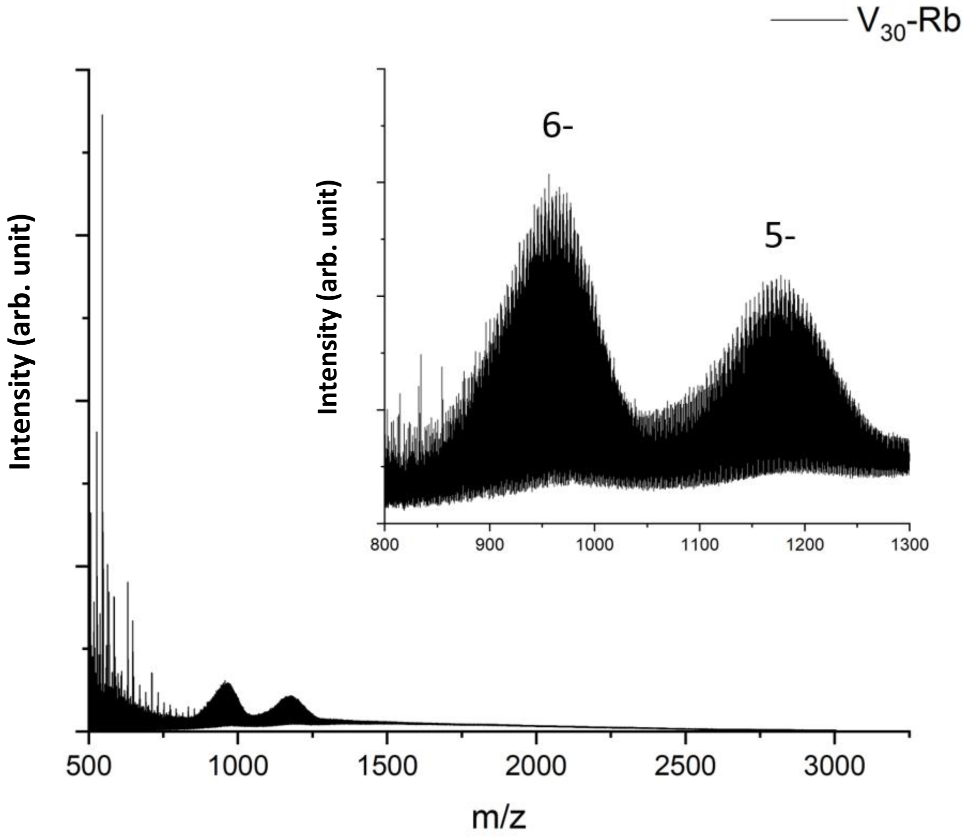

Supplementary Figure 32. ESI-MS spectrum of **Rb-{V_{30}}** in water. Two broad envelopes corresponding to the -5 and -6 charged species are detectable along with fragments in the lower mass region.

K-$\{V_{30}\}$

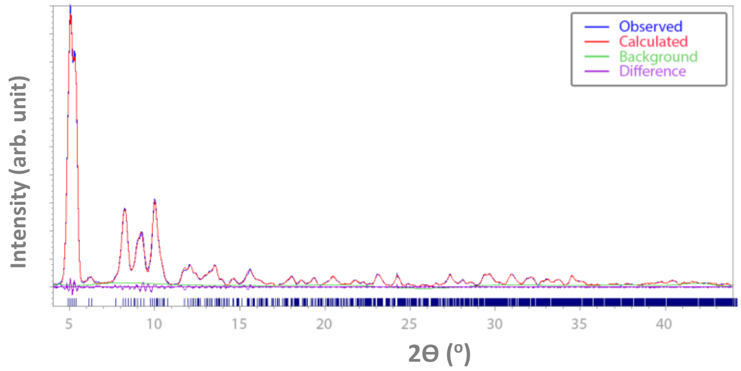

K-$\{V_{20}\}$

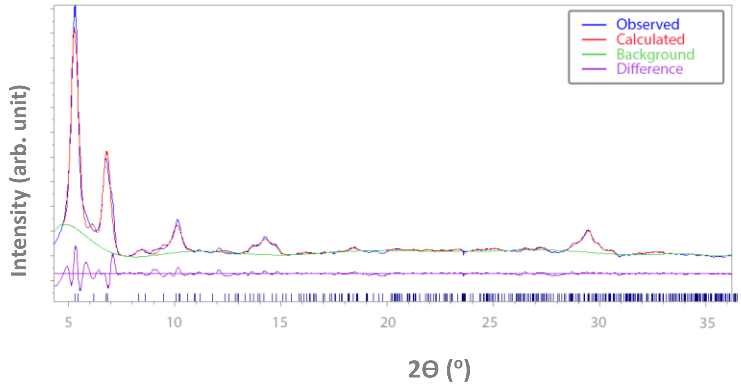

Rb-$\{V_{20}\}$

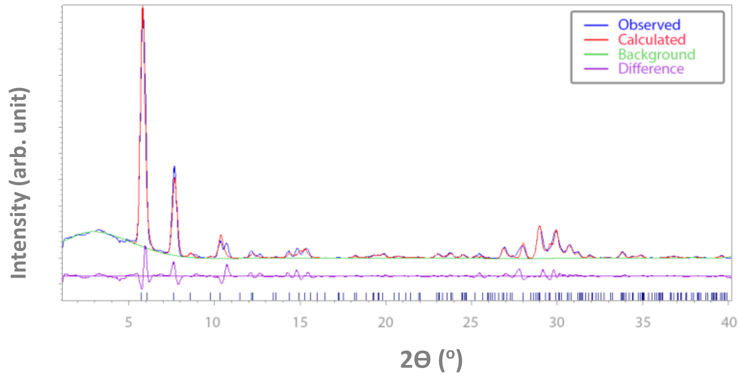

Cs-$\{V_{20}\}$

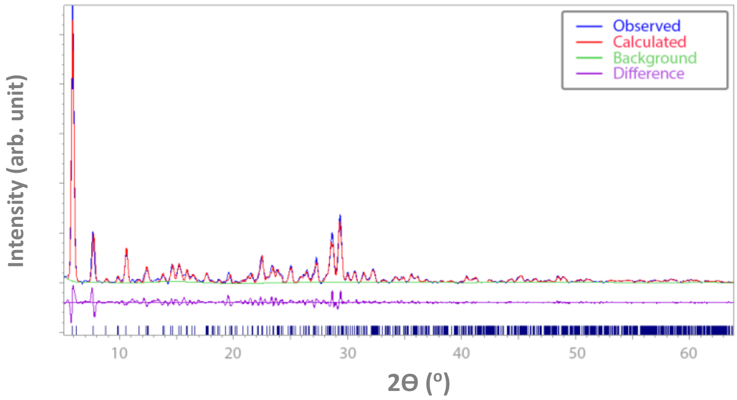

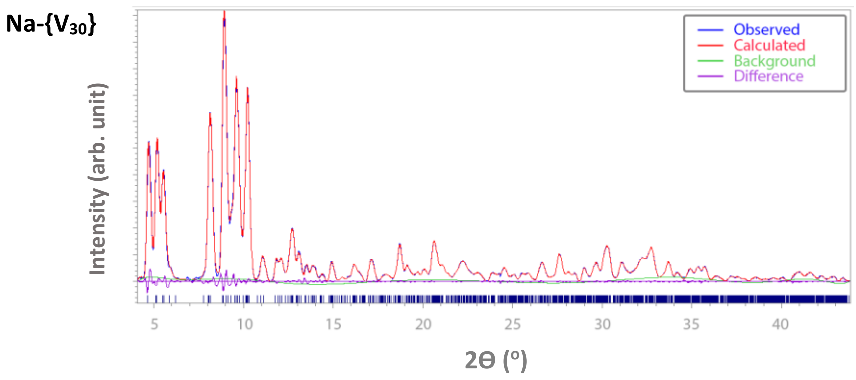

Supplementary Figure 33. Powder X-ray diffraction patterns for various vanadate cages.

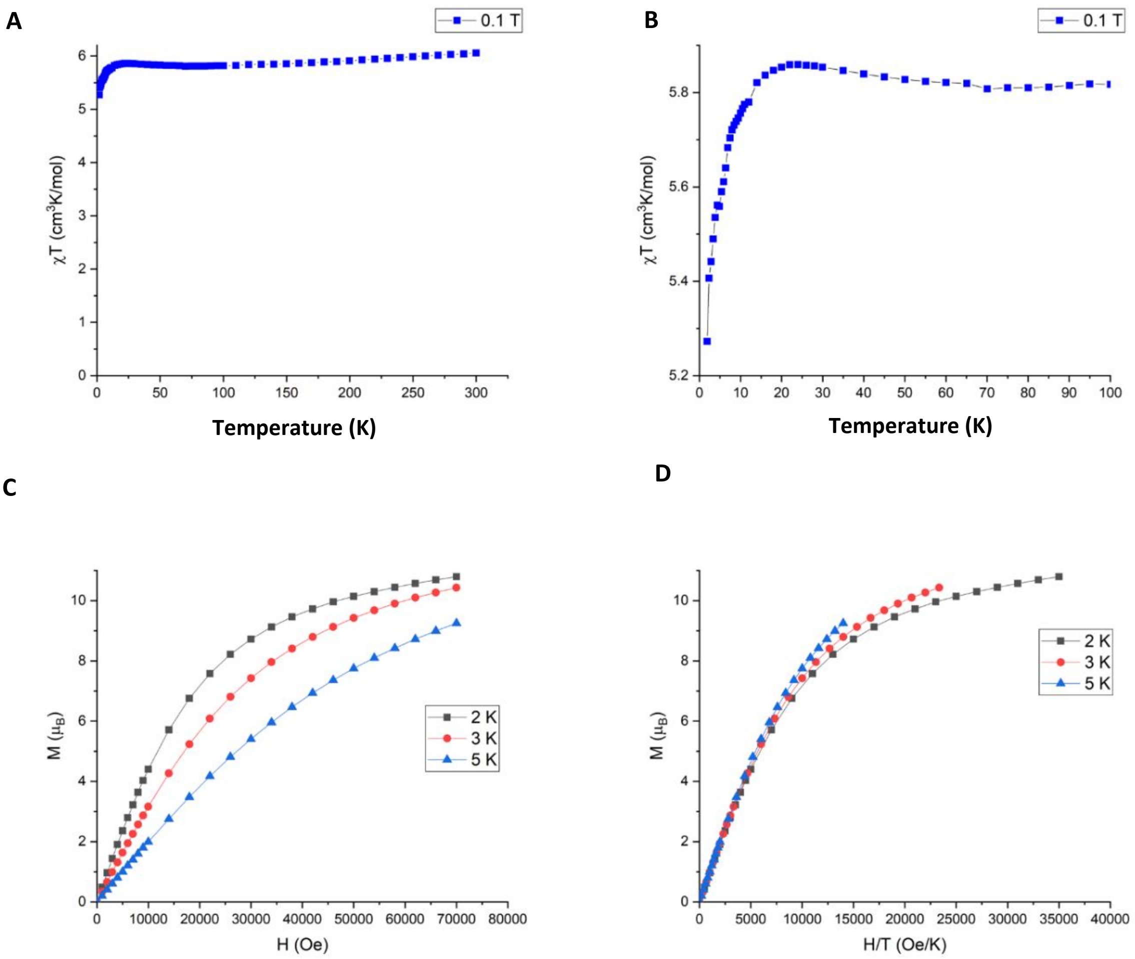

Supplementary Figure 34. Magnetic properties of {V_{20}} ring system**: A & B** Temperature-dependent susceptibility measurements were conducted on a representative sample of **Cs-{V_{20}}** between 300 K to 1.8 K at an applied static dc field of 0.1 T. The room temperature χT value of *ca*. 6.04 cm^3K/mol is consistent with 16 unpaired electrons (S = ½, g = 2, C = 0.375 cm^3K/mol; 16 x 0.375 cm^3K/mol = 6.0 cm^3K/mol) that derive from the 16 V^{IV} d^1 ions in the four {V_5O_9} units. Below 25K, the χT product exhibits a sharp decrease which can be attributed to pre-dominant antiferromagnetic interactions, Zeeman saturation and ligand-field splitting effects. The residual χT value at 1.8 K may be indicative of a non-zero ground state and the presence uncompensated magnetic moments due to competing antiferromagnetic and ferromagnetic interactions. **C & D** The field-dependence of magnetisation was measured from 0 T to 7 T at 2, 3 and 5 K. The lack of saturation of the magnetisation at these temperatures is consistent with χT data at low temperature, low-lying exited states and magnetic anisotropy.

Supplementary Tables

Supplementary Table 1. Assignment of the signals of the envelope arising from the -5 charged species in the ESI-MS spectrum of **Cs-{V_{20}}**.

**Counter ions**	**Oxidation state**	**Calculated (m/z)**	**Peak (m/z)**	**Charge**
**$Na_{10}H_2$ -2Cs**	V^{IV}_{20}	903.7235	903.6642	5-
**Na_9H_2 -Cs**	V^{IV}_{20}	925.7067	925.6422	5-
**Na_8H_2**	V^{IV}_{20}	947.6898	947.6272	5-
**$CsNa_7H_2$**	V^{IV}_{20}	969.6729	969.6077	5-
**$Cs_2Na_6H_2$**	V^{IV}_{20}	991.6561	991.5917	5-
**Na_8H_3 -2Cs**	$V^{IV}_{19}V^{V}_{1}$	894.7292	894.6691	5-
**Na_9H_2 -2Cs**	$V^{IV}_{19}V^{V}_{1}$	899.1256	899.0634	5-
**Na_8H_2 -Cs**	$V^{IV}_{19}V^{V}_{1}$	921.1087	921.0477	5-
**Na_7H_2**	$V^{IV}_{19}V^{V}_{1}$	943.0919	943.0295	5-
**$CsNa_6H_2$**	$V^{IV}_{19}V^{V}_{1}$	965.075	965.0128	5-
**$Cs_2Na_5H_2$**	$V^{IV}_{19}V^{V}_{1}$	987.0581	986.9935	5-
**Na_7H_2 -Cs**	$V^{IV}_{18}V^{V}_{2}$	916.5108	916.4522	5-
**Na_6H_2**	$V^{IV}_{18}V^{V}_{2}$	938.4939	938.4322	5-
**$CsNa_5H_2$**	$V^{IV}_{18}V^{V}_{2}$	960.477	960.4168	5-
**Na_5H_2**	$V^{IV}_{17}V^{V}_{3}$	933.896	933.8383	5-
**$CsNa_4H_2$**	$V^{IV}_{17}V^{V}_{3}$	955.8791	955.8252	5-
**Na4H2**	$V^{IV}_{16}V^{V}_{4}$	929.299	929.246	5-
**$CsNa_3H_2$**	$V^{IV}_{16}V^{V}_{4}$	951.2811	951.2285	5-
**$Cs_2Na_2H_2$**	$V^{IV}_{16}V^{V}_{4}$	973.2643	973.2105	5-
**Cs_3NaH_2**	$V^{IV}_{16}V^{V}_{4}$	995.2474	995.1916	5-
**Na_4H_2 -Cs**	$V^{IV}_{15}V^{V}_{5}$	902.7169	902.6663	5-
**Na_3H_2**	$V^{IV}_{15}V^{V}_{5}$	924.7	924.6485	5-
**$CsNa_2H_2$**	$V^{IV}_{15}V^{V}_{5}$	946.6832	946.6309	5-
**Cs_2NaH_2**	$V^{IV}_{15}V^{V}_{5}$	968.6663	968.6137	5-
**Cs_3H_2**	$V^{IV}_{15}V^{V}_{5}$	990.6494	990.5959	5-
**Na_3H_2 -Cs**	$V^{IV}_{14}V^{V}_{6}$	898.119	898.0673	5-
**Na_2H2**	$V^{IV}_{14}V^{V}_{6}$	920.1021	920.0495	5-
**$CsNaH_2$**	$V^{IV}_{14}V^{V}_{6}$	942.0852	942.0315	5-
**Cs_2H_2**	$V^{IV}_{14}V^{V}_{6}$	964.0684	964.0142	5-

Supplementary Table 2. Assignment of the signals of the envelope arising from the -4 charged species in the ESI-MS spectrum of **Cs-{V_{20}}**.

Counter ions	Oxidation state	Calculated (m/z)	Peak (m/z)	Charge
**$Na_{11}H_2$ - 2Cs**	V^{IV}_{20}	1135.402	1135.328	4-
**$Na_{10}H_2$ - Cs**	V^{IV}_{20}	1162.881	1162.807	4-
**Na_9H_2**	V^{IV}_{20}	1190.36	1190.283	4-
**$CsNa_8H_2$**	V^{IV}_{20}	1217.839	1217.766	4-
**$Cs_2Na_7H_2$**	V^{IV}_{20}	1245.318	1245.241	4-
**$Na_{10}H_2$ - 2Cs**	$V^{IV}_{19}V^{V}_{1}$	1129.655	1129.581	4-
**Na_9H_2 - Cs**	$V^{IV}_{19}V^{V}_{1}$	1157.134	1157.057	4-
**Na_8H_2**	$V^{IV}_{19}V^{V}_{1}$	1184.613	1184.54	4-
**$CsNa_7H_2$**	$V^{IV}_{19}V^{V}_{1}$	1212.092	1212.018	4-
**$Cs_2Na_6H_2$**	$V^{IV}_{19}V^{V}_{1}$	1239.57	1239.493	4-
**$Cs_3Na_5H_2$**	$V^{IV}_{19}V^{V}_{1}$	1267.049	1266.977	4-
**Na_9H_2 - 2Cs**	$V^{IV}_{18}V^{V}_{2}$	1123.907	1123.829	4-
**Na_8H_2 - Cs**	$V^{IV}_{18}V^{V}_{2}$	1151.386	1151.308	4-
**Na_7H_2**	$V^{IV}_{18}V^{V}_{2}$	1178.865	1178.79	4-
**$CsNa_6H_2$**	$V^{IV}_{18}V^{V}_{2}$	1206.344	1206.273	4-
**$Cs_2Na_5H_2$**	$V^{IV}_{18}V^{V}_{2}$	1233.823	1233.756	4-
**$Cs_3Na_4H_2$**	$V^{IV}_{18}V^{V}_{2}$	1261.302	1261.232	4-
**$Cs_4Na_3H_2$**	$V^{IV}_{18}V^{V}_{2}$	1288.781	1288.708	4-
**Na_8H_2 - 2Cs**	$V^{IV}_{17}V^{V}_{3}$	1118.16	1118.085	4-
**Na_7H_2 - Cs**	$V^{IV}_{17}V^{V}_{3}$	1145.639	1145.567	4-
**Na_6H_2**	$V^{IV}_{17}V^{V}_{3}$	1173.118	1173.048	4-
**$CsNa_5H_2$**	$V^{IV}_{17}V^{V}_{3}$	1200.597	1200.528	4-
**$Cs_2Na_4H_2$**	$V^{IV}_{17}V^{V}_{3}$	1228.076	1228.005	4-
**$Cs_3Na_3H_2$**	$V^{IV}_{17}V^{V}_{3}$	1255.555	1255.486	4-
**$Cs_4Na_2H_2$**	$V^{IV}_{17}V^{V}_{3}$	1283.033	1282.964	4-
**Na_6H_2 - Cs**	$V^{IV}_{16}V^{V}_{4}$	1139.891	1139.82	4-
**Na_5H_2**	$V^{IV}_{16}V^{V}_{4}$	1167.37	1167.306	4-
**$CsNa_4H_2$**	$V^{IV}_{16}V^{V}_{4}$	1194.849	1194.781	4-
**$Cs_2Na_3H_2$**	$V^{IV}_{16}V^{V}_{4}$	1222.328	1222.26	4-
**$Cs_3Na_2H_2$**	$V^{IV}_{16}V^{V}_{4}$	1249.807	1249.74	4-
**Cs_4NaH_2**	$V^{IV}_{16}V^{V}_{4}$	1277.286	1277.218	4-
**Na_5H_2 - Cs**	$V^{IV}_{15}V^{V}_{5}$	1134.144	1134.075	4-
**Na_4H_2**	$V^{IV}_{15}V^{V}_{5}$	1161.623	1161.051	4-
**$CsNa_3H_2$**	$V^{IV}_{15}V^{V}_{5}$	1189.102	1189.031	4-

**$Cs_2Na_2H_2$**	$V^{IV}_{15}V^{V}_{5}$	1216.581	1216.515	4-
**Cs_3NaH_2**	$V^{IV}_{15}V^{V}_{5}$	1244.06	1243.993	4-
**Na_4H_2 -Cs**	$V^{IV}_{14}V^{V}_{6}$	1128.396	1128.331	4-
**Na_3H_2**	$V^{IV}_{14}V^{V}_{6}$	1155.875	1155.805	4-
**$CsNa_2H_2$**	$V^{IV}_{14}V^{V}_{6}$	1183.354	1183.286	4-
**Cs_2NaH_2**	$V^{IV}_{14}V^{V}_{6}$	1210.833	1210.763	4-

Supplementary Table 3. Assignment of the signals of the envelope arising from the -4 charged species in the ESI-MS spectrum of **Rb-{V_{20}}**.

Counter ions	Oxidation state	Calculated (m/z)	Peak (m/z)	Charge
**Na_7H_2**	$V^{IV}_{18}V^{V}_{2}$	1083.877	1083.814	4-
**$RbNa_6H_2$**	$V^{IV}_{18}V^{V}_{2}$	1099.858	1099.792	4-
**$Rb_2Na_5H_2$**	$V^{IV}_{18}V^{V}_{2}$	1115.338	1115.279	4-
**$Rb_3Na_4H_2$**	$V^{IV}_{18}V^{V}_{2}$	1130.819	1130.762	4-
**Na_9H_2 - 3 Rb**	$V^{IV}_{17}V^{V}_{3}$	1031.188	1031.116	4-
**Na_8H_2 - 2 Rb**	$V^{IV}_{17}V^{V}_{3}$	1047.169	1047.101	4-
**Na_7H_2 - Rb**	$V^{IV}_{17}V^{V}_{3}$	1062.649	1062.584	4-
**Na_6H_2**	$V^{IV}_{17}V^{V}_{3}$	1078.13	1078.064	4-
**$RbNa5H_2$**	$V^{IV}_{17}V^{V}_{3}$	1094.11	1094.045	4-
**$Rb_2Na_4H_2$**	$V^{IV}_{17}V^{V}_{3}$	1109.591	1109.531	4-
**$Rb_3Na_3H_2$**	$V^{IV}_{17}V^{V}_{3}$	1125.071	1125.013	4-
**$Rb_4Na_2H_2$**	$V^{IV}_{17}V^{V}_{3}$	1140.552	1140.504	4-
**Na_8H_2 – 3 Rb**	$V^{IV}_{16}V^{V}_{4}$	1025.441	1025.369	4-
**Na_7H_2 – 2 Rb**	$V^{IV}_{16}V^{V}_{4}$	1041.421	1041.354	4-
**Na_6H_2 – Rb**	$V^{IV}_{16}V^{V}_{4}$	1056.902	1056.839	4-
**Na_5H_2**	$V^{IV}_{16}V^{V}_{4}$	1072.382	1072.316	4-
**$RbNa_4H_2$**	$V^{IV}_{16}V^{V}_{4}$	1088.363	1088.3	4-
**$Rb2Na_3H_2$**	$V^{IV}_{16}V^{V}_{4}$	1103.843	1103.784	4-
**$Rb_3Na_2H_2$**	$V^{IV}_{16}V^{V}_{4}$	1119.324	1119.272	4-
**Rb_4NaH_2**	$V^{IV}_{16}V^{V}_{4}$	1134.804	1134.757	4-
**Na_7H_2 – 3 Rb**	$V^{IV}_{15}V^{V}_{5}$	1019.693	1019.62	4-
**Na_6H_2 – 2 Rb**	$V^{IV}_{15}V^{V}_{5}$	1035.674	1035.611	4-
**Na_5H_2 – Rb**	$V^{IV}_{15}V^{V}_{5}$	1051.154	1051.091	4-
**Na_4H_2**	$V^{IV}_{15}V^{V}_{5}$	1066.635	1066.578	4-

**$RbNa_3H_2$**	$V^{IV}_{15}V^{V}_{5}$	1082.615	1082.557	4-
**$Rb_2Na_2H_2$**	$V^{IV}_{15}V^{V}_{5}$	1098.096	1098.039	4-

Supplementary Table 4. Assignment of the signals of the envelope arising from the -5 charged species in the ESI-MS spectrum of **Rb-{V_{20}}**.

Counter ions	Oxidation state	Calculated (m/z)	Peak (m/z)	Charge
**$RbNa_5H_2$**	$V^{IV}_{18}V^{V}_{2}$	875.2878	875.2343	5-
**Na_7H_2 - 2 Rb**	$V^{IV}_{17}V^{V}_{3}$	833.1367	833.0803	5-
**Na_6H_2 - Rb**	$V^{IV}_{17}V^{V}_{3}$	845.5211	845.4665	5-
**Na_5H_2**	$V^{IV}_{17}V^{V}_{3}$	857.9055	857.8522	5-
**$RbNa_4H_2$**	$V^{IV}_{17}V^{V}_{3}$	870.6899	870.6371	5-
**$Rb_2Na_3H_2$**	$V^{IV}_{17}V^{V}_{3}$	883.0743	883.0244	5-
**Na_6H_2 - 2 Rb**	$V^{IV}_{16}V^{V}_{4}$	828.5387	828.4852	5-
**Na_5H_2 - Rb**	$V^{IV}_{16}V^{V}_{4}$	840.9231	840.8713	5-
**Na_4H_2**	$V^{IV}_{16}V^{V}_{4}$	853.3075	853.2578	5-
**$RbNa_3H_2$**	$V^{IV}_{16}V^{V}_{4}$	866.0919	866.0403	5-
**$Rb_2Na_2H_2$**	$V^{IV}_{16}V^{V}_{4}$	878.4763	878.4271	5-
**Rb_3NaH_2**	$V^{IV}_{16}V^{V}_{4}$	890.8607	890.8148	5-
**Na_4H_2 - Rb**	$V^{IV}_{15}V^{V}_{5}$	836.3252	836.2773	5-
**Na_3H_2**	$V^{IV}_{15}V^{V}_{5}$	848.7096	848.6643	5-
**$RbNa_2H_2$**	$V^{IV}_{15}V^{V}_{5}$	861.494	861.4527	5-
**Rb_2NaH_2**	$V^{IV}_{15}V^{V}_{5}$	873.8784	873.833	5-

Supplementary Table 5. Assignment of the signals of the envelope arising from the -4 charged species in the ESI-MS spectrum of **Cs-{V$_{20}$-*t*Bu}**.

Counter ions	Oxidation state	Calculated (m/z)	Peak (m/z)	Charge
**Na_9H_2**	V^{IV}_{20}	1302.485	1302.413	4-
**$CsNa_8H_2$**	V^{IV}_{20}	1329.964	1329.889	4-
**$Cs_2Na_7H_2$**	V^{IV}_{20}	1357.443	1357.367	4-
**$Cs_3Na_6H_2$**	V^{IV}_{20}	1384.922	1384.845	4-
**$Cs_4Na_5H_2$**	V^{IV}_{20}	1412.4	1412.323	4-
**Na_8H_2**	$V^{IV}_{19}V^{V}_{1}$	1296.738	1296.666	4-
**$CsNa_7H_2$**	$V^{IV}_{19}V^{V}_{1}$	1324.217	1324.139	4-
**$Cs_2Na_6H_2$**	$V^{IV}_{19}V^{V}_{1}$	1351.696	1351.619	4-
**$Cs_3Na_5H_2$**	$V^{IV}_{19}V^{V}_{1}$	1379.175	1379.098	4-
**$Cs_4Na_4H_2$**	$V^{IV}_{19}V^{V}_{1}$	1406.654	1406.58	4-
**Na_7H_2**	$V^{IV}_{18}V^{V}_{2}$	1290.99	1290.917	4-
**$CsNa_6H_2$**	$V^{IV}_{18}V^{V}_{2}$	1318.469	1318.394	4-
**$Cs_2Na_5H_2$**	$V^{IV}_{18}V^{V}_{2}$	1345.948	1345.871	4-
**$Cs_3Na_4H_2$**	$V^{IV}_{18}V^{V}_{2}$	1373.427	1373.357	4-
**Na_6H_2**	$V^{IV}_{17}V^{V}_{3}$	1285.243	1285.168	4-
**$CsNa_5H_2$**	$V^{IV}_{17}V^{V}_{3}$	1312.722	1312.647	4-
**$Cs_2Na_4H_2$**	$V^{IV}_{17}V^{V}_{3}$	1340.201	1340.133	4-
**$Cs_3Na_3H_2$**	$V^{IV}_{17}V^{V}_{3}$	1367.68	1367.615	4-
**$Cs_4Na_2H_2$**	$V^{IV}_{17}V^{V}_{3}$	1395.159	1395.093	4-
**Cs_5NaH_2**	$V^{IV}_{17}V^{V}_{3}$	1422.638	1422.568	4-
**Na_5H_2**	$V^{IV}_{16}V^{V}_{4}$	1279.495	1279.424	4-
**$CsNa_4H_2$**	$V^{IV}_{16}V^{V}_{4}$	1306.974	1306.91	4-
**$Cs_2Na_3H_2$**	$V^{IV}_{16}V^{V}_{4}$	1334.453	1334.385	4-
**$Cs_3Na_2H_2$**	$V^{IV}_{16}V^{V}_{4}$	1361.932	1361.863	4-
**Cs_4NaH_2**	$V^{IV}_{16}V^{V}_{4}$	1389.411	1389.343	4-
**Cs_5H_2**	$V^{IV}_{16}V^{V}_{4}$	1416.89	1416.823	4-
**Na_4H_2**	$V^{IV}_{15}V^{V}_{5}$	1273.748	1273.679	4-
**$CsNa_3H_2$**	$V^{IV}_{15}V^{V}_{5}$	1301.227	1301.163	4-
**$Cs_2Na_2H_2$**	$V^{IV}_{15}V^{V}_{5}$	1328.706	1328.64	4-
**Cs_3NaH_2**	$V^{IV}_{15}V^{V}_{5}$	1356.185	1356.119	4-
**Cs_4H_2**	$V^{IV}_{15}V^{V}_{5}$	1383.664	1383.596	4-
**Na_3H_2**	$V^{IV}_{15}V^{V}_{5}$	1268.001	1267.933	4-
**$CsNa_2H_2$**	$V^{IV}_{15}V^{V}_{5}$	1295.479	1295.411	4-
**Cs_2NaH_2**	$V^{IV}_{15}V^{V}_{5}$	1322.959	1322.895	4-
**Cs_3H_2**	$V^{IV}_{15}V^{V}_{5}$	1350.437	1350.373	4-
**Na_2H_2**	$V^{IV}_{14}V^{V}_{6}$	1262.253	1262.193	4-

**CsNaH₂**	$V^{IV}_{14}V^{V}_{6}$	1289.732	1289.665	4-
**Cs₂H₂**	$V^{IV}_{14}V^{V}_{6}$	1317.211	1317.142	4-
**NaH₂**	$V^{IV}_{13}V^{V}_{7}$	1256.506	1256.445	4-
**CsH2**	$V^{IV}_{13}V^{V}_{7}$	1283.985	1283.926	4-

Supplementary Table 6. Assignment of the signals of the envelope arising from the -4 charged species in the ESI-MS spectrum of **Cs-{V_{20}-*t*Bu}**.

Counter ions	Oxidation state	Calculated (m/z)	Peak (m/z)	Charge
**Na_7H_2**	$V^{IV}_{19}V^{V}_{1}$	1032.792	1032.732	5-
**$CsNa_6H_2$**	$V^{IV}_{19}V^{V}_{1}$	1054.775	1054.713	5-
**$Cs_2Na_5H_2$**	$V^{IV}_{19}V^{V}_{1}$	1076.758	1076.694	5-
**Na_6H_2**	$V^{IV}_{18}V^{V}_{2}$	1028.194	1028.136	5-
**$CsNa_5H_2$**	$V^{IV}_{18}V^{V}_{2}$	1050.177	1050.116	5-
**$Cs_2Na_4H_2$**	$V^{IV}_{18}V^{V}_{2}$	1072.16	1072.098	5-
**Na_6H_2 - Cs**	$V^{IV}_{17}V^{V}_{3}$	1001.613	1001.558	5-
**Na_5H_2**	$V^{IV}_{17}V^{V}_{3}$	1023.596	1023.538	5-
**$CsNa_4H_2$**	$V^{IV}_{17}V^{V}_{3}$	1045.579	1045.523	5-
**$Cs_2Na_3H_2$**	$V^{IV}_{17}V^{V}_{3}$	1067.562	1067.511	5-
**$Cs_3Na_2H_2$**	$V^{IV}_{17}V^{V}_{3}$	1089.546	1089.492	5-
**Na_5H_2 - Cs**	$V^{IV}_{16}V^{V}_{4}$	997.015	996.964	5-
**Na_4H_2**	$V^{IV}_{16}V^{V}_{4}$	1018.998	1018.951	5-
**$CsNa_3H_2$**	$V^{IV}_{16}V^{V}_{4}$	1040.981	1040.931	5-
**$Cs_2Na_2H_2$**	$V^{IV}_{16}V^{V}_{4}$	1062.964	1062.915	5-
**Cs_3NaH_2**	$V^{IV}_{16}V^{V}_{4}$	1084.948	1084.894	5-
**$Cs_3Na_4H_2$**	$V^{IV}_{16}V^{V}_{4}$	1098.741	1098.681	5-
**Cs_4H_2**	$V^{IV}_{16}V^{V}_{4}$	1106.931	1106.876	5-
**Na_4H_2 - Cs**	$V^{IV}_{15}V^{V}_{5}$	992.4171	992.3684	5-
**$CsNa_2H_2$**	$V^{IV}_{15}V^{V}_{5}$	1036.383	1036.335	5-
**Cs_2NaH_2**	$V^{IV}_{15}V^{V}_{5}$	1058.366	1058.316	5-
**Cs_3H_2**	$V^{IV}_{15}V^{V}_{5}$	1080.35	1080.296	5-
**Na_3H_2**	$V^{IV}_{15}V^{V}_{5}$	1014.4	1014.348	5-
**Na_3H_2 - Cs**	$V^{IV}_{14}V^{V}_{6}$	987.8191	987.7711	5-
**Na_2H_2**	$V^{IV}_{14}V^{V}_{6}$	1009.802	1009.75	5-
**$CsNaH_2$**	$V^{IV}_{14}V^{V}_{6}$	1031.785	1031.738	5-
**Cs_2H_2**	$V^{IV}_{14}V^{V}_{6}$	1053.769	1053.721	5-
**Na_2H_2 - Cs**	$V^{IV}_{13}V^{V}_{7}$	983.2212	983.1722	5-
**NaH_2**	$V^{IV}_{13}V^{V}_{7}$	1005.204	1005.156	5-
**CsH_2**	$V^{IV}_{13}V^{V}_{7}$	1027.187	1027.138	5-

Supplementary Table 7. Assignment of the signals of the arising from the -5 charged species in the ESI-MS spectrum of **Rb-{V$_{20}$-*t*Bu}**.

Counter ions	Oxidation state	Calculated (m/z)	Peak (m/z)	Charge
**Na_6H_2**	$V^{IV}{}_{18}V^{V}{}_{2}$	952.2036	952.1489	5-
**$RbNa_5H_2$**	$V^{IV}{}_{18}V^{V}{}_{2}$	964.9880	964.9372	5-
**$Rb_2Na_4H_2$**	$V^{IV}{}_{18}V^{V}{}_{2}$	977.3724	977.3209	5-
**$Rb_3Na_3H_2$**	$V^{IV}{}_{18}V^{V}{}_{2}$	989.7568	989.7096	5-
**Na_5H_2**	$V^{IV}{}_{17}V^{V}{}_{3}$	947.6056	947.5494	5
**$RbNa_4H_2$**	$V^{IV}{}_{17}V^{V}{}_{3}$	960.3900	960.3372	5-
**$Rb_2Na_3H_2$**	$V^{IV}{}_{17}V^{V}{}_{3}$	972.7744	972.7220	5-
**$Rb_3Na_2H_2$**	$V^{IV}{}_{17}V^{V}{}_{3}$	985.1588	985.1123	5-
**Na_4H_2**	$V^{IV}{}_{16}V^{V}{}_{4}$	943.0077	942.9550	5-
**$RbNa_3H_2$**	$V^{IV}{}_{16}V^{V}{}_{4}$	955.7921	955.7421	5
**$Rb_2Na_2H_2$**	$V^{IV}{}_{16}V^{V}{}_{4}$	968.1765	968.1278	5-
**Rb_3NaH_2**	$V^{IV}{}_{16}V^{V}{}_{4}$	980.5609	980.5135	5-
**Na_5H_2 - 2Rb**	$V^{IV}{}_{15}V^{V}{}_{5}$	913.6429	913.5854	5-
**Na_4H_2 - Rb**	$V^{IV}{}_{15}V^{V}{}_{5}$	926.0253	925.9699	5-
**Na_3H_2**	$V^{IV}{}_{15}V^{V}{}_{5}$	938.4097	938.3597	5
**$RbNa_2H_2$**	$V^{IV}{}_{15}V^{V}{}_{5}$	951.1936	951.1460	5-
**Rb_2NaH_2**	$V^{IV}{}_{15}V^{V}{}_{5}$	963.5780	963.5323	5-
**Na_2H_2**	$V^{IV}{}_{14}V^{V}{}_{6}$	933.8118	933.7637	5-
**$NaRbH_2$**	$V^{IV}{}_{14}V^{V}{}_{6}$	946.5962	946.5489	5-
**Rb_2H_2**	$V^{IV}{}_{14}V^{V}{}_{6}$	958.9806	958.9357	5

Supplementary Table 8. Assignment of the signals of the envelope arising from the -4 charged species in the ESI-MS spectrum of **Rb-{V_{20}-*t*Bu}**.

Counter ions	Oxidation state	Calculated (m/z)	Peak (m/z)	Charge
**$Rb_4Na_3H_2$**	$V^{IV}_{18}V^{V}_{2}$	1258.9243	1258.8654	4-
**Na_6H_2**	$V^{IV}_{17}V^{V}_{3}$	1190.2548	1190.1884	4-
**$RbNa_5H_2$**	$V^{IV}_{17}V^{V}_{3}$	1206.2354	1206.1742	4-
**$Rb_2Na_4H_2$**	$V^{IV}_{17}V^{V}_{3}$	1221.7159	1221.6505	4-
**$Rb_3Na_3H_2$**	$V^{IV}_{17}V^{V}_{3}$	1237.1964	1237.1359	4-
**$Rb_4Na_2H_2$**	$V^{IV}_{17}V^{V}_{3}$	1253.1769	1253.1209	4-
**Rb_5NaH_2**	$V^{IV}_{17}V^{V}_{3}$	1268.6574	1268.6016	4-
**Na_5H_2**	$V^{IV}_{16}V^{V}_{4}$	1184.5074	1184.4386	4-
**$RbNa_4H_2$**	$V^{IV}_{16}V^{V}_{4}$	1200.4879	1200.4266	4-
**$Rb_2Na_3H_2$**	$V^{IV}_{16}V^{V}_{4}$	1215.9684	1215.9070	4-
**$Rb_3Na_2H_2$**	$V^{IV}_{16}V^{V}_{4}$	1231.4489	1231.3905	4-
**Rb_4NaH_2**	$V^{IV}_{16}V^{V}_{4}$	1247.4294	1247.3730	4-
**Rb_5H_2**	$V^{IV}_{16}V^{V}_{4}$	1262.9099	1262.8604	4-
**Na_5H_2 - Rb**	$V^{IV}_{15}V^{V}_{5}$	1163.2795	1163.2145	4-
**Na_4H_2**	$V^{IV}_{15}V^{V}_{5}$	1178.7600	1178.6959	4-
**$RbNa_3H_2$**	$V^{IV}_{15}V^{V}_{5}$	1194.7405	1194.6791	4-
**$Rb_2Na_2H_2$**	$V^{IV}_{15}V^{V}_{5}$	1210.2210	1210.1635	4-
**Rb_3NaH_2**	$V^{IV}_{15}V^{V}_{5}$	1225.7015	1225.6447	4-
**Rb_4H_2**	$V^{IV}_{15}V^{V}_{5}$	1241.6820	1241.6314	4-
**Na_3H_2**	$V^{IV}_{14}V^{V}_{6}$	1173.0125	1172.9539	4-
**$RbNa_2H_2$**	$V^{IV}_{14}V^{V}_{6}$	1188.9930	1188.9320	4-

Supplementary Table 9. Bond valence sum (BVS) calculations for **K-{V_{20}}**, **Rb-{V_{20}}** and **Cs-{V_{20}}**.

K-{V_{20}}	BVS	Oxidation state
**V1**	4.36	5
**V2**	4.14	4
**V3**	3.85	4
**V3a**	3.88	4
**V4**	4.06	4
**V5**	4.40	5
**V6**	4.11	4
**V7**	4.02	4
**V7a**	3.84	4

Rb-{V_{20}}	BVS	Oxidation state
**V1**	4.55	5
**V2**	4.00	4
**V3**	3.82	4
**V3a**	3.95	4

Cs-{V_{20}}	BVS	Oxidation state
**V1**	4.56	5
**V2**	4.03	4
**V3**	3.86	4
**V3a**	3.73	4
**V4**	4.02	4
**V4a**	3.78	4

Supplementary Table 10. Bond valence sum (BVS) calculations for **Rb-{V_{20}-*t*Bu}** and **Cs-{V_{20}-*t*Bu}**.

Rb-{V_{20}-*t*Bu}	BVS	Oxidation state		BVS	Oxidation state
**V1**	4.89	5	**V4A**	3.99	4
**V2**	4.13	4	**V5**	3.32	4
**V3**	4.01	4	**V5A**	3.34	4
**V4**	3.71	4			

Cs-{V_{20}-*t*Bu}	BVS	Oxidation state		BVS	Oxidation state
**V1**	4.55	5	**V6**	4.51	5
**V2**	3.99	4	**V7**	4.13	4
**V3**	3.99	4	**V8**	4.16	4
**V4**	3.94	4	**V9**	3.66	4
**V4a**	3.96	4	**V9a**	3.78	4
**V5**	3.83	4	**V10**	4.04	4
**V5a**	3.78	4	**V10a**	3.92	4

Supplementary Table 11. Bond valence sum (BVS) for **Rb-{V_{20}-NH_2}** and **Rb-{V_{20}-Py}** (OS: Oxidation state).

Rb-{V_{20}-NH_2}	BVS	OS
**V1**	4.56	5
**V2**	3.99	4
**V3a**	3.82	4
**V3b**	3.81	4
**V4a**	3.69	4
**V4b**	3.77	4

Rb-{V_{20}-Py}	BVS	OS
**V1**	4.12	4
**V2**	4.06	4
**V3**	3.91	4
**V3a**	3.85	4

Supplementary Table 12. Bond valence sum (BVS) for both isomers of **K-{V_{30}}** (OS: Oxidation state).

	BVS	Ox. St.		BVS	OS		BVS	OS		BVS	OS
**V1**	4.88	5	**V1a**	4.94	5	**V6**	4.95	5	**V11**	5.24	5
**V2**	4.26	4	**V2a**	3.7	4	**V7**	3.92	4	**V12**	3.94	4
**V3**	3.97	4	**V3a**	4.17	4	**V8**	4.08	4	**V12a**	4.09	4
**V4**	3.83	4	**V3b**	4.02	4	**V8a**	4.07	4	**V13**	3.86	4
**V5**	3.8	4	**V4a**	3.87	4	**V9**	3.98	4	**V14**	3.96	4
			**V5a**	4.08	4	**V10**	3.97	4	**V14A**	4.07	4
			**V5b**	3.71	4	**V10a**	3.84	4	**V15**	4.06	4

Supplementary Table 13. Bond valence sum (BVS calculations for **Rb-{V_{30}}** (OS: Oxidation state).

	BVS	OS		BVS	OS		BVS	Ox. St.		BVS	OS
**V1**	4.91	5	**V6**	5.06	5	**V6a**	5.29	5	**V11**	5.1	5
**V2**	3.97	4	**V7**	4.2	4	**V7a**	3.67	4	**V12**	3.84	4
**V3**	4.25	4	**V8**	4.09	4	**V7b**	4.08	4	**V12a**	4.01	4
**V4**	4.13	4	**V9**	4.22	4	**V8a**	4.11	4	**V13**	3.95	4
**V5**	4.53	4	**V10**	3.97	4	**V9a**	4.12	4	**V14**	4.13	4
						**V9b**	4.75	4	**V14A**	3.36	4
						**V10a**	3.85	4	**V15**	4.15	4

Supplementary Table 14. Bond valence sum (BVS) calculations for **Cs-{V_{30}}** (OS: Oxidation state).

	BVS	OS		BVS	OS		BVS	Ox. St.		BVS	OS
**V1**	5.24	5	**V6**	4.87	5	**V11**	5.09	5	**V16**	5.22	5
**V2**	4.33	4	**V7**	4.14	4	**V12**	4.31	4	**V17**	3.67	4
**V3**	4.37	4	**V8**	4.07	4	**V13**	4.57	4	**V18**	4.05	4
**V4**	4.00	4	**V9**	4.15	4	**V14**	4.12	4	**V19**	4.31	4
**V5**	3.81	4	**V10**	4.22	4	**V15**	4.30	4	**V20**	4.13	4
									**V20A**	4.08	4

	BVS	OS		BVS	OS
**V21**	5.38	5	**V26**	5.57	5
**V22**	4.05	4	**V27**	4.30	4
**V23**	4.31	4	**V28**	4.11	4
**V23A**	4.01	4	**V28A**	3.98	4
**V24**	4.32	4	**V29**	4.46	4
**V25**	4.21	4	**V30**	3.77	4
**V25A**	3.95	4			

Supplementary Table 15. Bond valence sum (BVS) calculations for **Na-{V_{30}}** (OS: Oxidation state).

	BVS	OS		BVS	OS		BVS	Ox. St.
**V001**	4.02	4	**V006**	5.04	5	**V00B**	4.05	4
**V002**	4.07	4	**V007**	4.05	4	**V00C**	4.05	4
**V003**	4.97	5	**V008**	4.04	4	**V00D**	4.06	4
**V004**	4.00	4	**V009**	4.03	4	**V00E**	4.05	4
**V005**	4.04	4	**V00A**	4.07	4	**V00F**	5.07	5

Supplementary Table 16. Crystallographic tables for **Cs-{V_{20}}**, **Rb-{V_{20}}** and **K-{V_{20}}**.

**Identification code**	**Cs-{V_{20}}**	**Rb-{V_{20}}**	**K-{V_{20}}**
**Cryst./Empirical formula**	$C_{50}H_{35}Cs_8NNa_{0.5}O_{85}P_{16}V_{20}$	$C_{48}H_{32}Cl_2NaO_{88}P_{16}Rb_8V_{20}$	$C_{48}H_{32}K_9O_{88}P_{16}V_{20}$
**Formula weight(g/mol)**	4598.88	4308.7	3883.21
**Temperature/K**	100(2)	100(2)	100(2)
**Crystal system**	tetragonal	tetragonal	monoclinic
**Space group**	*P4/mnc*	*I4/mmm*	*C2/m*
**a/Å**	19.9912(6)	20.5137(5)	30.2412(12)
**b/Å**	19.9912(6)	20.5137(5)	20.5427(8)
**c/Å**	22.9901(7)	23.0839(7)	17.2658(7)
**α/°**	90	90	90
**β/°**	90	90	110.393(2)
**γ/°**	90	90	90
**Volume/Å^3**	9187.9(6)	9714.0(6)	10053.9(7)
**Z**	2	2	2
**ρ_{calc}g/cm^3**	1.662	1.473	1.283
**μ/mm^{-1}**	2.739	3.135	1.262
**F(000)**	4335	4130	3790
**Crystal size/mm^3**	0.14 × 0.1 × 0.08	0.18 × 0.1 × 0.04	0.13 × 0.13 × 0.13
**Radiation**	MoKα (λ = 0.71073)	MoKα (λ = 0.71073)	MoKα (λ = 0.71073)
**2Θ range for data collection/°**	4.568 to 55.168	4.778 to 59.366	4.85 to 54.642
**Index ranges**	-26 ≤ h ≤ 25, -25 ≤ k ≤ 26, -29 ≤ l ≤ 29	-28 ≤ h ≤ 28 -28 ≤ k ≤ 28 -32 ≤ l ≤ 32	-38 ≤ h ≤ 38 -26 ≤ k ≤ 26 -22 ≤ l ≤ 22
**Reflections collected**	201697	149504	110330
**Independent reflections**	5459 [R_{int} = 0.1310, R_{sigma} = 0.0253]	3832 [R_{int} = 0.0885 R_{sigma} = 0.0199]	11569 [R_{int} = 0.1457, R_{sigma} = 0.0682]
**Data/restraints/ parameters**	5459/27/246	3832/8/127	11569/13/467
**Goodness-of-fit on F^2**	1.033	1.004	1.046
**Final R indexes [I>=2σ (I)]**	R_1 = 0.0507, wR_2 = 0.1143	R_1 = 0.0615, wR_2 = 0.1671	R_1 = 0.0782, wR_2 = 0.2303
**Final R indexes [all data]**	R_1 = 0.0736, wR_2 = 0.1297	R_1 = 0.0783, wR_2 = 0.1854	R_1 = 0.1250, wR_2 = 0.2638
**Largest diff. peak/hole / eÅ^{-3}**	2.17/-1.26	3.07/-2.63	1.38/-0.71
**CCDC Deposition No.**	2350529	2350524	2350523

Supplementary Table 17. Crystallographic tables for **Rb-{V_{20}-tBu}** and **Cs-{V_{20}-tBu}**.

Identification code	Rb-{V_{20}-*t*Bu}	Cs-{V_{20}-*t*Bu}
**Cryst./Empirical formula**	$C_{80}H_{96}Cl_2NaO_{84}P_{16}Rb_8V_{20}$	$C_{80}H_{96}Cl_2Cs_8NaO_{84}P_{16}V_{20}$
**Formula weight/(g/mol)**	4693.54	5073.05
**Temperature/K**	100(2)	100(2)
**Crystal system**	monoclinic	triclinic
**Space group**	*C*2/*m*	*P*-1
**a/Å**	20.4471(12)	17.8917(8)
**b/Å**	33.574(2)	19.7119(9)
**c/Å**	17.9993(18)	20.1522(10)
**α/°**	90	116.487(3)
**β/°**	105.2820(10)	102.900(4)
**γ/°**	90	96.335(3)
**Volume/Å^3**	11919.4(16)	6018.5(5)
**Z**	2	1
**ρ_{calc}g/cm^3**	1.308	1.4
**μ/mm^{-1}**	2.560	17.255
**F(000)**	4578	2433
**Crystal size/mm^3**	0.204 × 0.105 × 0.099	0.11 × 0.09 × 0.06
**Radiation**	MoKα (λ = 0.71073)	CuKα (λ = 1.54178)
**2Θ range for data collection/°**	5.056 to 50.992	5.22 to 117.86
**Index ranges**	-24 ≤ h ≤ 24, -40 ≤ k ≤ 40, -21 ≤ l ≤ 21	-18 ≤ h ≤ 19, -21 ≤ k ≤ 21, -22 ≤ l ≤ 22
**Reflections collected**	92548	47961
**Independent reflections**	11251 [R_{int} = 0.1427, R_{sigma} = 0.0798]	17211 [R_{int} = 0.0837, R_{sigma} = 0.1058]
**Data/restraints/parameters**	11251/98/525	17211/321/960
**Goodness-of-fit on F^2**	1.025	1.02
**Final R indexes [I>=2σ (I)]**	R_1 = 0.0814, wR_2 = 0.2325	R_1 = 0.0687, wR_2 = 0.1875
**Final R indexes [all data]**	R_1 = 0.1375, wR_2 = 0.2776	R_1 = 0.1015, wR_2 = 0.2087
**Largest diff. peak/hole / e Å^{-3}**	1.82/-1.84	1.05/-1.00
**CCDC Deposition No.**	2350525	2350528

Supplementary Table 18. Crystallographic tables for **Rb-{V_{20}-NH_2}** and **Rb-{V_{20}-Py}**.

Identification code	Rb-{V_{20}-NH_2}	Rb-{V_{20}-Py}
Cryst./Empirical formula	$C_{48}H_{40}Cl_2N_8NaO_{84}P_{16}Rb_8V_{20}$	$C_{40}H_{24}Cl_2N_8NaO_{84}P_{16}Rb_8V_{20}$
Formula weight/(g/mol)	4364.85	4252.64
Temperature/K	100(2)	100(2)
Crystal system	tetragonal	tetragonal
Space group	$P4/mnc$	$I4/mmm$
a/Å	19.7754(7)	19.7216(8)
b/Å	19.7754(7)	19.7216(8)
c/Å	23.1729(9)	22.3083(11)
α/°	90	90
β/°	90	90
γ/°	90	90
Volume/Å^3	9062.1(7)	8676.6(8)
Z	2	2
ρ_{calc}g/cm^3	1.6	1.628
μ/mm^{-1}	13.14	13.707
F(000)	4194	4066
Crystal size/mm^3	0.1 × 0.07 × 0.06	0.06 × 0.05 × 0.03
Radiation	CuKα (λ = 1.54178)	CuKα (λ = 1.54178)
2Θ range for data collection/°	5.876 to 125.016	5.982 to 136.618
Index ranges	-21 ≤ h ≤ 21, -22 ≤ k ≤ 22, -26 ≤ l ≤ 26	-20 ≤ h ≤ 23, -18 ≤ k ≤ 23, -26 ≤ l ≤ 25
Reflections collected	53331	30037
Independent reflections	3721 [R_{int} = 0.0721, R_{sigma} = 0.0341]	2261 [R_{int} = 0.0635, R_{sigma} = 0.0329]
Data/restraints/parameters	3721/11/231	2261/3/127
Goodness-of-fit on F^2	1	1.003
Final R indexes [I>=2σ (I)]	R_1 = 0.0767, wR_2 = 0.2075	R_1 = 0.0634, wR_2 = 0.1874
Final R indexes [all data]	R_1 = 0.0834, wR_2 = 0.2123	R_1 = 0.0701, wR_2 = 0.1930
Largest diff. peak/hole / e Å^{-3}	1.07/-1.27	0.92/-1.43
CCDC Deposition No.	2350526	2350527

Supplementary Table 19. Crystallographic tables for **Na-{V_{30}}** and **K-{V_{30}}**.

Identification code	Na-{V_{30}}	K-{V_{30}}
Cryst./Empirical formula	$C_{48}H_{24}Na_{15}O_{168}P_{24}V_{30}$	$C_{48}H_{24}K_5O_{137}P_{24}V_{30}$
Formula weight/(g/mol)	5905.00	5259.65
Temperature/K	100(2)	100(2)
Crystal system	triclinic	triclinic
Space group	*P*-1	*P*-1
a/Å	19.6035(14)	19.6124(10)
b/Å	20.0402(14)	20.8088(11)
c/Å	22.0058(16)	21.2020(12)
α/°	116.134(2)	62.822(2)
β/°	92.848(3)	65.124(2)
γ/°	113.628(2)	88.747(3)
Volume/Å^3	6836.4(9)	6826.1(7)
Z	1	1
ρ_{calc}g/cm^3	1.434	1.280
μ/mm^{-1}	10.54	10.86
F(000)	2871	2553
Crystal size/mm^3	0.15 × 0.07 × 0.05	0.14 × 0.07 × 0.04
Radiation	CuKα (λ = 1.54178)	CuKα (λ = 1.54178)
2Θ range for data collection/°	4.652 to 137.69	4.884 to 137.14
Index ranges	-23 ≤ h ≤ 23<br>-24 ≤ k ≤ 24<br>-26 ≤ l ≤ 26	-23 ≤ h ≤ 23<br>-25 ≤ k ≤ 25<br>-25 ≤ l ≤ 25
Reflections collected	111396	102224
Independent reflections	25077 [R_{int} = 0.0385<br>R_{sigma} = 0.0306]	24903 [R_{int} = 0.0679<br>R_{sigma} = 0.1002]
Data/restraints/parameters	25077/94/1348	24903/46/1306
Goodness-of-fit on F^2	1.062	1.033
Final R indexes [I>=2σ (I)]	R_1 = 0.0683, wR_2 = 0.1952	R_1 = 0.0944, wR_2 = 0.2817
Final R indexes [all data]	R_1 = 0.0701, wR_2 = 0.1967	R_1 = 0.1105, wR_2 = 0.2976
Largest diff. peak/hole / e Å^{-3}	1.99/-1.09	2.54/-0.89
CCDC Deposition No.	2382883	2350530

Supplementary Table 20. Crystallographic tables for **Rb-{V_{30}}** and **Cs-{V_{30}}**.

Identification code	Rb-{V_{30}}	Cs-{V_{30}}
Cryst./Empirical formula	$C_{48}H_{24}O_{130}P_{24}Rb_4V_{30}$	$C_{48}Cs_{9.5}O_{126.5}P_{24}V_{30}$
Formula weight/(g/mol)	5294.03	6157.78
Temperature/K	100(2)	100(2)
Crystal system	monoclinic	triclinic
Space group	*C*2/*c*	*P*-1
a/Å	34.212(3)	19.8760(8)
b/Å	28.369(2)	22.6944(9)
c/Å	28.435(3)	36.6223(19)
α/°	90	94.071(3)
β/°	91.704(5)	90.680(3)
γ/°	90	115.025(2)
Volume/Å^3	27586(4)	14914.8(12)
Z	4	2
ρ_{calc}g/cm^3	1.275	1.371
μ/mm^{-1}	10.892	18.182
F(000)	10200	5791
Crystal size/mm^3	0.08 × 0.06 × 0.02	0.16 × 0.06 × 0.05
Radiation	CuKα (λ = 1.54178)	CuKα (λ = 1.54178)
2Θ range for data collection/°	4.046 to 105.226	2.422 to 113.914
Index ranges	-34 ≤ h ≤ 35<br>-29 ≤ k ≤ 29<br>-29 ≤ l ≤ 29	-18 ≤ h ≤ 17<br>-24 ≤ k ≤ 20<br>-33 ≤ l ≤ 34
Reflections collected	105045	39717
Independent reflections	15699 [R_{int} = 0.1076<br>R_{sigma} = 0.0720]	23179 [R_{int} = 0.0676,<br>R_{sigma} = 0.1328]
Data/restraints/parameters	15699/89/962	23179/3763/1825
Goodness-of-fit on F^2	1.006	0.994
Final R indexes [I>=2σ (I)]	R_1 = 0.1684, wR_2 = 0.4093	R_1 = 0.1268, wR_2 = 0.3773
Final R indexes [all data]	R_1 = 0.2027, wR_2 = 0.4403	R_1 = 0.1645, wR_2 = 0.4107
Largest diff. peak/hole / e Å^{-3}	5.30/-1.80	3.25/-1.78
CCDC Deposition No.	2350532	2350531

Supplementary References

[1] Zheng, T., Gao, Y., Chen, L., Liu, Z., Diwu, J., Chai, Z., Albrecht-Schmitt, T. E., & Wang, S. A new chiral uranyl phosphonate framework consisting of achiral building units generated from ionothermal reaction: structure and spectroscopy characterizations. *Dalton Trans.* **44**, 18158-18166 (2015).

[2] Zon, J., Videnova-Adrabinska, V., Janczak, J., Wilk, M., Samoc, A., Gancarz, R. & Samoc, M. Design, synthesis and noncentrosymmetric solid state organization of three novel pyridylphosphonic acids. *CrystEngComm*, **13**, 3474-3484 (2011).

[3] Bessmertnykh, A., Douaihy, C. M., & Guilard, R. Direct synthesis of amino-substituted aromatic phosphonates via palladium-catalyzed coupling of aromatic mono- and dibromides with diethyl phosphite. *Chem. Lett.* **38**, 738-739, (2009).

[4] SAINT+, Bruker AXS Inc., Madison, Wisconsin, USA (2012).

[5] Krause, L., Herbst-Irmer, R., Sheldrick, G. M., and Stalke, D. Comparison of silver and molybdenum microfocus X-ray sources for single-crystal structure determination. *J. Appl. Cryst.* **48,** 3-10 (2015).

[6] Sheldrick, G. M, *SHELXT* – Integrated space-group and crystal-structure determination*, Acta Cryst. A*, **A71**, 3-8 (2015).

[7] Dolomanov, O. V., Bourhis, L. J., Gildea, R. J., Howard, J. A. K., & Puschmann, H. OLEX2: A complete structure solution, refinement and analysis program. *J. Appl. Cryst.* **42**, 339-341 (**2009)**.

[8] Sheldrick, G. M. Crystal structure refinement with SHELXL,. *Acta Cryst. C* ***C71***, 3-8 (**2015)**;

[9] Spek, A. L. PLATON SQUEEZE: A tool for the calculation of the disordered solvent contribution to the calculated structure factors/ *Acta Cryst.* **C71,** 9-18 (2015).

[10] Frisch, M.J., Trucks, G.W., Schlegel, H.B., Scuseria, G.E., Robb, M.A., Cheeseman, J.R., Scalmani, G., Barone, V., Mennucci, B., Petersson, G.A., Nakatsuji, H., Caricato, M., Li, X., Hratchian, H.P., Izmaylov, A.F., Bloino, J., Zheng, G., Sonnenberg, J.L., Hada, M., Ehara, M., Toyota, K., Fukuda, R., Hasegawa, J., Ishida, M., Nakajima, T., Honda, Y., Kitao, O., Nakai, H., Vreven, T., Montgomery Jr., J.A., Peralta, J.E., Ogliaro, F., Bearpark, M., Heyd, J.J., Brothers, E., Kudin, K.N., Staroverov, V.N., Kobayashi, R., Normand, J., Raghavachari, K., Rendell, A., Burant, J.C., Iyengar, S.S., Tomasi, J., Cossi, M., Rega, N., Millam, J.M., Klene, M., Knox, J.E., Cross, J.B., Bakken, V., Adamo, C., Jaramillo, J., Gomperts, R., Stratmann, R.E., Yazyev, O., Austin, A.J., Cammi, R., Pomelli, C., Ochterski, J.W., Martin, R.L., Morokuma, K., Zakrzewski, V.G., Voth, G.A., Salvador, P., Dannenberg, J.J., Dapprich, S., Daniels, A.D., Farkas, O., Foresman, J.B., Ortiz, J.V., Cioslowski, J. and Fox, D.J. Gaussian 09, Revision D.01 (2009).

[11] (a) Perdew, J. P., Burke, K., & Ernzerhof, M. Generalized Gradient Approximation Made Simple, *Phys. Rev. Lett.* **77**, 3865-3868 (1996); (b) Adamo, C., & Barone, V., Toward reliable density functional methods without adjustable parameters: The PBE0 model. *J. Chem. Phys.*, **110**, 6158-6170 (1999).

[12] Dolg, M., Wedig, U., Stoll, H., & Preuss, H. Energy-adjusted ab initio pseudopotentials for the first row transition elements, *J. Chem. Phys.*, **86**, 866-872 (1987).

[13] Francl, M. M., Pietro, W. J., Hehre, W. J., Binkley, J. S., Gordon, M. S., Defrees, D. J., & Pople, J. A., Self-consistent molecular orbital methods. XXIII. A polarization-type basis set for second-row elements *J. Chem. Phys.* **77**, 3654-3665 (1982).

[14] Tandon, S., Soriano-López, J., Kathalikkattil, A. C., Jin, G., Wix, P., Venkatesan, M., Lundy, R. Morris, M. A., Watson, G. W., Schmitt, W., A cubane-type manganese complex with H_2O oxidation capabilities, *Sustain. Energy Fuels*, **4**, 4464-4468 (2020).

[15] Tandon, S., Venkatesan, M., Schmitt,W. Watson, G. W., Altering the nature of coupling by changing the oxidation state in a {Mn_6} cage, *Dalton Trans.*, **49**, 8086-8095 (2020).

[16] Micera, G., Garribba, E., The effect of the functional, basis set, and solvent in the simulation of the geometry and spectroscopic properties of $V^{IV}O^{2+}$ complexes - Chemical and biological applications, *Int. J. Quantum Chem.*, **112**, 2486-2498 (2012).

[17] Cramer, C. J., Truhlar, D. G., Density functional theory for transition metals and transition metal chemistry, *Phys. Chem. Chem. Phys.*, **11**, 10757-10816(2009)

[18] Breen, J. M., Schmitt, W. Hybrid organic-inorganic polyoxometalates: functionalization of V(IV)/V(V) nanosized clusters to produce molecular capsules. *Angew. Chem. Int. Ed.*, **47**, 6904-6908 (2008).

[19] Mahimaidoss, M. B., Krasnikov, S. A., Reck, L., Onet, C. I., Breen J. M., Zhu N., Marzec B., Shvets I. V., Schmitt W. Homologous size-extension of hybrid vanadate capsules – solid state structures, solution stability and surface deposition, *Chem. Commun,* **50**, 2265-2267 (2014).

[20] Breen J. M., Clérac R., Zhang L., Cloonan S. M., Kennedy E., Feeney M., McCabe T., Williams D. C., Schmitt W. Self-assembly of hybrid organic-inorganic polyoxovanadates: functionalized mixed-valent clusters and molecular cages, *Dalton Trans.* **41**, 2918-2926 (2012).

[21] Altermatt, D. & Brown, I. D. The automatic searching for chemical bonds in inorganic crystal structures. *Acta Cryst*. **B41**, 240-244 (1985).

[22] Brese, N. E. & O'Keeffe, M. Bond-valence parameters for solids. *Acta Cryst.* **B47**, 192-197 (1991).

[23] Brown, I. D. Recent developments in the methods and applications of the bond valence model. *Chem. Rev.* **109**, 6858-6919 (2009).

[24] Pauling, L. The principles determining the structure of complex ionic crystals. *J. Am. Chem. Soc.*, **51**, 1010-1026 (1929).

[25] Müller, A., Hovemeier, K., Krickemeyer, E. & Bögge, H. Modeling the Remote-Controlled Organization of Particles in a Nanodimensional Cavity: Synthesis and Properties of$(Et_3NH)_3(tBuNH_3)_2$-$Na_2[(H_2O)_2,N^{-3} \subset V_{14}O_{22}(OH)_4(PhPO_3)_8]\cdot 6H_2O\cdot 2DMF$. *Angew. Chem. Int. Ed.* **34**, 779-781 (1995).

[26] Nakamoto, K. in *Infrared and Raman Spectra of Inorganic and Coordination Compounds Part A*, John Wiley & Sons, Inc., Hoboken, New Jersey (2008).

[27] Xiao, L.-N. *et al.* Synthesis and characterizations of the first $[V_{16}O_{39}Cl]^{6-}$ ($V_{16}O_{39}$) polyanion. *Dalton Trans.*, **42**, 5247-5251 (2013).

[28] Daniel, C. & Hartl, H. Neutral and cationic V(IV)/V(V) mixed-valence alkoxo-polyoxovanadium clusters $[V_6O_7(OR)_{12}]^{n+}$ (R = $-CH_3$, -C2H_5): structural, cyclovoltammetric and IR-spectroscopic investigations on mixed valency in a hexanuclear core. *J. Am. Chem. Soc.*, **127**, 13978-13987 (2005).

[29] Keene, T. D. *et al.* $V_{16}O_{38}(CN)$]9–: A Soluble Mixed-Valence Redox-Active Building Block with Strong Antiferromagnetic Coupling, Inorg. Chem. **51**, 17, 9192–9199 (2012).
